# Age-Related Variations and Seasonal Influences: A Network Analysis of Comorbidities in Asthma Hospitalizations (2013–2023)

**DOI:** 10.3390/jcm14072350

**Published:** 2025-03-29

**Authors:** Ana Adriana Trusculescu, Versavia Maria Ancusa, Alexandra Burducescu, Camelia Corina Pescaru, Daniel Trăilă, Norbert Wellmann, Ovidiu Fira-Mladinescu, Cristian Iulian Oancea

**Affiliations:** 1 Center for Research and Innovation in Personalized Medicine of Respiratory Diseases (CRIPMRD), Pulmonology University Clinic, ‘Victor Babes’ University of Medicine and Pharmacy, Eftimie Murgu Square No. 2, 300041 Timisoara, Romania; ana.trusculescu@umft.ro (A.A.T.); pescaru.camelia@umft.ro (C.C.P.); traila.daniel@umft.ro (D.T.); norbert.wellmann@umft.ro (N.W.); oancea@umft.ro (C.I.O.); 2 Pulmonology University Clinic, Clinical Hospital of Infectious Diseases and Pneumophysiology, Dr. Victor Babeș Timișoara, Gheorghe Adam Street, No. 13, 300310 Timisoara, Romania; alexandra.burducescu.umfvbt@gmail.com; 3 Department of Computer and Information Technology, Automation and Computers Faculty, “Politehnica” University of Timis, Vasile Pârvan Blvd, No. 2, 300223 Timisoara, Romania; 4 Doctoral School, ‘Victor Babes’ University of Medicine and Pharmacy, Eftimie Murgu Square 2, 300041 Timisoara, Romania

**Keywords:** asthma, comorbidities, age-related patterns, seasonal variations, complex network analysis

## Abstract

**Background:** Asthma, a chronic respiratory disease characterized by airway inflammation and hyperresponsiveness, exhibits significant heterogeneity in its presentation. This study aimed to investigate age-related comorbidity patterns, seasonal variations, and demographic trends among a cohort of asthma patients within a defined geographical region. **Methods:** A retrospective analysis of 13,695 asthma patients admitted to a Romanian hospital from 2013 to 2023 was conducted. Comorbidity patterns were analyzed using network analysis across age groups, and seasonal trends were investigated through spectral analysis. **Results:** Asthma admissions exhibited non-linear trends with female predominance (57.72%). The pandemic significantly impacted admission rates, with males experiencing greater COVID-19-related effects. Female admissions showed distinct seasonal patterns potentially linked to domestic responsibilities. Comorbidity patterns evolved with age, shifting from lifestyle factors in younger patients to complex cardiovascular and neurological disorders in older groups. The 60–69 age group showed the highest integration of comorbidity communities. **Conclusions:** The study revealed that asthma management focus should shift with patient age from the disease itself to addressing underlying comorbidities. Understanding these complex patterns may help personalize treatment strategies and improve long-term prognosis for asthma patients.

## 1. Introduction

Asthma, a heterogeneous disease, affects approximately 260 million people globally and causes 455,000 deaths annually, according to WHO estimates [1,2]. In the EU, asthma prevalence averages 6%, with Finland reporting the highest rate at 9%, followed by Germany and France, while Romania and Bulgaria have the lowest rates, under 2% [3]. Gender differences are evident: boys have a higher prevalence in childhood, shifting to adult women post-puberty [4]. Despite treatment advances, preventable deaths persist due to factors like suboptimal medication use and limited healthcare access [5,6].

Asthma phenotypes are broadly categorized into two groups: early-onset allergic asthma (childhood development, Th2-driven inflammation, linked to atopic diseases like allergic rhinitis/eczema) [7] and adult-onset asthma (neutrophilic inflammation, associated with obesity/respiratory infections, more severe with frequent relapses and fixed airflow limitation despite better lung function) [7,8]. Severity factors differ, where childhood asthma is influenced by neutrophilic profiles, lung function, and medication use, while adult severity relates to elevated IgE, eosinophilia, smoking, and obesity [9].

Asthma comorbidities, often overlooked, significantly impact disease progression. These comorbidities vary by age, gender, and socioeconomic factors, affecting asthma outcomes and management. The prevalence of comorbidities and related specialty conditions in the asthma population tends to be higher than in the general population [7]. We can divide comorbidities into two groups: pulmonary (obstructive sleep apnea (OSA) 49.5% [10], allergic rhinitis and chronic rhinosinusitis (nasal disease)—approximately 95% [11]; vocal cord dysfunction and chronic obstructive pulmonary disease (COPD)—23.3% [12]; bronchiectasis—36.6% [13]) and extrapulmonary (gastroesophageal reflux disease (GERD)—18–25% [14]; cardiovascular disease—25% [15]; obesity—29% [16]; diabetes mellitus—11.2% [17]; anxiety/depression—36% [18]). However, the early-onset asthma population is prone to atopic conditions such as allergic rhinitis and atopic dermatitis, and the adult-onset asthma population is prone to obesity, metabolic syndrome, and cardiovascular diseases [19]. Older adults (late-onset asthma) have a higher prevalence of obesity, dyslipidemia, arthritis, diabetes, hypertension, and an increased likelihood of COPD with a younger age of asthma diagnosis [20]. The older group is characterized by systemic inflammation, sustained by adipose tissue, which can worsen asthma disease and its responsiveness to treatment [7]. Half of the population suffering from obesity and overweight also has asthma. Obesity exacerbates asthma by altering inflammatory pathways through proinflammatory adipokines. This condition is also linked to corticosteroid resistance, potentially reducing treatment efficacy [14]. Recognizing conditions associated with asthma, such as rhinitis, sinusitis, and GERD, is crucial as they can mimic asthma symptoms like persistent cough and chest tightness. Misdiagnosing these shared symptoms as uncontrolled asthma may lead to unnecessary medication escalation. Instead, effectively managing these comorbidities can significantly improve asthma control [11].

Socioeconomic factors greatly affect patients’ access to safe housing, nutritious food, and quality healthcare. Poor and minority populations often experience worse health outcomes, particularly for allergic and immunologic conditions [21].

Low-income Asian and African nations report higher asthma mortality (16.43 and 9.10 per 100,000) but lower prevalence (30.23%, 7.88%) versus high-income countries (62.69%, 57.13%) [22]. Limited healthcare access and education likely contribute to these disparities. In the U.S., Black and Latinx populations face elevated asthma rates due to socioeconomic inequities, environmental exposures, and poor healthcare access [21,23].

Asthma exacerbations peak in autumn and winter (4.66%, 5.12%), likely linked to respiratory viral infections. Studies demonstrate that ICS/LABA combinations outperform ICS monotherapy in reducing these events, suggesting pre-cold season step-up therapy for high-risk patients [24].

In this study, we aim to analyze age, sex, seasonal, and comorbidity trends in asthma patients in Timis County and investigate how comorbidity network groups change depending on patients’ age, what could determine these changes, and their impact on disease management. The study’s primary goal is to understand factors influencing hospital admissions, which indirectly inform asthma management and heterogeneity by analyzing comorbidities and demographic trends.

Although existing research has delineated the global epidemiology of asthma, its demographic variations, and the clinical significance of comorbid conditions, there remains a paucity of studies investigating localized populations with shared genetic and lifestyle characteristics and their influence on asthma presentation. Such context-specific investigations, as emphasized by the Global Initiative for Asthma (GINA) guidelines, are paramount for developing effective, personalized, and geographically tailored management strategies. Our study addresses this need by conducting a comprehensive long-term analysis over 11 years, providing valuable insights into a nuanced understanding of how asthma presentation evolves over time, as well as highlighting the importance of localized research for improving asthma management and outcomes in specific populations.

## 2. Materials and Methods

This retrospective study (1 January 2013–30 November 2023) analyzed data from 13,695 patients with an asthma diagnosis admitted to the “Victor Babes” University Hospital in Timisoara, Romania. Asthma was defined according to the Global Initiative for Asthma (GINA) guidelines. The medical data were extracted from the healthcare facility’s digital patient record system. Only inpatients (12 h of hospitalization or longer) were included because the medical dates for outpatients were inconsistent for such a long analysis period. All participants have written informed consent for research purposes.

This retrospective study included patients aged 18 or older with a discharge diagnosis of asthma (ICD-10 code J45 and sub-codes). Patients were selected from complete medical records available between 1 January 2013, and 30 November 2023, and had to reside in Timis County, Romania. Exclusion criteria were age < 18 years, unknown home address, address not in Timis County and no informed consent obtained.

The study received ethical approval from the appropriate ethics committees, numbers 38/24.11.2023 (university) and 10535/13.11.2023 (hospital).

A linear regression analysis was performed to assess the trend of yearly admissions. The goodness of fit was evaluated using the R-squared value. The overall average admission rate was compared to the pre-COVID-19 average to assess the pandemic’s impact. Gender distribution was analyzed by calculating the proportion of female and male admissions. A chi-squared test was used to determine the significance of the difference in gender proportions across the entire study period. To examine the shift in gender distribution during the COVID-19 pandemic, specifically between 2020 and 2021, a Mann–Whitney U test was performed. Statistical significance was set at *p* < 0.05. All statistical analyses were conducted using SciPy 1.14.1.

To identify seasonal patterns in asthma prevalence, spectral analysis was performed on time-series data of monthly asthma hospital admissions, split into males and females. The time-series spanned the whole study period. Seasonal decomposition was conducted using the seasonal_decompose function from the statsmodels.tsa.seasonal module in Python 3.11.11. An additive model was employed to separate the time-series data into trend, seasonal, and residual components. The seasonal component, representing cyclical variations within a 12-month period, was extracted and analyzed to understand the influence of periodic factors on asthma prevalence. All spectral analyses were performed using Python, with the statsmodels library version 0.14.4.

Data analysis was conducted using Python programming language, with panda 2.2.2, NumPy 2.0.2, SciPy 1.14.1, StatsModels 0.14.4, matplotlib 3.10.0 and seaborn 0.13.2 libraries. To clean up the data, the anonymized hospital database was parsed into a dataframe and the diagnoses were reduced to a list of ICD-10 codes. Only patients that presented a code classified as asthma were analyzed. To minimize registrar bias, all J45 (asthma) subcodes were merged. Age was calculated from the birthdate on file and extracted as full years of life.

To analyze comorbidities, each patient’s ICD10 discharge codes (regardless of whether asthma was the primary or secondary discharge diagnostic) were transformed into a fully connected network. Repeated admissions were handled by considering each admission as a separate event for the purpose of this analysis. The patients’ networks were aggregated according to age groups into larger ones. In constructing the network graphs, we encountered the challenge of representing numerous ICD-10 codes without compromising visual clarity. Given the number of unique codes per network revolved around 1000, we focused on highlighting the ones that create the most meaningful links between comorbidities. However, in order to provide all the information, we included a Supplementary Table S1 that lists all nodes as ICD-10 codes and their measured values in the network, as well as the general asthma comorbidities’ complex network characteristics for each age range in Sections S1 and S2. Gephi 0.10.1 was used to compute communities and node influence (betweenness centrality (BC)) values, normalizing it in the [0, 1] interval, for objective comparison between the networks. The visual representation was rendered with a force-directed layout, to maximize community grouping, with nodes colored according to their mathematical affinities. Node sizes were scaled proportionally to their mathematically derived centrality measures, reflecting their influence within the network, with larger nodes indicating higher centrality values. Label size was proportional to node size. This allowed us to focus on the most relevant information in very dense networks. The same color scheme was used for all the networks, to facilitate visual comparison.

Clinicians further refined the clusters by grouping related disease codes into categories. The relative influence of these categories was calculated as the sum of their individual BC values divided by the BC value of the asthma code itself.

## 3. Results

The asthma cohort, summarized in Table 1, presented a mean age of 59.6 ± 14.8 years, with a female predominance of 57.2%. The average length of hospital stay was 4.5 ± 5.9 days. Age distribution was relatively uniform across adult categories, with the largest group being the 60–69 age group (28.8%). Temporal analysis revealed a fluctuating distribution of cases over the years, peaking in 2018 (12.8%) and reaching a nadir in 2021 (5.0%).

**Figure 1:**
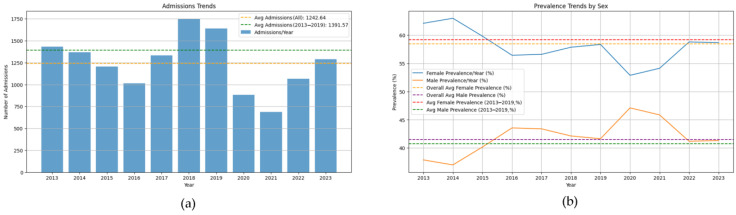
Asthma hospital admissions (2013–2023) showing annual trends and gender differences: (**a**) annual admission numbers and averages (overall and pre-pandemic); (**b**) female–male prevalence percentage.

Yearly total admissions (Figure 1a) do not follow a linear trend for hospitalizations (R-squared = −13.16 <0 for linear regression fit). Admission rates over the study period (overall average) compared to the pre-COVID-19 average asthma patient hospitalization rates are lower, highlighting the pandemic-impacted asthma admissions. The post-pandemic period shows a continued deviation from pre-pandemic levels to a significant decrease in admissions, reflected in the shifted average value, probably due to reduced respiratory infections and public health measures. In general, females represented a significantly higher proportion of total admissions (57.72%, *p* < 0.001) compared to males, (Figure 1b). A significant shift in gender distribution was observed between 2020 and 2021, during the pandemic, with a decrease in the proportion of female patient admissions (*p* = 0.018 < 0.05, Mann–Whitney U test). Females exhibit a higher prevalence of asthma hospitalizations with distinct seasonal patterns, potentially influenced by hormonal factors or domestic responsibilities, particularly during winter. In contrast, males show a different trend, with increased admissions during the COVID-19 pandemic, reaching almost an equal percentage with the female admissions, possibly due to heightened susceptibility to severe infections.

**Figure 2:**
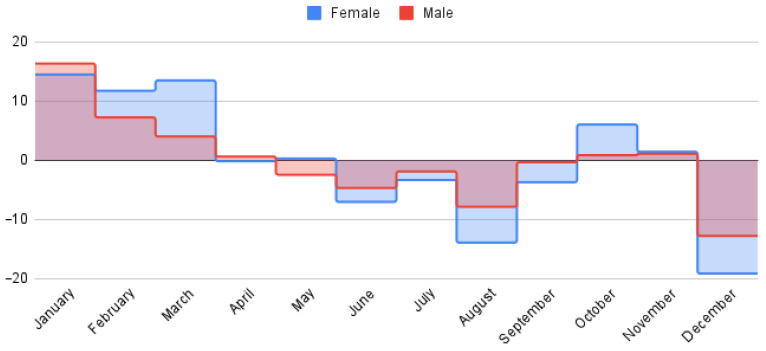
Spectral analysis of seasonal asthma variations using a 12–month periodicity for males and females.

Seasonal patterns were discerned using spectral analysis. Out of the three components, to understand the influence of periodic factors on asthma prevalence, we selected the seasonal component, which represents cyclical variations occurring within 12 months. Figure 2 illustrates the seasonal patterns of asthma prevalence throughout the year, as determined by spectral analysis of monthly time-series data. The *Y*-axis represents the deviation from the average monthly prevalence, expressed as a percentage. Positive values indicate months where asthma prevalence was higher than the average, while negative values indicate months where prevalence was lower than the average.

The magnitude of the values reflects the strength of the seasonal effect. Larger positive or negative values indicate a greater deviation from the average, suggesting a more pronounced seasonal influence. By examining the patterns across the year, we can identify periods of increased or decreased asthma prevalence, potentially related to factors such as weather changes, allergens, or respiratory infections

**Figure 3:**
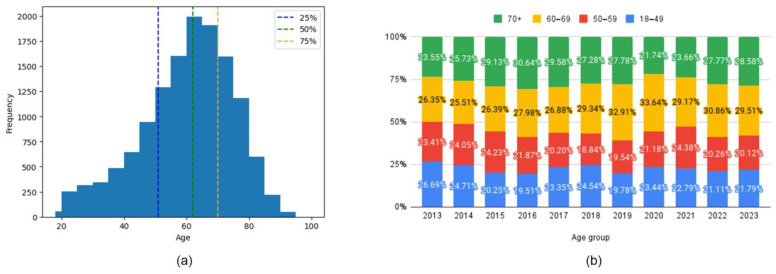
Age distribution and yearly trends: (**a**) age description histogram and (**b**) age group yearly variations.

Patient age distribution had a mean of 59.62 years, with quartiles at 51, 62, and 70 years, respectively (Figure 3a). To enhance analytical granularity, age groups were defined based on quartiles of the age distribution. Particular attention was given to the 50- and 60-year age thresholds to facilitate comparison with existing literature (Figure 3b).

**Figure 4:**
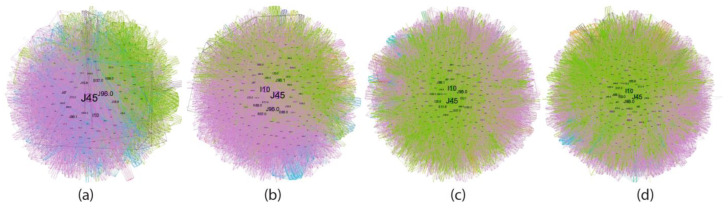
Evolution of asthma comorbidities (using ICD-10 discharge codes) in age groups (**a**) 18–49; (**b**) 50–59; (**c**) 60–69; (**d**) 70+.

**Figure 5:**
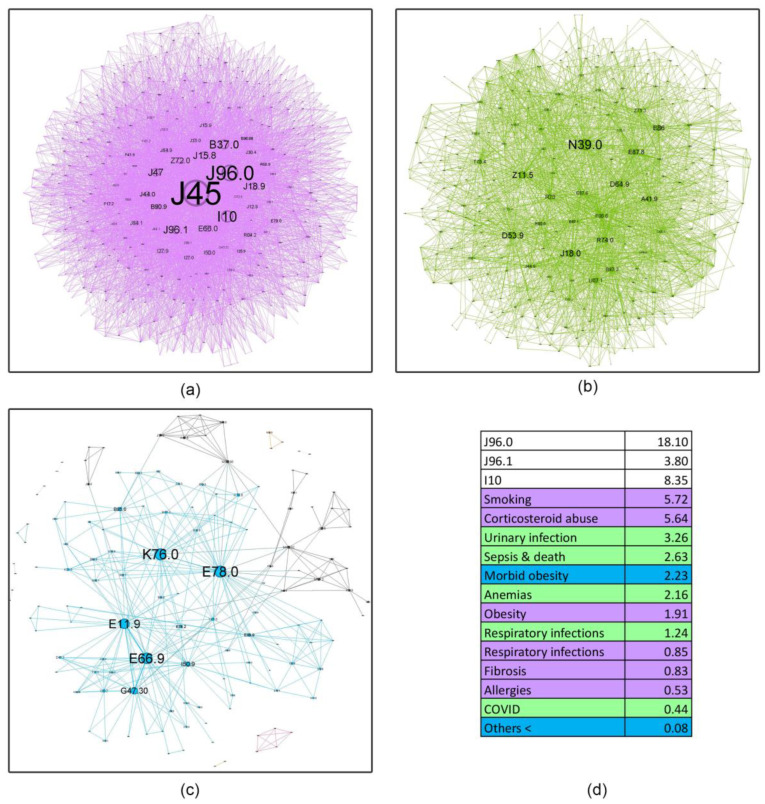
Asthma ICD-10 codes’ community network structures for the 18–49 age group. Subfigures (**a**–**c**) represent main, secondary, and communities’ sizes, while (**d**) colors show the main disorders categories and their relative percentage. (**a**) PURPLE: smoking (Z72.0, J43.9), oral candidosis (B37.0, B37.1, B37.7!, J31.0), obesity (E11.9, E66.0, G47.32, R73, E78.5), respiratory infections (J15.8, J18.9, J47, J15.9, J15.6, J18.8, B90.9, A15.0, J15.2, J15.1, J15.0), lung fibrosis (J84.1, 9); allergies (J30.4, J30.1). (**b**) GREEN: urinary disorders (N39.0, N40, N18.90, N20.9, N02.9), sepsis and death (E87.6, A41.9, E87.1, E87.8, I46.9, E86, E87.5, E87.2, E87.0), anemia (D53.9, D64.8, D63.8*, D50.8), respiratory infections (J15.8, J18.9, J47, J15.9, J15.6, J18.8, B90.9, A15.0, J15.2, J15.1, J15.0); COVID-19 (U07.1) (**c**) BLUE: morbid obesity (E66.9, K76.0).

**Figure 6:**
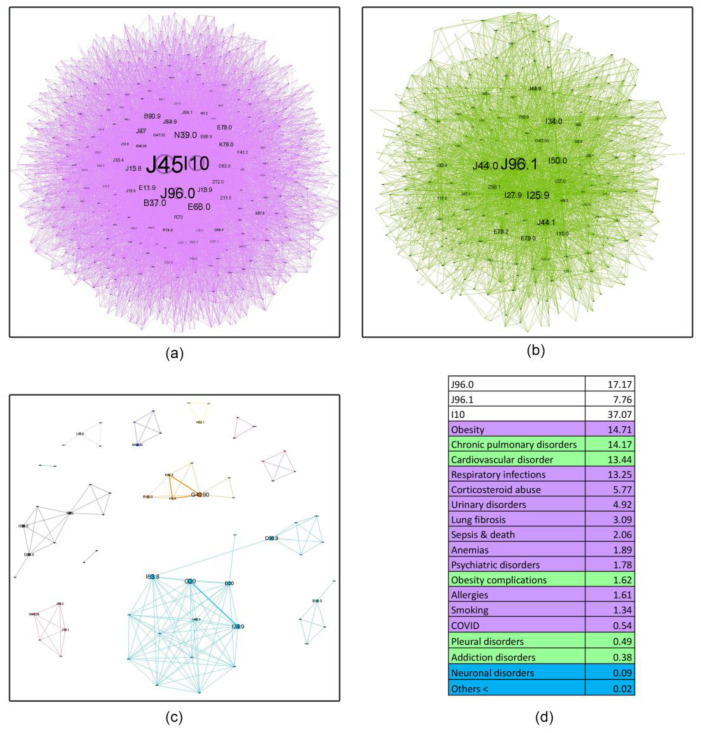
Asthma ICD-10 codes’ community network structures for the 50–59 age group. Subfigures (**a**–**c**) represent main, secondary, and small communities’ sizes, while (**d**) colours show the main disorders categories and their relative percentage. (**a**) PURPLE: obesity (E66.0, E78.0, K76.0, R73, E66.9, E11.9, G47.32), candidiasis (B37.0, B37.1, J31.0), urinary (N39.0), allergy (J30.1, J30.4), smoking (Z72.0, J43.9), respiratory infections (J15.8, B90.9, J18.9, Z11.5, J15.9, J47, J12.9, J18.0), COVID-19 (U07.1), psychiatric disorders (F41.2, F06.6, F41.9, F33.9), pulmonary fibrosis (J84.1, J84.9), sepsis (E87.1, E87.6, E87.8, A41.9); anemia (D53.9, D64.9, D50.8). (**b**) GREEN: chronic pulmonary disorders (ACO) (J96.1, J44.0, J44.1, J44.9, Z99.1, J44.8, J42, J41.0, J94.8, J43.8), pleural disorder (J92.9), cardiovascular disorders (I25.9, I50.0, I27.9, I34.0, E79.0, I11.0, I27.0, I50.9, I47.1, I48, I36.1, I20.9, I45.0, I07.1, I70.0, I20.8, I25.2, I49.3, I30.9, I27.2, I26.9, I70.8, I35.1), obesity complications (E78.2, E78.5, G47.30, E11.8); addition disorders (alcohol and smoking) (F17.2, F10.2). Note: There is no direct causal link established between asymptomatic hyperuricemia and respiratory disorders. The primary concerns with hyperuricemia are related to gout, kidney stones, and other metabolic and cardiovascular issues. (**c**) BLUE: neuronal disorders (G20, I63.8, I24.9).

**Figure 7:**
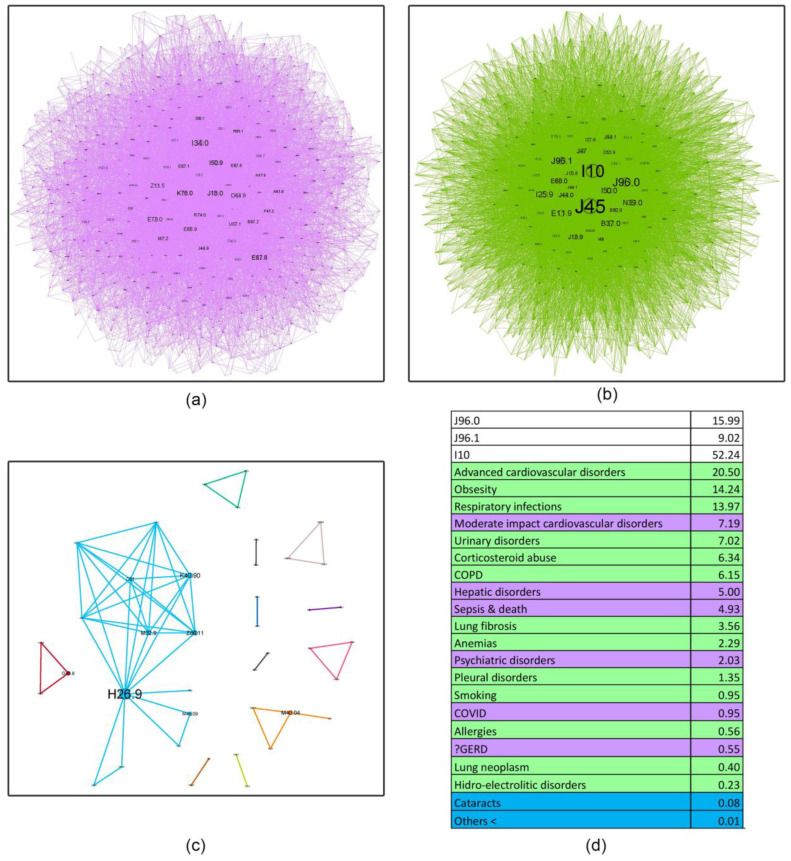
Asthma ICD-10 codes’ community network structures for the 60–69 age group. Subfigures (**a**–**c**) represent main, secondary, and communities’ sizes, while (**d**) colours represent the main disorders categories and their relative percentage. (**a**) PURPLE: moderate-impact cardiovascular disorders (I34.0, I50.9, I36.1, I07.1, I25.2, I35.1, I20.8), death and sepsis (E87.8, E87.1, A41.9, I46.9, E86, E87.6), hepatic disorders (K76.0, R74.0, B18.1, B18.2, E78.0), COVID-19 (U07.1), psychiatric disorders (F41.2, F41.9, F06.6, F33.9); GERD (K21.0, K21.9). (**b**) GREEN: obesity (E11.9, E66.0, E11.8, G47.32, R73, E78.2, G47.30), advanced cardiovascular disorders (I25.9, I27.9, I48, E79.0, I50.0, I27.0, I11.0, I20.9, I25.5, I70.0, I45.0, I25.8, I49.3, I11.9, I47.1, I27.8, R00.0), oral candidiasis (B37.0, B37.1, J31.0), urinary disorders (N39.0, N18.90, N40, R31), pleural disorders (J90, J92.9), COPD (J44 + Z99.1), lung fibrosis (J84.1, J84.9), respiratory infections (J47, J18.9, J15.8, J15.9, B90.9, J12.9), allergies (J30.4), smoking (Z72.0, F17.2), anemia (D53.9, D64.8, D50.8), lung cancer (C34.9, R59.0); hydro-electrolytic disorders (A41.8, E87.0), (**c**) BLUE: cataracts (H26.9).

**Figure 8:**
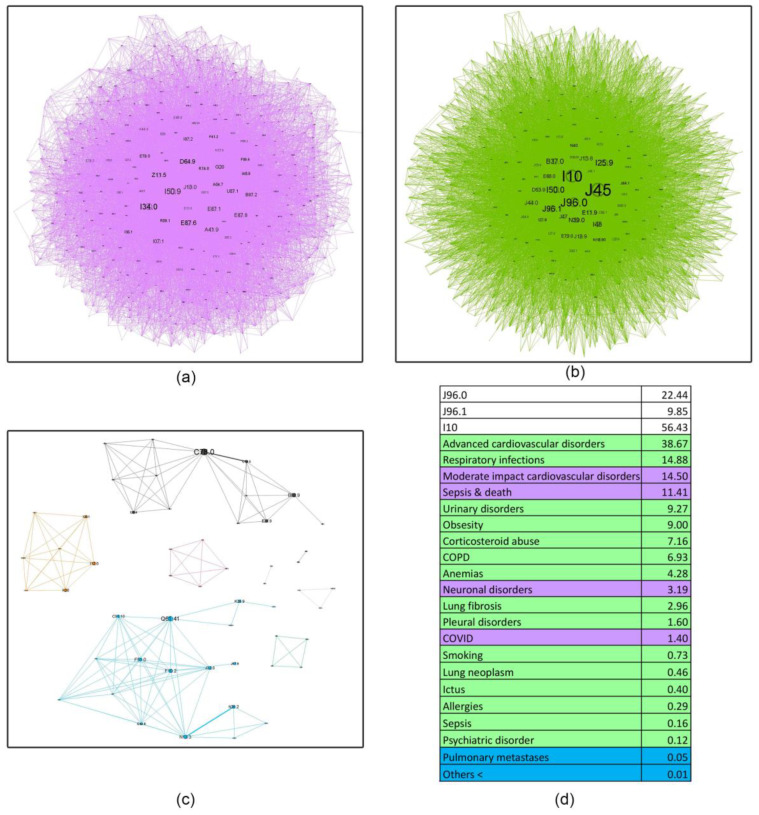
Asthma ICD-10 codes community networks structures for the 70+ age group. Subfigures (**a**–**c**) represent main, secondary, and communities’ sizes, while (**d**) colours represent the main disorders categories and their relative percentage (**a**) PURPLE: moderate-impact cardiovascular disorders (I34.0, I50.9, I07.1, I35.1, I36.1, I70.0, I27.2, I35.0, I70.8, I26.9, I44.0, I50.1, I45.1, I25.0, I49.8), sepsis and death (E87.6, A41.9, E87.1, E87.8, I46.9, E86, E87.5, E87.2, E87.0), neuronal disorders (G20, G31.0, I63.9, I63.8, R42, I69.3, F03, F06.7); COVID-19 (U07.1) (**b**) GREEN: moderate-impact cardiovascular disorders (I50.0, I25.9, I27.9, I48, I11.0, E79.0, I44.7, I20.8, I45.0, I70.9, I20.9, I27.0, I25.5, I11.9, I25.2, J12.9, I42.0, I25.6, I49.1, I25.8, I47.1, I35.2, I49.3, I27.8, I20.0, I49.4), oral candidosis (B37.0, B37.1, B37.7!, J31.0), COPD (J44 + Z99.1), lung fibrosis (J84.1, 9), respiratory infections (J15.8, J18.9, J47, J15.9, J15.6, J18.8, B90.9, A15.0, J15.2, J15.1, J15.0), obesity (E11.9, E66.0, G47.32, R73, E78.5), urinary disorders (N39.0, N40, N18.90, N20.9, N02.9), pleural disorders (J92.9, J92.0, J90, J94.8), anemia (D53.9, D64.8, D63.8*, D50.8), allergies (J30.4, J30.1), cancer (C34.9, R59.0, C34.1, C34.8, C34.0), psychiatric disorders (F41.2, F41.9, F06.6, F33.9), smoking (Z72.0, J43.9); ictus and sepsis (E87.1, E87.6, E87.8, A41.9). (**c**) BLUE: pulmonary metastasis (C78.0).

Complex network analysis was employed to investigate the age-related evolution of asthma comorbidities co-occurrence patterns within these cohorts (Figure 4, Figure 5, Figure 6, Figure 7 and Figure 8). Figure 4 presents an overview of the network across the studied age range of how a comorbidity community blends (main community is purple, second is blue and third green) in relation to asthma diagnosis over time. The largest comorbidity community, denoted by purple, comprises a cluster of comorbidities that are strongly interconnected, and the probability that ICD-10 codes to meet is high. In contrast, the blue and green communities exhibit gradually weaker connections, although links between ICD codes still exist. These communities were identified through complex network analysis, which leverages the connections between ICD codes to group related comorbidities. Network analysis revealed significant age-related variations in comorbidity patterns among asthma patients. The intensity of connections between comorbidities decreases from the youngest age group (18–49 years) to the oldest (70+ years), as illustrated in Figure 4a–d. This trend is accompanied by a gradual shift in the color distribution, with a predominance of green in the older age groups (Figure 4c,d), reflecting a shift in the complexity and interrelatedness of comorbidities with advancing age.

The analysis of diagnostic diversity revealed a progressive increase in the number of unique diagnoses across age cohorts: 956 in the 18–49 age group, 996 in the 50–59 group, 1266 in the 60–69 group, and 1289 in the 70+ group. A comprehensive list of diagnoses for each age group is provided in Section S2.

Figure 5, Figure 6, Figure 7 and Figure 8 provide a nuanced analysis of the comorbidity clusters identified in Figure 4, offering detailed insights into the evolution of purple, green, and blue communities across different age groups.

The purple cluster, prevalent in younger age groups (18–49 years), in Figure 5 is characterized by lifestyle factors such as smoking and obesity, which gradually lose influence as age increases. In contrast, the green cluster, comprising cardiovascular and metabolic disorders, becomes more prominent in older age groups (50–59, 60–69 and 70+ years), as shown in Figure 6, Figure 7 and Figure 8, reflecting a shift towards chronic conditions. The blue cluster, representing neurological and psychiatric disorders, shows increased integration with other comorbidities in elderly patients, underscoring the complexity of health conditions in this demographic. In Figure 7, the 60–69 age group showed the highest integration of comorbidity communities, indicating a critical period for managing multiple health conditions simultaneously.

The intricate interplay of multiple coexisting medical conditions and their age-dependent manifestations using complex networks of comorbidity patterns change across age groups (18–49, 50–59, 60–69, 70+), as summarized in Table 2. Age-specific considerations include the following:

For 18–49 years: A focus on modifiable lifestyle factors such as tobacco smoking cessation, obesity reduction, preventing respiratory infections (flu vaccinations) and allergy management.

For 50–59 years: A focus on the increased prevalence of cardiovascular diseases (low-dose aspirin, statins if needed), chronic obstructive pulmonary disease (smoking cessation, pulmonary rehabilitation on top of normal pharmacotherapy), and susceptibility to severe infections (vaccination, lifestyle).

For 60–69 years: A pivot approach towards the higher incidence of moderate cardiovascular disorders (medication management and lifestyle changes), hepatic dysfunction (screening and early detection, as well as alcohol consumption management), and psychiatric conditions (specific screening as well as social support). Sepsis emerges as a significant clinical concern, highlighting the importance of prompt treatment as well as education of symptoms to primary caregivers and patients.

For 70+ years: A focus on advanced cardiovascular diseases (requiring complex medication management, blood pressure control and even specific procedures like transcatheter aortic valve implantation or intravascular imaging-guided PCI), neurological disorders (addressed through cognitive health monitoring), and a heightened risk of sepsis and mortality, requiring comprehensive care strategies that consider comorbidities, frailty, and individual patient needs.

## 4. Discussion

### 4.1. Seasonal and Demographic Trends

Yearly trends (Figure 1a) are consistent with literature findings indicating a decline in asthma hospital admissions during the COVID-19 pandemic, associated with a decrease in respiratory infections, which would have triggered asthma exacerbation [25]. Even though multiple studies show a reduction in asthma-related admissions, the reasons behind these findings are diverse and require more research. One explanation could be a decrease in overall air pollution (an environmental trigger) during lockdown due to fewer working factories and less car transportation [26]. Another explanation involves the impact of public health measures, such as wearing a mask and social distancing, which lowered the cases of all air-transmitted respiratory infections [27]. Nevertheless, some articles state that patient disease management could have been poorer in those years without professional and patient-group support, and patients were experiencing worsened respiratory symptoms [28], but did not seek medical attention due to concerns about healthcare facilities being potential sources of COVID-19 transmission and the contradictory messages between guidance on accessing health services and lockdown restrictions [29]. Although on a rising trend, current admission numbers fail to reach the previous levels. This warrants further investigation to determine the cause: altered patient behavior or underlying shifts in asthma epidemiology.

The increase in the male patient population during 2020–2021 (Figure 1b) correlates with the literature description of males with asthma developing worse COVID-19 forms [30,31]. The same studies highlight the potential vulnerability of older adults and the rise in admissions for the combined 50–69 age group (54.81% and 53.56% in pandemic years), and Figure 3b confirms this association. Further interdisciplinary (psychology, sociology) investigation is warranted to explore why the trend is reversed in the 70+ age group. A plausible explanation could be that families protected their elderly relatives by performing strict social distancing measures, keeping them out of contact with the outside world, and ensuring that older adults adhere better to their medication during this period.

The seasonal variations in asthma admissions, as depicted in Figure 2, reveal distinct patterns between male and female patients, aligning with established literature indicating higher prevalence during winter and lower prevalence during summer [24,32]. While both sexes exhibit elevated admission rates during the winter months (January–March), a divergence is observed, where male admissions demonstrate a descending trend, whereas female admissions remain relatively stable. Several potential confounding factors warrant consideration in explaining this disparity.

Firstly, the hypothesis that indoor air quality serves as a primary driver of seasonal asthma exacerbations must be addressed. During winter, increased time spent indoors, coupled with the use of heating systems, limited ventilation, and heightened utilization of cleaning agents, may contribute to compromised indoor air quality for all individuals, not exclusively females. This factor could independently explain the observed seasonal patterns, irrespective of domestic responsibilities.

Secondly, the prevalence of viral infections during winter, a known trigger for asthma exacerbations, presents another potential confounder. The observed increase in asthma admissions could be attributed to the heightened incidence of colds and influenza, rather than gender-specific domestic activities.

Thirdly, the role of hormonal fluctuations in female asthma severity cannot be overlooked. Seasonal changes may influence hormonal patterns, which, in turn, could directly impact asthma symptom expression, independent of domestic exposures.

Fourthly, weather patterns, characterized by variations in humidity and temperature, may directly influence asthma exacerbations. These environmental factors could explain the seasonal trends observed, independent of gender or domestic activities.

Fifthly, access to healthcare may be seasonally influenced, potentially affecting the reporting and management of asthma symptoms. A decline in healthcare access during specific seasons could lead to an apparent increase in reported asthma symptoms, rather than reflecting true disease prevalence.

While national statistical studies suggest that females spend more time indoors, engaging in domestic duties, and thus potentially experiencing increased exposure to indoor air pollutants such as those associated with cooking [33,34,35] and temperature differentials when going outside [36]. These observations may be confounded by the aforementioned factors. Additionally, the potential influence of sex hormones on female asthma pathology, as suggested by animal studies [37], requires further investigation in the context of human populations.

Therefore, to accurately discern the determinants of seasonal asthma variations, particularly the observed gender-specific patterns, future research should incorporate statistical analyses that control for these identified confounders, including indoor air quality, viral infections, hormonal fluctuations, weather patterns, and access to healthcare.

One interesting data point is the spike in women’s asthma in October. While meteorological factors could potentially contribute to this pattern (beginning of the cold autumn season), the lack of a corresponding increase in male asthma (flatlined from September to November) cases suggests that socioeconomic factors may play a more significant role. The mothers tend to be the primary caregivers in our region, and after an extended summer break that lasts almost 3 months, children begin school in September, potentially transmitting customary respiratory infections from offspring to mothers until immunity is developed [38]. Moreover, given the circumstances of school starting and children catching respiratory infections, another underlying mechanism could be the supplementary stress of mothers [39] triggering asthma symptoms, but no studies have been conducted yet in our region.

Last, it is important to acknowledge that August and December might be outliers due to social factors like holiday celebrations, prevalent at the national level, leading to under-reporting of milder asthma cases. No specific studies indicate a decrease in the number of hospitalizations during the holidays, but some suggest patterns of reduced healthcare-seeking in this period [40].

A recent study conducted at a University Hospital in Timisoara, Romania, revealed that approximately 4.69% of the adult population in Timis County had been admitted for asthma-related issues over the last 11 years, suggesting potential regional variations in asthma incidence or differences in diagnostic and reporting practices within counties [41].

### 4.2. Age Groups and Comorbidities

Age-wise, the histogram (Figure 3a) presents a heavy head, correlating with the childhood-onset asthma phenotype [8]. A skewness value of −0.57 confirms that the age distribution leans slightly towards younger adults (18–35 years old), but a kurtosis of −0.04 is closer to a normal distribution, with the ages spread more evenly around the average.

The diffuse nature of the disease is apparent in its evolution (Figure 4). Initially, distinct community structures from the 18–49 age group (Figure 4a) become progressively integrated as age progresses (Figure 4b–d). This trend is evident in the reduced modularity and increased overlap between communities despite the use of a force-oriented layout to emphasize distinct clusters. The exact mathematical data (see Table from Section S1) confirm this. The observed peak in prevalence within the 60–69 age group suggests several potential underlying mechanisms: (1) chronic disease management—the 70+ group does not actively manage their health as well as the others due to cognitive decline or decreased healthcare utilization [42]; (2) survivorship bias—the age expectancy in Romania is 71.5 years for males and 79.3 years for females (2022), leading to a statistical exclusion of those age categories [43]. There are two main clusters in all age groups, but the asthma code (J45) shifts from the largest community in younger age groups (18–49, 50–59) to the secondary community in the last two groups. This suggests that the asthma management focus should shift with the patients’ age from the disease itself to addressing the underlying comorbidities [44]; some studies even proposed the term “geriatric asthma”, implying a comprehensive approach to this age group [45].

### 4.3. The Impact of Comorbidities

The asthma-containing community emerges as the most interconnected cluster of disorders across all age cohorts, as evidenced in Figure 5a, Figure 6a, Figure 7b and Figure 8b. This high degree of connectivity is a direct result of the underlying network construction. Throughout the different age groups, several recurring patterns become apparent, as outlined below:

- Asthma influence in the network as a whole, decreases with age, almost halving in the last age bracket compared to the first. This finding shows the importance of managing comorbidities in older patients and how complex geriatric pathology is, possibly explaining the challenges encountered in treating this age group. Recent literature supports this affirmation, confirming that a comprehensive, multidisciplinary approach is needed to better understand and treat the elderly [46].
- Respiratory failure (J96 code), a rational asthma comorbidity, presents an upward relative influence trend with age (21.89%, 24.93%, 25.01%, 32.29%), but the acute (J96.0) versus the chronic component (J96.1) have different patterns. Young patients are approximately 4.77 times more likely to experience acute respiratory failure (ARF) compared to chronic respiratory failure (CRF). Several studies show a higher prevalence of ARF in younger patients compared to older ones, with small changes in the diameter of the airways increasing the resistance and reducing airflow significantly, making smaller children more susceptible to this acute condition [47]. In contrast, older patients exhibit a smaller differential, with a roughly twofold increase in acute respiratory failure relative to chronic respiratory failure (2.21, 1.77, and 2.28, respectively). It is a known fact that as we age, our organ systems begin to decline in function slowly. These changes make older patients more susceptible to organ failure; therefore, CRF is found predominantly in this age group. Also, a longer amount of environmental exposure, such as household air pollution and occupational hazards, may influence this mechanism [48].
- Hypertension (I10) is always close to the J45 node, with a pervasive relative influence (8.35%, 37.07%, 52.24%, 56.43%), and a clear influence increase from the first group to the last. Corresponding to the recent literature, hypertension is a common comorbidity in asthma and other chronic conditions, and it shows a continuous rise from age 35 to 79 [49]. It is also associated with increased asthma severity, emphasizing the importance of managing hypertension as it may be a crucial factor in optimizing asthma care [50].
- Inadequate inhaler techniques or abuse of corticosteroid devices, leading to oral and, in the later stages, pulmonary candidiasis (B37.0, B37.1), have a slow upward trend (5.64%, 5.77%, 6.34%, 7.16%), making the elderly more susceptible to not using the medication correctly. However, this rather low prevalence may be biased due to physicians’ underreporting of this diagnosis in discharge summaries.
- Respiratory infections, as expected, play a triggering role in asthma pathology; however, their influence is minimal in the young age group (0.85%) and slightly higher in subsequent age cohorts (13.25%, 13.97%, 14.88%). This information is discordant with the literature on respiratory infections, particularly viral etiology, which play an important role in asthma exacerbation in younger patients [51]. A possible explanation might be that young adults in the first age group may experience milder infectious exacerbations, which do not require hospitalization compared to older patients.
- Surprisingly, allergies do not seem to have an important influence (0.53%, 1.61%, 0.56%, 0.29%). A potential reason for these findings is the lack of very young patients in our healthcare facility (children and adolescents), knowing that allergic asthma is more prevalent in this age group [52].
- Obesity shows a reverse U-shape trend, with the lowest percentage found in the younger group (1.91%), an upward trend for the 50–59 and 60–69 age groups (14.71%, 14.24%), and an abrupt decline in the elderly group 70+ (9%). Patients from these middle groups may have a higher percentage of obesity due to longer exposure to traditional cooking practices in our region (oil-fried meat, sauces, and processed food). A possible explanation for the decrease in the 70+ group may be the onset of frailty syndrome, a condition observed usually in the elderly, characterized by weight loss, sarcopenia, osteoporosis, and an altered state of nutrition, which can increase the risk of hospitalization and mortality [53].
- A downward trend is seen in smoking behavior (5.72%, 1.34%, 0.95%, 0.73%), with younger patients showing a tendency to exhibit a higher percentage of unhealthy habits like this. International statistics demonstrate similar results, stating that UK citizens over 65 have the lowest proportion of current smokers [54].

### 4.4. Asthma ICD-10 Complex Networks (Node Characteristic Tables in Age Groups of Asthma Patients)

This approach of complex networks helps find disease progression patterns and comorbidities. The results show an important shift in asthma comorbidities from the first age group (18–49) to the last (70+), suggesting the idea of shifting the therapeutic perspective from treating asthma per se to managing its comorbidities for better control in older patients.

In the youngest group (18–49 years old), the most pervasive role is played by lifestyle factors like long-term smoking behavior (5.72%), inadequate inhaler techniques, or abuse of corticosteroid devices (5.64%) and obesity due to excess calories (1.91%). Several connections were found in the literature, such as the ability of smoking to reduce asthma therapy effectiveness, leading to corticosteroid abuse, which further leads to weight gain and obesity [55]. Common respiratory infections (0.85%) and allergies (0.53%) are aggravating factors, but this small prevalence does not correlate with current data that suggest an important role of allergies and respiratory infections in asthma exacerbations, even in young patients [51]. The secondary cluster (Figure 5b) presents the accompanying factors that describe a patient with a long-term immunity depression associated with urinary infections (3.26%) and diverse anemia types (2.14%) onto which an opportunistic infection latches on: pneumonia(1.24%)/COVID-19– (0.44%), leading to a severe outcome like electrolytic imbalance, sepsis and cardiac arrest (2.63%). Several studies show a connection between anemia, especially iron deficiency anemia, and lower tract respiratory infections in young patients [56,57], but there is no clear connection between urinary infections and pneumonia, besides in bedridden patients with prolonged hospitalization [58]. The small communities in this age group (Figure 5c) are centered around morbidly obese patients with hepatic and lipoprotein metabolism dysfunctions, incipient diabetes mellitus type 2, as well as obstructive sleep apnea (2.23%). Cardiovascular disorders are present, but in a lower percentage (<0.08%).

The second age group (50–59 years old) reflects a more advanced disease stage through its clinical complexity. An integration of the first two communities in the young quartile can be seen in the first cluster here. This change emphasizes that as patients advance in age, they progressively accumulate comorbidities, being exposed for a longer period to risk factors such as smoking and obesity, which will subsequently decrease the body’s immunity, predisposing them to more severe infections like COVID-19 (0.54%) [59]. These factors subsequently create a predisposing environment for the emergence of cardiovascular diseases (13.44%), dyslipidemia, and their associated complications (1.62%) in the second community [60]. Also in this cluster, chronic lung diseases (14.17%) appear, predominantly represented by COPD and asthma-COPD overlap syndrome, which partially correlates with existing literature data, where the prevalence in this age group is 6.1% [61], but the prevalence differs from region to region. However, in Romania, the median age for COPD diagnosis is 56 years for males and 46 for females, with an overall prevalence of 9.7% for patients over 40 [62], suggesting that socioeconomic factors in our region may be implicated in this earlier age diagnosis. The last community contains neurological disorders, which are found at a lower prevalence (0.09%) but start to gradually increase between 64 and 80 years old [63].

The group of patients aged 60–69 years is associated with a community characterised by moderate cardiovascular, hepatic, and psychiatric disorders, linked with sepsis and death. Sepsis is correlated with the exacerbation of cardiovascular diseases and also the new onset of disease for survivors [64]. Some studies show that during sepsis, liver steatosis disease exhibits a higher incidence of acute kidney injury, with the need for renal replacement therapy and more frequent use of invasive mechanical ventilation. Additionally, some psychiatric disorders seem to be associated with cardiovascular diseases such as hypertension, ischemic heart disease, angina pectoris, and others [65]. Interestingly, a shift can be seen from the first community in the last group (50–59) to the second community in this group, which was an integration of the first two communities in the youngest age group (18–49). This supports the fact that as a person ages, they progressively accumulate comorbidities that are intricate with each other, ultimately weakening the body and making asthma more difficult to treat, placing it somewhere in second place alongside its comorbidities [66].

The first cluster of moderate cardiovascular diseases, neuronal disorders, sepsis, and exitus characterizes the last group of elderly patients (70+). This correlates with literature findings that rates for cardiovascular disorders in people over 60 are estimated to be around 60% [67], and for neurological disorders around 5–55%, depending on the specific condition [68]. The secondary cluster contains all the accompanying comorbidities mentioned above, with the second community having the most comorbidities. For example, a retrospective cohort study [69] revealed that the incidence of pleural empyema was 2.03 times higher in patients with asthma compared to those without it, with an adjusted hazard ratio of 2.12. The same study found that the risk of pleural empyema was greater in older individuals, indicating that age is a significant factor in the development of pleural complications in asthma patients

COVID-19 was present in three of the four groups of patients (50–59, 60–69, and 70+) in the first cluster, reflecting the increased influence of only 2 pandemic years in the overall population evolution, studied over 11 years.

### 4.5. Asthma Treatment and Management Implications

For clinicians, a comprehensive understanding of the complex interplay of comorbidities and their age-dependent variations is crucial for optimizing asthma management. This knowledge can inform personalized treatment strategies in the following ways:

- Prioritizing multidisciplinary care: Effective management of asthma in older adults often requires a coordinated approach involving pulmonologists, cardiologists, geriatricians, and other specialists.
- Minimizing the risk of drug interactions: Polypharmacy is common in older adults with multiple comorbidities, increasing the potential for adverse drug reactions.
- Improving long-term prognosis: By personalizing treatment and consistent prospective screening, for patients with co-existing conditions.

## 5. Conclusions

This study aimed to retrospectively investigate the asthma trends in our region. Several key findings emerged:

Admission Trends: While a rising trend was observed, current admission numbers remain below pre-pandemic levels. This discrepancy warrants further investigation to determine the underlying factors, such as changes in patient behavior or shifts in asthma epidemiology.

Gender Disparities: There is an observed increase in male admissions during 2020–2021, which may be linked to the higher susceptibility of males with asthma to severe COVID-19 infections, confirming the literature findings. Although females tend to have higher overall prevalence, as expected, their admission rates present a seasonal distribution, specific for the social factors in the region. Pinpointing the causation, not just the correlation, should be performed in collaboration with sociologists and psychologists, as these findings can be translated into modifiable lifestyle factors to improve life quality in asthmatic patients.

Age-Related Patterns: The age distribution of admissions exhibited a characteristic “heavy head”, consistent with the predominance of childhood-onset asthma. In the comorbidities network, the relative importance of asthma decreased with age, highlighting the geriatric asthma complexity, as well as the critical role of multidisciplinary managing comorbidities in older patients. As expected, respiratory failure emerged as a significant comorbidity, with an increasing relative influence with age; however, its pattern quantification is interesting, as younger patients tend to experience higher rates of acute respiratory failure during severe exacerbations, while chronic respiratory failure increased in older age groups. Confirming the age distribution, this offers specific numerical data in adult patients, while, to our knowledge, such studies were only conducted on pediatric patients [47]. Surprisingly, the influence of allergies on asthma admissions appeared minimal across all age groups, warranting further investigation. Identification of distinct, age-related, asthma hospitalizations patterns with comorbidity quantization is the beginning of a science-based, region-specific personalized approach to asthma management. Our methodology and the numerical results can serve as a valuable refinement to existing international guidelines, such as those provided by the Global Initiative for Asthma (GINA), by tailoring recommendations to the specific epidemiological and clinical characteristics of the local population.

From a clinical perspective, these findings underscore the need for a multifaceted approach to asthma management that considers age, gender, and the evolving interplay of comorbidities. A multidisciplinary approach is crucial for optimizing the care of patients with asthma, particularly in older adults, by addressing the unique challenges posed by co-existing conditions and age-related physiological changes.

The authors used ChatGPT-4-turbo, an AI-powered large language model developed by OpenAI, to improve the manuscript’s language and readability exclusively. All the scientific content, interpretations, and conclusions are based on the original work of the authors.

## Figures and Tables

**Table 1:** Baseline characteristics table.

Criteria	Asthma Cohort Variation
Age (years, mean ± SD)	59.6 ± 14.8
Female (%)	57.2
Length of Stay (Days, mean ± SD)	4.5 ± 5.9
Age Category 18–49 (%)	22.7
Age Category 50–59 (%)	21.4
Age Category 60–69 (%)	28.8
Age Category 70+ (%)	27.1
Year 2013 (%)	10.5
Year 2014 (%)	10
Year 2015 (%)	8.8
Year 2016 (%)	7.4
Year 2017 (%)	9.7
Year 2018 (%)	12.8
Year 2019 (%)	12
Year 2020 (%)	6.5
Year 2021 (%)	5
Year 2022 (%)	7.8
Year 2023 (%)	9.4

**Table 2:** Complex patterns of comorbidities and their variations with age. (Cs—corticosteroids, CVD—cardiovascular disorders, Adv CVD—advanced CVD, COPD—chronic obstructive pulmonary disorder, D—disorder, GERD—gastroesophageal reflux diseases).

	18–49 y	50–59 y	60–69 y	70 y +
**PURPLE ** **(main community)**	Smoking Cs abuse (Candidosis) Obesity Resp. Inf Lung Fibrosis Allergies	Obesity Resp. Infections Cs Abuse (Candidosis) Urinary D. Lung Fibrosis Sepsis and Death Anemia Psychiatric D. Allergies Smoking COVID-19	Moderate-impact CVD Hepatic D. Sepsis and Death Psychiatric D. COVID-19 GERD	Moderate-impact CVD Sepsis and death Neuronal D. COVID-19
**GREEN ** **(secondary community)**	Urinary Infection Sepsis and Death Anemia Resp Inf COVID-19	Chronic pulm.D. CVD. Obesity Complications Pleural D. Addiction D.	Adv CVD Obesity Resp Inf Urinary D. Cs Abuse (Candidosis) COPD Lung Fibrosis Anemia Pleural. D. Smoking Allergies Lung Neo Hydro/electro D.	Adv CVD Resp Inf Urinary D. Obesity Cs Abuse (Candidosis) COPD Anemia Lung Fibrosis Pleural D. Smoking Lung Neo Ictus Allergies Sepsis Psych D.
**BLUE ** **(tertiary community)**	Morbid Obesity Others	Neuronal D. Others	Cataracts Others	Pulmonary metastases Others

## Data Availability

The datasets generated during and/or analyzed during the current study are not publicly available due to privacy (GDPR) restrictions. However, anonymized datasets may be available upon reasonable request and with appropriate ethical approval. Requests to access the datasets should be directed to Dr. Oancea (oancea@umft.ro).

## Supplementary Materials

The following supporting information can be downloaded at https://www.mdpi.com/article/10.3390/jcm14072350/s1: Section S1: Complex networks details of age range asthma comorbidities; Section S2: Nodes Characteristics per Age Group.

## References

[1] GINA-2024-Strategy-Report-24_05_22_WMS.pdf [Internet]. https://ginasthma.org/wp-content/uploads/2024/05/GINA-2024-Strategy-Report-24_05_22_WMS.pdf.

[2] WHO Asthma [Internet]. https://www.who.int/news-room/fact-sheets/detail/asthma.

[3] Finland: EU Country with Highest Share of Asthmatics [Internet]. https://ec.europa.eu/eurostat/web/products-eurostat-news/-/edn-20210924-1.

[4] Pate C.A., Zahran H.S., Qin X., Johnson C., Hummelman E., Malilay J. (2021). Asthma Surveillance—United States, 2006–2018. MMWR Surveill. Summ..

[5] Mortimer K., Lesosky M., García-Marcos L., Asher M.I., Pearce N., Ellwood E., Bissell K., El Sony A., Ellwood P., Marks G.B. (2022). The burden of asthma, hay fever and eczema in adults in 17 countries: GAN Phase I study. Eur. Respir. J..

[6] GBD 2019 Diseases and Injuries Collaborators (2020). Global burden of 369 diseases and injuries in 204 countries and territories, 1990–2019: A systematic analysis for the Global Burden of Disease Study 2019. Lancet Lond Engl..

[7] Kankaanranta H., Kauppi P., Tuomisto L.E., Ilmarinen P. (2016). Emerging Comorbidities in Adult Asthma: Risks, Clinical Associations, and Mechanisms. Mediat. Inflamm..

[8] Ilmarinen P., Tuomisto L.E., Kankaanranta H. (2015). Phenotypes, Risk Factors, and Mechanisms of Adult-Onset Asthma. Mediat. Inflamm..

[9] Trivedi M., Denton E. (2019). Asthma in Children and Adults—What Are the Differences and What Can They Tell us About Asthma?. Front. Pediatr..

[10] Kong D.L., Qin Z., Shen H., Jin H.Y., Wang W., Wang Z.F. (2017). Association of Obstructive Sleep Apnea with Asthma: A Meta-Analysis. Sci. Rep..

[11] Ledford D.K., Lockey R.F. (2013). Asthma and comorbidities. Curr. Opin. Allergy Clin. Immunol..

[12] Akmatov M.K., Ermakova T., Holstiege J., Steffen A., von Stillfried D., Bätzing J. (2020). Comorbidity profile of patients with concurrent diagnoses of asthma and COPD in Germany. Sci. Rep..

[13] Zhang S.Q., Xiong X.F., Wu Z.H., Huang T.T., Cheng D.Y. (2021). Clinical features of asthma with comorbid bronchiectasis. Medicine.

[14] Cazzola M., Rogliani P., Ora J., Calzetta L., Matera M.G. (2022). Asthma and comorbidities: Recent advances. Pol. Arch. Intern. Med..

[15] Pollevick M.E., Xu K.Y., Mhango G., Federmann E.G., Vedanthan R., Busse P., Holguin F., Federman A.D., Wisnivesky J.P. (2021). The Relationship Between Asthma and Cardiovascular Disease. Chest.

[16] Forte G.C., Grutcki D.M., Menegotto S.M., Pereira R.P., de Tarso Roth Dalcin P. (2013). Prevalence of obesity in asthma and its relations with asthma severity and control. Rev. Assoc. Médica. Bras..

[17] Kauppi P., Linna M., Jantunen J., Martikainen J.E., Haahtela T., Pelkonen A., Mäkelä M. (2015). Chronic Comorbidities Contribute to the Burden and Costs of Persistent Asthma. Mediat. Inflamm..

[18] Cunha M.S., Amaral R., Pereira A.M., Almeida R., Alves-Correia M., Loureiro C.C., Lopes C., Carvalho J., Ribeiro C., Vidal C. (2023). Symptoms of anxiety and depression in patients with persistent asthma: A cross-sectional analysis of the INSPIRERS studies. BMJ Open.

[19] Baan E.J., De Roos E.W., Engelkes M., De Ridder M., Pedersen L., Berencsi K., Prieto-Alhambra D., Lapi F., Van Dyke M.K., Rijnbeek P. (2022). Characterization of Asthma by Age of Onset: A Multi-Database Cohort Study. J. Allergy Clin. Immunol. Pract..

[20] Mendy A., Mersha T.B. (2022). Comorbidities in childhood-onset and adult-onset asthma. Ann. Allergy Asthma. Immunol..

[21] Perry T.T., Grant T.L., Dantzer J.A., Udemgba C., Jefferson A.A. (2024). Impact of socioeconomic factors on allergic diseases. J. Allergy Clin. Immunol..

[22] Sinharoy A., Mitra S., Mondal P. (2018). Socioeconomic and Environmental Predictors of Asthma-Related Mortality. J. Environ. Public. Health..

[23] Grant T., Croce E., Matsui E.C. (2022). Asthma and the social determinants of health. Ann. Allergy Asthma. Immunol..

[24] Szefler S.J., Raphiou I., Zeiger R.S., Stempel D., Kral K., Pascoe S. (2019). Seasonal variation in asthma exacerbations in the AUSTRI and VESTRI studies. ERJ. Open Res..

[25] Dounce-Cuevas C.A., Flores-Flores A., Bazán M.S., Portales-Rivera V., Morelos-Ulíbarri A.A., Bazán-Perkins B. (2023). Asthma and COVID-19: A controversial relationship. Virol. J..

[26] Chen Y.L., Lin Y.Y., Chin P.W., Chen C.C., Cheng C.G., Cheng C.A. (2024). Analyzing COVID-19 and Air Pollution Effects on Pediatric Asthma Emergency Room Visits in Taiwan. Toxics.

[27] Karcher H., Schoenberger M., Rayban T., Kelly C., Heaney A., Mackay A. (2023). Impact of COVID-19 measures on exacerbation rates and healthcare visits in US asthma patients. Allergy Asthma. Proc..

[28] Sykes D.L., Faruqi S., Holdsworth L., Crooks M.G. (2021). Impact of COVID-19 on COPD and asthma admissions, and the pandemic from a patient’s perspective. ERJ. Open Res..

[29] Imlach F., McKinlay E., Kennedy J., Pledger M., Middleton L., Cumming J., McBride-Henry K. (2021). Seeking Healthcare During Lockdown: Challenges, Opportunities and Lessons for the Future. Int. J. Health Policy Manag..

[30] Lee S.C., Son K.J., Han C.H., Jung J.Y., Park S.C. (2020). Impact of comorbid asthma on severity of coronavirus disease (COVID-19). Sci. Rep..

[31] Caminati M., Vultaggio A., Matucci A., Senna G., Almerigogna F., Bagnasco D., Chieco-Bianchi F., Cosini F., Girelli D., Guarnieri G. (2021). Asthma in a large COVID-19 cohort: Prevalence, features, and determinants of COVID-19 disease severity. Respir. Med..

[32] Khalid M., Almasri T., Goble S., Johnson D., Gilbertson D., Linzer M., Strykowski R. (2024). Seasonal variations and social disparities in asthma hospitalizations and outcomes. J. Asthma. Off J. Assoc. Care Asthma..

[33] utilizarea_timpului_r13.pdf [Internet]. https://insse.ro/cms/files/statistici/comunicate/com_anuale/util_timpul/utilizarea_timpului_r13.pdf.

[34] Sood A., Assad N.A., Barnes P.J., Churg A., Gordon S.B., Harrod K.S., Irshad H., Kurmi O.P., Martin W.J., Meek P. (2018). ERS/ATS workshop report on respiratory health effects of household air pollution. Eur. Respir. J..

[35] Dai X., Bui D.S., Perret J.L., Lowe A.J., Frith P.A., Bowatte G., Thomas P.S., Giles G.G., Hamilton G.S., Tsimiklis H. (2021). Exposure to household air pollution over 10 years is related to asthma and lung function decline. Eur. Respir. J..

[36] He L., Evans S., Norris C., Barkjohn K., Cui X., Li Z., Zhou X., Li F., Zhang Y., Black M. (2023). Associations between personal apparent temperature exposures and asthma symptoms in children with asthma. PLoS ONE.

[37] Fuseini H., Newcomb D.C. (2017). Mechanisms driving gender differences in asthma. Curr. Allergy Asthma. Rep..

[38] MacIntyre C.R., Ridda I., Seale H., Gao Z., Ratnamohan V.M., Donovan L., Zeng F., Dwyer D.E. (2012). Respiratory viruses transmission from children to adults within a household. Vaccine.

[39] Yang F., Zhou J., Xiao H., Wu X., Cui Y., Huang H., Zheng S., Li H. (2024). Caregiver burden among parents of school-age children with asthma: A cross-sectional study. Front. Public. Health.

[40] Briciu V., Topan A., Calin M., Dobrota R., Leucuta D.C., Lupse M. (2023). Comparison of COVID-19 Severity in Vaccinated and Unvaccinated Patients during the Delta and Omicron Wave of the Pandemic in a Romanian Tertiary Infectious Diseases Hospital. Healthcare.

[41] Trusculescu A.A., Ancusa V.M., Pescaru C.C., Wellmann N., Fira-Mladinescu C., Oancea C.I., Fira-Mladinescu O. (2024). A Multifaceted Exploration of Status Asthmaticus: A Retrospective Analysis in a Romanian Hospital. J. Clin. Med..

[42] Hong S.N., Lai F.T.T., Wang B., Choi E.P.H., Wong I.C.K., Lam C.L.K., Wan E.Y.F. (2024). Age-specific Multimorbidity Patterns and Burden on All-Cause Mortality and Public Direct Medical Expenditure: A Retrospective Cohort Study. J. Epidemiol. Glob. Health.

[43] State of Health in the EU, Romania.pdf [Internet]. https://health.ec.europa.eu/system/files/2023-12/2023_chp_ro_english.pdf.

[44] Khosa J.K., Louie S., Moreno P.L., Abramov D., Rogstad D.K., Alismail A., Matus M.J., Tan L.D. (2023). Asthma Care in the Elderly: Practical Guidance and Challenges for Clinical Management—A Framework of 5 “Ps”. J. Asthma. Allergy..

[45] Battaglia S., Benfante A., Spatafora M., Scichilone N. (2016). Asthma in the elderly: A different disease?. Breathe.

[46] Warm K., Hedman L., Stridsman C., Lindberg A., Rönmark E., Backman H. (2023). Age-related differences in associations between uncontrolled asthma, comorbidities and biomarkers in adult-onset asthma. J Asthma..

[47] Han P., Jiao A., Yin J., Zou H., Liu Y., Li Z., Wang Q., Wu J., Shen K. (2024). Analysis of risk factors for acute attacks complicated by respiratory failure in children with asthma. Front. Pediatr..

[48] Joshi P.R. (2024). Pulmonary Diseases in Older Patients: Understanding and Addressing the Challenges. Geriatrics.

[49] Cheng W., Du Y., Zhang Q., Wang X., He C., He J., Jing F., Ren H., Guo M., Tian J. (2022). Age-related changes in the risk of high blood pressure. Front. Cardiovasc. Med..

[50] Zolotareva O., Saik O.V., Königs C., Bragina E.Y., Goncharova I.A., Freidin M.B., Dosenko V.E., Ivanisenko V.A., Hofestädt R. (2019). Comorbidity of asthma and hypertension may be mediated by shared genetic dysregulation and drug side effects. Sci. Rep..

[51] Mthembu N., Ikwegbue P., Brombacher F., Hadebe S. (2021). Respiratory Viral and Bacterial Factors That Influence Early Childhood Asthma. Front. Allergy.

[52] Schiffers C., Wouters E.F., Breyer-Kohansal R., Buhl R., Pohl W., Irvin C.G., Breyer M.-K., Hartl S. (2023). Asthma Prevalence and Phenotyping in the General Population: The LEAD (Lung, hEart, sociAl, boDy) Study. J. Asthma. Allergy..

[53] Greco E.A., Pietschmann P., Migliaccio S. (2019). Osteoporosis and Sarcopenia Increase Frailty Syndrome in the Elderly. Front. Endocrinol..

[54] Adult Smoking Habits in the UK—Office for National Statistics [Internet]. https://www.ons.gov.uk/peoplepopulationandcommunity/healthandsocialcare/healthandlifeexpectancies/bulletins/adultsmokinghabitsingreatbritain/2022.

[55] Gaffin J.M., Castro M., Bacharier L.B., Fuhlbrigge A.L. (2022). The Role of Comorbidities in Difficult-to-Control Asthma in Adults and Children. J. Allergy Clin. Immunol. Pract..

[56] STEPAND, DOPD, MOROŞANUA, VINTILESCUB, NICULESCUC (2018). Implications of the Iron Deficiency in Lower Tract Respiratory Acute Infections in Toddlers. Curr. Health Sci. J..

[57] Jayamanna U., Jayaweera J.A.A.S. (2023). Childhood Anemia and Risk for Acute Respiratory Infection, Gastroenteritis, and Urinary Tract Infection: A Systematic Review. J. Pediatr. Infect. Dis..

[58] Matsusaka K., Kawakami G., Kamekawa H., Momma H., Nagatomi R., Itoh J., Yamaya M. (2018). Pneumonia risks in bedridden patients receiving oral care and their screening tool: Malnutrition and urinary tract infection-induced inflammation. Geriatr. Gerontol. Int..

[59] de Frel D.L., Atsma D.E., Pijl H., Seidell J.C., Leenen P.J.M., Dik W.A., van Rossum E.F.C. (2020). The Impact of Obesity and Lifestyle on the Immune System and Susceptibility to Infections Such as COVID-19. Front. Nutr..

[60] Frasca D., Blomberg B.B., Paganelli R. (2017). Aging, Obesity, and Inflammatory Age-Related Diseases. Front. Immunol..

[61] Yan X., Xu L., Shi B., Wang H., Xu X., Xu G. (2020). Epidemiology and risk factors of chronic obstructive pulmonary disease in Suzhou: A population-based cross-sectional study. J. Thorac. Dis..

[62] Mihaltan F.D., Furtunescu F., Nemes R.M., Farcasanu D., Daramus I.M. (2014). COPD prevalence in Romania and possible influence of social and household characteristics. Eur. Respir. J..

[63] Feigin V.L., Vos T., Nichols E., O Owolabi M., Carroll W.M., Dichgans M., Deuschl G., Parmar P., Brainin M., Murray C. (2020). The global burden of neurological disorders: Translating evidence into policy. Lancet Neurol..

[64] Mankowski R.T., Yende S., Angus D.C. (2019). Long-Term Impact of Sepsis on Cardiovascular Health. Intensive Care Med..

[65] Shen Q., Mikkelsen D.H., Luitva L.B., Song H., Kasela S., Aspelund T., Bergstedt J., Lu Y., Sullivan P.F., Ye W. (2023). Psychiatric disorders and subsequent risk of cardiovascular disease: A longitudinal matched cohort study across three countries. eClinicalMedicine.

[66] Melani A.S. (2013). Management of asthma in the elderly patient. Clin. Interv. Aging..

[67] Gadó K., Szabo A., Markovics D., Virág A. (2022). Most common cardiovascular diseases of the elderly—A review article. Dev. Health Sci..

[68] Stancu P., Hentsch L., Seeck M., Zekry D., Graf C., Fleury V., Assal F. (2024). Neurology of Aging: Adapting Neurology Provision for an Aging Population. Neurodegener. Dis..

[69] Liao W.C., Lin C.L., Shen T.C., Tu C.Y., Hsia T.C., Hsu W.H. (2022). Risk of Pleural Empyema in Adult Patients With Asthma: A Nationwide Retrospective Cohort Study. Front. Med..

