# Supplementary material for: Age-Related Variations and Seasonal Influences: A Network Analysis of Comorbidities in Asthma Hospitalizations (2013–2023)
Source: J Clin Med. 2025 Mar 29;14(7):2350. doi: 10.3390/jcm14072350 (PMC11989303; doi:10.3390/jcm14072350)
Supplement: Supplementary file 1 [file jcm-14-02350-s001.zip › jcm-3494012-supplementary.pdf]

Section S1. Complex networks details of age range asthma comorbidities

Table S1. Complex network characteristics of age range asthma comorbidities.

Age Group	18-49	50-59	60-69	70+
Nodes	956	996	1266	1288
Edges	35720	54459	98880	125157
Average Degree	28.611	37.707	47.605	55.896
Average Weighted Degree	74.728	109.355	152.209	194.343
Diameter	2	2	3	3
Radius	1	1	2	2
Average Path length	1.97	1.962	1.964	1.957
Graph Density	0.03	0.038	0.038	0.043
Connected components	1	1	1	1
Modularity	0.112	0.089	0.081	0.092
Average Clustering Coefficient:	0.803	0.804	0.816	0.813
Total triangles	118474	218918	484938	681216
Communities	12	14	13	9
Resolution for modularity	1	1	1	1
Communities' percentages	47.59%, 38.08%, 8.68%, 3.24%, 0.52%, 0.52%, 0.31%, 0.21%, 0.21%, 0.21%, 0.21%, 0.21%,	60.14%, 33.23%, 1.81%, 0.9%, 0.7%, 0.5%, 0.5%, 0.4%, 0.4%, 0.4%, 0.3%, 0.3%, 0.2%, 0.2%	54.98%, 41.79%, 1.03%, 0.32%, 0.32%, 0.24%, 0.24%, 0.24%, 0.24%, 0.16%, 0.16%, 0.16%, 0.16%	51.55%, 44.33%, 1.32%, 1.16%, 0.54%, 0.39%, 0.31%, 0.23%, 0.16%

Section S2. Nodes Characteristics per Age Group

Group 18-49

Label	Degree	Weighted Degree	Betweeness centrality	Modularity class
A04.7	48	66	0.000279	0
A04.9	41	76	0.0002	0
A05.9	12	13	0.000008	0
A07.1	31	41	0.000125	0
A08.0	4	4	0	0
A08.3	5	5	0	0
A09	58	90	0.000521	0
A15.0	90	177	0.000727	0
A15.1	27	34	0.000027	0
A15.3	32	38	0.00003	1
A16.0	49	81	0.000224	1
A16.1	6	6	0	0

A16.2	16	22	0.000019	1
A16.3	2	2	0	0
A16.5	9	11	0.000001	1
A18.2	31	33	0.000013	0
A26.0	7	7	0	1
A38	3	3	0	1
A40.3	7	7	0	0
A41.1	9	9	0	0
A41.8	54	86	0.000172	0
A41.9	152	351	0.003386	0
A46	5	5	0	0
A49.0	21	24	0.000055	9
A49.1	6	6	0	1
A49.2	10	10	0	9
A51.5	10	10	0	9
A52.8	7	7	0	1
A53.9	12	12	0	1
A69.2	3	3	0	4
A74.9	8	8	0	0
B00.9	4	4	0	1
B01.8	7	8	0	0
B01.9	9	10	0.000001	0
B05.2	5	5	0	0
B05.9	5	5	0	0
B07	7	7	0	0
B08.1	9	9	0	0
B15.9	5	6	0	9
B18.0	20	22	0.000028	4
B18.1	79	139	0.000682	0
B18.2	36	54	0.000061	0
B18.9	18	23	0.000014	4
B20	64	109	0.000245	1
B22	19	23	0.000004	4
B23.0	4	4	0	1
B23.8	36	51	0.000151	0
B27.0	7	7	0	0
B30.8	5	5	0	1
B34.0	6	6	0	0
B34.2	23	41	0.00002	0
B37.0	344	1619	0.026209	1
B37.1	87	179	0.0006	1
B37.3+	6	6	0	0
B37.7	59	109	0.00027	1
B37.88	18	27	0.000015	0
B37.9	15	20	0.000016	0
B44.1	9	10	0.000003	1

B47.9	11	11	0	1
B59+	9	9	0	0
B67.0	7	7	0	0
B67.1	7	7	0	0
B67.3	8	14	0	0
B85.2	12	12	0	1
B90.0	17	17	0	1
B90.9	211	857	0.006424	1
B91	8	8	0	0
B95.3	6	6	0	1
B95.5	8	8	0	1
B95.6	54	85	0.000225	4
B96.1	31	46	0.000022	1
B96.2	95	155	0.000613	0
B96.5	44	69	0.000198	1
B96.81	16	17	0.000003	0
B96.88	137	398	0.002251	1
B97.0	5	5	0	3
B97.2	126	504	0.001914	0
B99	30	47	0.000102	0
C16.9	13	13	0	0
C18.9	13	13	0	0
C34.0	3	3	0	0
C34.1	15	23	0.000007	1
C34.3	1	1	0	1
C34.8	15	16	0.000008	1
C34.9	42	57	0.000141	0
C40.2	7	7	0	11
C41.2	7	7	0	11
C44.4	5	5	0	1
C49.9	10	10	0	0
C50.9	13	14	0.000012	0
C53.9	9	10	0	5
C55	8	8	0	0
C71.5	8	8	0	1
C78.0	7	7	0	3
C78.1	7	7	0	3
C79.5	6	6	0	0
C81.1	7	7	0	1
C81.9	16	27	0.000006	0
D04.4	3	3	0	0
D14.1	9	9	0	0
D14.3	7	10	0.000001	1
D15.2	4	4	0	4
D17.1	6	6	0	2
D18.02	8	8	0	0

D18.08	16	18	0.000009	1
D24	10	20	0	1
D25.9	17	20	0.000014	1
D26.1	13	14	0.000005	0
D27	2	2	0	0
D32.0	9	9	0	9
D35.0	6	6	0	8
D35.2	7	7	0	0
D38.0	5	5	0	0
D38.1	39	52	0.000118	0
D38.3	13	21	0.000007	0
D38.6	6	6	0	0
D40.0	27	27	0	0
D41.1	8	8	0	0
D46.4	10	14	0.000002	0
D47.3	31	38	0.000046	4
D50.0	4	4	0	1
D50.8	55	94	0.000191	0
D50.9	32	40	0.000042	1
D53.0	14	16	0.000006	0
D53.8	27	32	0.000019	1
D53.9	201	621	0.006233	0
D56.1	6	6	0	0
D58.2	40	134	0.000101	1
D62	7	7	0	1
D63.0*	10	11	0.000004	1
D63.8*	41	69	0.000119	0
D64.3	5	5	0	4
D64.8	58	100	0.000152	1
D64.9	183	409	0.004637	0
D68.8	16	28	0.000005	0
D68.9	5	5	0	1
D69.0	11	13	0.000002	0
D69.5	40	55	0.00012	0
D69.6	84	142	0.000689	0
D72.0	6	6	0	0
D72.1	70	125	0.00052	1
D72.8	116	331	0.001386	1
D72.9	28	34	0.000056	0
D73.0	7	7	0	0
D73.9	10	10	0	1
D75.0	25	67	0.000019	1
D75.1	57	172	0.000164	1
D75.2	69	108	0.000209	0
D75.9	15	16	0.000007	0
D76.1	7	7	0	1

D81.9	8	9	0.000001	0
D86.0	33	46	0.000038	0
D86.1	5	5	0	1
D86.2	13	17	0.000006	1
E03.2	2	2	0	1
E03.8	19	28	0.000009	4
E03.9	39	64	0.000072	0
E04.1	15	17	0.000019	0
E05.0	12	14	0.000003	0
E05.9	16	25	0.000002	1
E06.2	12	12	0	4
E06.3	67	112	0.000473	0
E06.9	22	25	0.000012	0
E07.8	19	23	0.000011	0
E09.01	11	14	0.000004	1
E09.9	37	60	0.000142	4
E10.11	13	13	0	1
E10.29	10	10	0	0
E10.31	6	6	0	1
E10.9	30	35	0.00002	1
E11.42	13	13	0	1
E11.65	12	12	0	0
E11.69	12	12	0	1
E11.72	4	4	0	1
E11.8	49	110	0.000117	1
E11.9	142	472	0.002103	4
E13.01	5	5	0	0
E13.9	8	9	0	0
E14.9	9	9	0	0
E16.2	8	8	0	9
E16.3	13	13	0	1
E16.8	18	22	0.000008	9
E16.9	6	7	0.000001	0
E23.0	5	5	0	1
E23.2	5	5	0	1
E27.0	7	7	0	0
E34.3	3	3	0	10
E44.1	44	64	0.000095	1
E46	29	34	0.000028	0
E63.8	8	8	0	1
E64.0	5	5	0	3
E66.0	255	1147	0.010091	1
E66.1	6	7	0	4
E66.2	31	56	0.00005	1
E66.8	25	38	0.000027	4
E66.9	157	535	0.0026	4

E72.1	8	8	0	1
E75.6	4	4	0	1
E77.9	8	8	0	0
E78.0	136	280	0.002877	4
E78.1	19	22	0.000015	0
E78.2	48	83	0.000124	4
E78.4	18	21	0.000011	4
E78.5	58	119	0.000168	1
E78.8	27	27	0	0
E78.9	10	12	0	0
E79.0	157	341	0.002702	1
E83.5	18	21	0.000039	0
E84.0	16	20	0.000008	0
E84.9	18	22	0.000036	1
E85.0	11	15	0.000003	1
E85.4	11	13	0.000003	1
E85.9	20	21	0.000023	4
E86	151	271	0.00385	0
E87.0	48	68	0.000076	0
E87.1	106	255	0.001037	0
E87.2	95	193	0.000632	0
E87.3	17	19	0.000001	0
E87.5	45	64	0.000067	0
E87.6	130	304	0.001636	0
E87.7	4	4	0	1
E87.8	131	335	0.002996	0
E88.2	13	13	0	1
E88.8	6	6	0	1
E88.9	47	82	0.000067	1
E89.0	7	8	0.000004	1
F06.2	9	9	0	0
F06.3	6	6	0	1
F06.6	124	370	0.001904	0
F06.9	7	7	0	0
F07.1	5	5	0	1
F07.2	12	12	0	1
F10.2	34	45	0.000049	1
F10.6	8	8	0	1
F17.1	7	7	0	0
F17.2	134	318	0.002291	1
F17.3	8	8	0	1
F20.0	10	11	0.000001	0
F20.9	12	14	0.000005	0
F23.3	6	9	0	1
F29	8	8	0	0
F31.3	16	21	0.000015	1

F31.5	10	10	0	0
F31.8	15	17	0.000003	0
F31.9	19	24	0.000024	1
F32.00	9	9	0	5
F33.0	5	5	0	1
F33.8	5	6	0.000001	1
F33.9	90	158	0.000706	0
F41.0	47	57	0.000248	0
F41.1	29	31	0.000026	0
F41.2	112	264	0.00164	1
F41.3	10	10	0	0
F41.9	141	407	0.002125	1
F42.9	8	8	0	0
F45.0	10	10	0	9
F45.4	6	6	0	1
F45.9	7	7	0	0
F48.0	21	32	0.000031	0
F70.0	8	8	0	4
F70.9	34	41	0.000048	1
F71.0	5	5	0	1
F71.1	10	11	0.000012	1
F71.9	38	69	0.0001	1
F79.0	7	7	0	1
F92.0	20	22	0.00006	1
F99	10	12	0.000006	1
G06.0	5	5	0	1
G09	5	5	0	1
G21.0	5	5	0	1
G21.9	5	5	0	1
G25.9	18	19	0.000013	1
G31.0	27	27	0	0
G31.9	9	12	0	0
G40.41	5	5	0	1
G40.90	28	36	0.000058	0
G40.91	17	19	0.000006	0
G43.0	9	9	0	0
G43.9	8	9	0	1
G44.0	16	16	0	1
G44.1	22	34	0.000001	1
G47.30	102	299	0.000883	4
G47.31	27	42	0.00001	1
G47.32	119	424	0.001252	1
G47.39	13	13	0	1
G50.0	9	9	0	9
G50.9	5	5	0	0
G51.8	9	9	0	1

G51.9	9	9	0	5
G52.3	9	9	0	1
G54.1	7	7	0	0
G54.8	26	50	0.000005	1
G56.0	19	20	0.000026	0
G80.02	9	9	0	9
G80.9	9	12	0	0
G81.9	18	23	0.000011	1
G82.04	8	8	0	0
G82.10	5	5	0	0
G82.20	28	28	0	0
G82.40	9	9	0	0
G92	17	17	0	1
G95.9	8	8	0	0
H10.1	13	16	0.000003	0
H10.4	5	5	0	1
H10.9	7	8	0.000002	1
H19.2*	6	6	0	4
H25.9	8	8	0	1
H35.0	5	5	0	1
H35.5	6	6	0	1
H35.8	14	65	0.000001	4
H40.1	15	17	0.000005	1
H40.9	50	70	0.000136	0
H44.2	4	4	0	1
H50.9	31	32	0.000015	0
H51.8	27	27	0	0
H54.0	16	17	0.000005	1
H54.2	7	7	0	0
H54.4	3	3	0	7
H57.8	5	5	0	1
H65.9	4	4	0	1
H80.9	10	10	0	1
H81.4	10	10	0	4
H81.9	23	36	0.000002	1
H90.2	5	5	0	0
H90.3	14	17	0.000005	1
H90.8	5	5	0	1
H91.3	6	6	0	1
H91.9	9	10	0.000001	1
H93.8	8	8	0	1
I05.1	21	24	0.000004	1
I05.2	6	6	0	1
I05.9	5	5	0	1
I06.1	5	5	0	1
I07.0	21	53	0.000007	1

I07.1	74	155	0.000359	1
I08.0	13	13	0	1
I08.1	10	10	0	1
I09.1	10	10	0	0
I10	438	2627	0.043839	1
I11.0	65	125	0.000246	1
I11.9	16	28	0.000006	1
I15.2	5	5	0	1
I15.9	10	10	0	0
I20.8	29	35	0.000037	4
I20.9	45	80	0.000084	1
I21.9	14	16	0.000008	0
I25.0	16	18	0.000009	4
I25.2	48	124	0.000111	4
I25.5	38	53	0.000124	1
I25.6	22	26	0.00001	1
I25.8	20	23	0.000013	4
I25.9	148	465	0.00222	1
I26.9	39	73	0.000046	1
I27.0	158	904	0.003019	1
I27.2	41	94	0.000073	1
I27.8	23	35	0.000017	1
I27.9	200	950	0.00457	1
I30.9	26	32	0.000027	0
I31.8	17	19	0.000007	4
I31.9	23	24	0.00002	1
I33.0	18	18	0	1
I34.0	127	398	0.001463	1
I34.1	27	30	0.000014	1
I34.2	8	8	0	1
I34.8	8	8	0	1
I35.0	13	14	0.000009	4
I35.1	45	112	0.000107	1
I35.9	5	5	0	1
I36.0	34	50	0.000047	1
I36.1	85	316	0.000557	1
I36.9	5	5	0	1
I37.1	35	48	0.000043	1
I40.0	8	8	0	1
I42.1	10	10	0	4
I42.2	16	17	0.000005	0
I44.4	6	6	0	0
I44.5	2	2	0	6
I44.6	10	10	0	0
I44.7	22	29	0.00004	1
I45.0	101	234	0.000895	1

I45.1	20	34	0.000011	1
I46.0	20	24	0.000006	1
I46.9	57	109	0.00026	0
I47.0	16	16	0	1
I47.1	61	142	0.00028	1
I47.2	25	38	0.000027	1
I47.9	36	66	0.000083	1
I48	53	67	0.00017	0
I49.1	22	37	0.000025	1
I49.3	58	125	0.000158	1
I49.4	14	17	0.000009	1
I49.5	3	3	0	1
I49.8	13	15	0.000017	1
I50.0	190	1065	0.004423	1
I50.9	67	151	0.000404	4
I51.7	12	14	0.000004	0
I51.9	6	6	0	1
I61.5	8	8	0	1
I63.8	27	27	0	0
I64	17	22	0.000006	4
I65.2	8	8	0	1
I67.0	13	13	0	1
I67.9	7	7	0	1
I69.3	15	15	0	0
I69.4	17	19	0.000004	1
I70.0	26	27	0.000033	0
I71.2	11	11	0	1
I73.0	5	5	0	1
I80.0	5	5	0	0
I80.8	4	4	0	0
I82.2	7	7	0	0
I83.1	13	13	0	1
I83.9	10	10	0	4
I84.2	11	11	0	0
I84.5	10	10	0	0
I84.9	15	19	0	1
I86.8	4	4	0	1
I87.0	28	34	0.000012	1
I87.1	8	8	0	0
I87.2	41	59	0.000113	0
I88.9	22	24	0.00001	0
I89.0	16	16	0	1
I95.9	13	16	0.000002	1
I97.9	18	19	0.000008	1
J00	6	6	0	1
J01.0	33	53	0.000123	1

J01.1	3	4	0	1
J01.4	3	3	0	0
J01.8	9	10	0	4
J01.9	8	9	0.000001	1
J02.0	6	6	0	4
J02.9	31	45	0.000121	0
J03.9	29	42	0.00003	0
J04.0	19	22	0.000004	1
J04.2	1	1	0	1
J06.0	6	6	0	0
J06.9	19	24	0.000009	4
J10.0	8	11	0	0
J10.1	16	22	0.000007	0
J10.8	7	7	0	0
J11.0	6	9	0.000001	1
J11.1	6	6	0	0
J12.0	3	3	0	0
J12.8	56	122	0.000258	0
J12.9	147	478	0.0033	1
J13	17	23	0.000003	1
J15.0	18	25	0.000015	1
J15.1	42	75	0.000109	1
J15.2	26	35	0.000015	1
J15.4	34	59	0.000077	1
J15.5	44	65	0.000072	1
J15.6	61	112	0.00034	0
J15.8	281	1241	0.016488	1
J15.9	161	514	0.003891	1
J16.8	5	5	0	0
J17.0*	5	5	0	0
J18.0	210	650	0.006489	0
J18.1	32	42	0.000078	0
J18.8	109	279	0.001438	1
J18.9	275	1156	0.014659	1
J20.0	3	3	0	1
J20.6	6	6	0	1
J20.9	20	26	0.000048	0
J21.8	7	7	0	0
J21.9	15	20	0.000009	1
J22	6	7	0	1
J30.0	18	22	0.000016	9
J30.1	109	296	0.000993	1
J30.2	5	6	0	1
J30.3	59	103	0.000381	1
J30.4	134	432	0.002791	1
J31.0	153	572	0.003377	1

J31.1	9	9	0	0
J32.0	58	93	0.000191	1
J32.1	4	4	0	1
J32.2	9	9	0	1
J32.3	16	28	0.000007	1
J32.8	21	25	0.00006	9
J32.9	38	55	0.000101	1
J33.0	16	30	0.000002	1
J33.1	23	28	0.000004	0
J33.8	15	18	0.000005	0
J33.9	41	60	0.000242	1
J34.2	98	211	0.000833	1
J34.8	23	27	0.000046	9
J35.0	22	27	0.000024	1
J35.9	9	9	0	1
J36	15	18	0.000008	0
J37.0	51	101	0.000087	1
J39.1	6	6	0	0
J39.8	6	6	0	0
J39.9	16	18	0.000002	1
J40	8	8	0	0
J41.0	61	141	0.000425	1
J41.1	3	3	0	4
J41.8	27	47	0.000014	1
J42	11	16	0.000002	1
J43.0	5	5	0	1
J43.1	22	45	0.000017	1
J43.2	5	5	0	1
J43.8	23	34	0.00002	0
J43.9	94	269	0.000941	1
J44.0	220	953	0.006932	1
J44.1	126	410	0.001732	1
J44.8	52	87	0.00022	1
J44.9	108	207	0.001378	0
J45	957	8865	0.524767	1
J46	46	91	0.000153	1
J47	299	1237	0.017433	1
J67.9	32	48	0.00003	0
J68.0	6	6	0	4
J80	22	24	0.000011	0
J81	12	12	0	0
J84.0	13	15	0.000002	0
J84.1	176	528	0.00435	1
J84.8	23	40	0.000045	1
J84.9	169	544	0.003978	1
J85.0	4	4	0	1

J85.1	13	17	0.000009	1
J85.2	5	5	0	0
J86.0	8	8	0	4
J86.9	10	10	0	1
J90	87	190	0.000587	1
J91*	17	21	0.000014	1
J92.0	36	65	0.000056	1
J92.9	116	319	0.001017	1
J93.0	4	4	0	0
J93.1	21	37	0.000003	1
J93.8	7	7	0	0
J93.9	16	17	0.000005	1
J94.1	48	87	0.000118	1
J94.8	44	66	0.000108	1
J95.3	7	7	0	1
J95.8	13	15	0.000002	0
J95.9	4	4	0	1
J96.0	570	3597	0.094978	1
J96.1	328	1743	0.019919	1
J98.1	9	13	0.000001	0
J98.3	39	66	0.000072	1
J98.4	6	6	0	1
J98.5	11	12	0.000002	0
J98.6	9	9	0	1
J98.8	19	40	0.000009	4
J98.9	9	9	0	1
J99.8*	14	14	0	0
K12.1	10	10	0	4
K20	6	6	0	0
K21.0	26	40	0.000069	4
K21.9	61	101	0.000246	0
K25.2	8	8	0	1
K25.7	7	7	0	1
K25.9	34	46	0.000035	1
K26.0	7	7	0	11
K26.7	32	47	0.000058	1
K26.9	29	38	0.000039	1
K27.0	7	7	0	11
K27.7	5	5	0	1
K27.9	16	18	0.000001	1
K28.9	7	7	0	9
K29.0	3	3	0	0
K29.1	9	10	0	1
K29.5	39	41	0.000039	0
K29.7	106	226	0.001109	1
K30	40	74	0.000057	1

K31.9	10	10	0	1
K38.8	27	27	0	0
K38.9	7	7	0	1
K40.20	16	16	0	1
K40.90	12	12	0	0
K40.91	4	4	0	1
K44.0	12	12	0	1
K44.9	45	60	0.000078	1
K45.0	8	8	0	4
K45.8	5	5	0	4
K50.9	3	3	0	1
K51.0	4	4	0	0
K51.4	6	6	0	0
K51.8	6	6	0	4
K51.9	17	18	0.000004	0
K52.2	4	4	0	1
K52.9	13	15	0.000008	1
K58.0	16	22	0.000007	1
K58.9	16	18	0.000007	1
K59.0	21	22	0.000027	1
K59.1	17	18	0.000004	4
K62.2	10	10	0	1
K62.9	5	5	0	0
K70.1	21	24	0.000017	1
K70.9	12	14	0.000001	4
K71.0	19	26	0.000008	0
K71.1	12	12	0	0
K71.2	37	41	0.000027	0
K71.6	3	3	0	0
K71.8	4	4	0	4
K71.9	95	169	0.000832	0
K72.0	38	40	0.000031	0
K72.9	17	18	0.000014	1
K73.0	4	4	0	0
K74.0	56	95	0.000377	1
K74.6	26	30	0.000109	4
K75.0	13	13	0	0
K75.2	2	2	0	0
K76.0	139	299	0.002822	4
K76.1	14	28	0.000007	9
K76.6	18	19	0.000027	1
K76.8	10	13	0.000001	1
K76.9	29	42	0.000045	1
K80.00	8	8	0	9
K80.20	61	181	0.000231	1
K80.80	17	28	0.000006	4

K81.0	12	15	0.000002	4
K81.8	8	8	0	0
K82.2	9	9	0	4
K82.8	14	16	0.000018	9
K82.9	15	16	0.00001	0
K83.5	7	7	0	4
K85	10	10	0	0
K86.1	11	14	0.000005	0
K86.9	14	15	0.000026	0
K91.5	8	8	0	0
L02.9	7	7	0	0
L20.8	7	7	0	1
L20.9	6	7	0	1
L23.8	15	15	0	0
L23.9	4	4	0	1
L28.2	9	10	0	0
L30.9	15	28	0.00001	1
L40.0	14	15	0	1
L40.3	6	6	0	4
L40.5+	6	6	0	4
L40.8	16	16	0	1
L40.9	44	82	0.000072	1
L41.9	6	6	0	1
L50.0	10	10	0	1
L52	7	7	0	0
L56.1	5	5	0	1
L59.9	11	11	0	1
L71.8	13	13	0	1
L80	21	54	0.000004	1
L92.3	6	6	0	9
L94.0	3	3	0	1
L97	9	9	0	0
L98.9	17	40	0.000002	1
M05.20	9	9	0	1
M05.80	9	9	0	0
M06.84	8	8	0	0
M13.0	2	2	0	6
M15.9	17	19	0.000007	1
M16.0	21	35	0.000042	0
M16.9	32	33	0.00001	0
M17.0	13	13	0	0
M17.2	10	10	0	4
M17.9	13	13	0	1
M18.9	18	18	0	1
M19.09	6	6	0	0
M19.99	12	12	0	4

M23.29	6	6	0	4
M25.78	11	11	0	0
M30.1	24	25	0.000016	4
M32.9	22	24	0.000012	1
M35.3	8	8	0	1
M35.8	6	6	0	0
M35.9	3	3	0	0
M40.03	12	12	0	1
M40.04	8	8	0	4
M40.09	8	8	0	0
M40.14	8	8	0	1
M41.05	6	6	0	1
M41.24	13	13	0	1
M41.25	9	9	0	0
M41.39	5	5	0	0
M41.52	5	5	0	0
M41.80	14	16	0.000001	1
M41.84	42	64	0.000052	0
M41.89	27	41	0.000063	1
M41.90	9	9	0	1
M41.94	13	14	0.000001	0
M41.96	8	8	0	0
M41.99	4	4	0	1
M42.00	9	9	0	1
M43.00	11	11	0	0
M43.02	20	24	0.000015	1
M43.03	11	11	0	0
M43.12	12	12	0	4
M43.14	14	14	0	0
M43.17	11	11	0	0
M43.19	7	7	0	1
M47.12	5	5	0	1
M47.80	29	33	0.000025	1
M47.82	35	49	0.00005	1
M47.83	19	19	0	1
M47.84	29	43	0.000007	1
M47.85	7	7	0	9
M47.86	13	13	0	1
M47.88	11	11	0	1
M47.89	23	32	0.000016	1
M47.90	40	72	0.000079	1
M47.92	33	41	0.000078	9
M47.93	12	12	0	1
M47.94	18	23	0.000002	0
M47.95	18	20	0.000006	0
M47.96	28	34	0.000087	9

M47.99	42	48	0.000063	0
M48.47	9	9	0	1
M48.82	6	6	0	4
M48.96	5	5	0	1
M50.0	6	6	0	1
M50.2	6	6	0	1
M51.2	25	34	0.000063	4
M51.3	30	34	0.000087	9
M51.4	11	11	0	0
M51.8	13	13	0	0
M51.9	28	37	0.000018	1
M53.22	7	7	0	0
M53.26	8	14	0	0
M53.89	3	3	0	0
M54.3	5	5	0	1
M54.4	11	11	0	1
M60.88	6	6	0	9
M62.38	6	6	0	9
M62.50	6	6	0	9
M62.59	6	12	0	1
M62.90	10	45	0.000001	1
M70.5	22	34	0.000001	1
M79.00	3	3	0	4
M79.28	16	19	0.000017	0
M79.29	15	16	0.000007	0
M81.00	6	6	0	0
M81.46	7	7	0	1
M81.90	10	10	0	0
M81.99	28	28	0	0
M87.08	9	9	0	1
M96.0	7	7	0	1
N02.9	40	78	0.000071	1
N04.1	8	8	0	1
N08.0*	8	8	0	1
N11.0	27	27	0	0
N11.1	30	35	0.000049	1
N11.9	13	14	0.000003	4
N12	13	14	0.000008	9
N13.3	8	8	0	4
N17.8	9	9	0	0
N17.9	43	60	0.000064	0
N18.0	10	12	0.000004	1
N18.8	27	29	0.000022	4
N18.90	66	103	0.000371	0
N18.91	12	13	0.000001	0
N20.0	73	143	0.000572	0

N20.2	19	24	0.000008	0
N20.9	48	100	0.00014	1
N21.0	16	25	0.000001	1
N21.9	8	8	0	1
N23	6	7	0.000001	9
N28.1	24	36	0.000016	4
N28.8	8	8	0	9
N30.0	38	54	0.000112	1
N30.9	9	11	0.000004	3
N31.0	8	8	0	0
N31.9	9	9	0	0
N32.9	6	6	0	8
N39.0	290	1112	0.016169	0
N39.1	6	6	0	1
N39.4	8	8	0	1
N39.81	12	18	0.000006	1
N39.9	24	26	0.000026	1
N40	23	30	0.000039	0
N60.1	5	5	0	1
N60.2	8	8	0	1
N60.3	22	50	0.000015	1
N60.8	6	6	0	1
N60.9	15	16	0.000009	0
N64.9	6	6	0	0
N72	6	6	0	0
N76.0	14	16	0.00002	9
N76.1	9	9	0	0
N80.4	4	4	0	0
N81.9	8	8	0	1
N83.0	7	7	0	0
N83.2	27	50	0.000041	1
N84.1	5	5	0	1
N85.2	3	3	0	0
N85.8	14	16	0.000008	9
N85.9	4	4	0	1
N99.3	7	7	0	4
O00.0	6	6	0	1
O09.5	5	5	0	0
O92.60	8	8	0	1
O98.5	7	7	0	0
O99.8	5	5	0	0
Q21.0	30	181	0.000036	1
Q21.1	12	12	0	1
Q21.9	16	105	0.000006	1
Q34.0	5	5	0	1
Q38.5	8	8	0	1

Q40.1	13	13	0	1
Q44.6	25	32	0.00004	0
Q50.10	9	9	0	0
Q60.0	6	6	0	0
Q77.81	6	6	0	1
Q79.1	6	6	0	1
Q80.1	9	10	0	1
Q80.9	3	3	0	7
Q89.30	2	2	0	1
R00.0	104	242	0.001242	0
R00.1	18	21	0.000026	0
R04.0	9	11	0.000003	1
R04.2	159	392	0.003584	1
R05	33	62	0.000046	0
R06.0	4	4	0	1
R07.1	15	26	0.000003	1
R07.3	13	13	0	1
R07.4	35	42	0.000086	1
R09.1	69	105	0.000471	0
R09.2	43	67	0.000122	0
R10.3	16	16	0	1
R10.4	10	10	0	0
R13	10	12	0.000002	0
R16.0	15	16	0.000017	0
R16.1	35	44	0.000152	0
R16.2	16	20	0.000003	0
R18	17	17	0	1
R31	68	135	0.000395	1
R35	9	9	0	4
R40.2	35	55	0.00003	1
R41.0	11	11	0	0
R41.2	15	28	0.000007	1
R41.8	27	27	0	0
R42	30	39	0.000039	0
R45.1	11	11	0	0
R49.0	33	45	0.000051	1
R50.0	77	173	0.000626	1
R50.1	32	51	0.000063	0
R50.9	139	388	0.002154	1
R51	40	55	0.000088	0
R52.0	9	9	0	1
R52.1	16	17	0.000008	1
R53	4	4	0	0
R55	16	22	0.000012	0
R56.8	6	6	0	0
R57.9	12	12	0	0

R59.0	45	101	0.00009	1
R59.1	26	28	0.00009	0
R59.9	14	65	0.000001	4
R63.3	25	31	0.000022	4
R64	108	316	0.001373	1
R71	9	9	0	0
R72	15	16	0.000026	0
R73	161	440	0.002498	0
R74.0	163	518	0.003319	0
R74.8	8	8	0	1
R74.9	16	17	0.000005	0
R76.0	5	5	0	0
R76.8	6	6	0	0
R77.0	58	67	0.000178	0
R77.8	8	8	0	0
R77.9	34	39	0.000058	0
R78.0	14	14	0	0
R79.0	21	49	0.000004	1
R79.8	3	3	0	1
R79.9	18	31	0.000007	4
R91	49	63	0.000204	0
R94.2	25	32	0.000032	1
R94.5	30	37	0.000045	0
S00.85	6	6	0	1
S20.2	11	11	0	0
S22.32	9	11	0.000004	0
S22.40	18	20	0.000007	1
S27.88	4	4	0	1
S27.9	14	14	0	0
S31.0	14	14	0	0
S31.1	8	8	0	0
S40.0	12	12	0	1
S52.9	9	9	0	0
S79.9	6	6	0	2
S81.9	6	6	0	1
S91.3	6	6	0	1
T09.05	12	12	0	1
T17.5	24	78	0.000021	4
T17.8	17	20	0.000003	1
T17.9	7	7	0	1
T45.5	7	7	0	0
T65.2	21	25	0.000013	0
T68	8	8	0	1
T75.1	6	6	0	1
T78.2	16	18	0.000008	0
T78.4	129	290	0.002046	0

T80.1	9	10	0.000002	0
T88.7	14	19	0.000011	1
U07.1	133	537	0.002305	0
U07.2	7	7	0	0
W54.0	11	12	0.000007	1
W80	7	7	0	1
Y04.00	12	12	0	1
Y40.0	7	7	0	1
Y40.1	6	6	0	1
Y40.8	11	14	0	4
Y42.0	10	10	0	1
Y83.6	29	33	0.000105	4
Z02.7	8	8	0	0
Z03.0	30	35	0.000038	0
Z03.1	26	36	0.000015	1
Z03.5	7	7	0	0
Z03.9	5	5	0	0
Z11.1	50	76	0.000161	1
Z11.2	6	6	0	1
Z11.5	200	552	0.005997	0
Z12.2	8	8	0	1
Z12.8	10	11	0.000002	0
Z20.9	7	7	0	0
Z22.0	5	5	0	1
Z22.3	22	34	0.000018	4
Z22.51	3	3	0	1
Z32.1	14	21	0.00001	0
Z34.9	6	8	0	0
Z43.0	15	15	0	0
Z45.9	10	10	0	0
Z49.1	11	11	0	0
Z54.0	5	5	0	1
Z59.0	36	42	0.000058	1
Z59.1	27	36	0.00001	1
Z59.9	14	15	0.000009	1
Z60.9	17	17	0	1
Z61.8	5	5	0	1
Z71.3	105	205	0.00175	0
Z71.6	16	16	0	4
Z71.7	14	19	0.000021	0
Z71.8	8	8	0	0
Z72.0	252	1071	0.010362	1
Z72.1	31	42	0.000036	1
Z72.4	6	6	0	1
Z85.3	7	7	0	11
Z85.4	12	12	0	4

Z86.43	13	15	0.000004	0
Z87.18	3	3	0	10
Z87.4	18	18	0	1
Z88.0	11	17	0.000002	1
Z88.1	16	29	0.000009	9
Z88.3	6	6	0	4
Z88.6	19	25	0.000031	0
Z88.8	7	7	0	0
Z88.9	13	15	0.000005	0
Z90.2	12	15	0.000001	0
Z90.3	10	10	0	1
Z90.5	18	23	0.000018	0
Z90.7	7	8	0.000001	0
Z91.0	5	7	0	1
Z92.1	51	85	0.000111	0
Z92.4	26	31	0.000016	0
Z94.0	12	15	0.000002	0
Z95.0	13	13	0	4
Z95.2	8	8	0	1
Z96.0	5	5	0	1
Z96.60	7	15	0.000001	1
Z96.64	13	21	0.000005	0
Z98.0	6	6	0	0
Z99.1	126	474	0.001332	1

Group 50-59

Label	Degree	Weighted Degree	Betweeness centrality	Modularity class
A02.0	7	7	0	0
A04.7	82	138	0.000627	0
A04.9	56	120	0.000265	0
A05.1	4	4	0	9
A07.1	39	54	0.000111	0
A09	70	119	0.000307	0
A15.0	79	127	0.000331	0
A15.2	5	5	0	0
A15.3	7	7	0	0
A15.9	6	6	0	0
A16.0	46	63	0.000071	1
A16.2	22	29	0.000008	1
A16.5	20	30	0.000005	1
A16.9	7	7	0	1
A17.0	7	7	0	0
A18.2	4	4	0	0
A18.8+	23	34	0.00001	1
A41.8	37	54	0.000036	0

A41.9	137	289	0.001332	0
A46	13	15	0.000006	0
A49.0	37	52	0.000057	0
A49.1	18	20	0.000014	0
A49.2	9	9	0	1
B00.9	5	5	0	0
B02.9	14	15	0.000007	0
B15.9	11	11	0	1
B16.9	9	9	0	1
B18.0	21	23	0.000011	7
B18.1	53	100	0.000162	0
B18.2	91	228	0.000766	0
B18.9	15	19	0.000003	1
B20	43	50	0.000058	8
B22	4	4	0	0
B23.8	10	14	0.000005	0
B34.2	8	12	0.000002	0
B36.0	3	3	0	0
B37.0	375	1962	0.019417	0
B37.1	122	346	0.001073	0
B37.4+	10	10	0	1
B37.7	66	146	0.000191	1
B37.88	72	114	0.000261	0
B37.9	12	12	0	1
B44.0	14	21	0.000003	0
B44.1	12	13	0.000001	0
B44.9	5	5	0	0
B67.9	5	5	0	1
B81.1	9	9	0	0
B82.9	10	10	0	0
B90.9	284	1010	0.009478	0
B95.0	12	12	0	0
B95.3	12	12	0	1
B95.42	6	6	0	1
B95.48	31	34	0.000041	0
B95.6	60	109	0.000316	0
B95.7	37	40	0.000042	0
B95.8	19	19	0	0
B96.1	79	148	0.000283	0
B96.2	78	160	0.000281	0
B96.31	12	12	0	0
B96.4	21	21	0	0
B96.5	115	280	0.00078	0
B96.81	9	9	0	0
B96.88	161	796	0.002261	0
B97.1	8	8	0	0

B97.2	159	657	0.001761	0
B99	10	10	0	0
C02.8	16	16	0	1
C07	14	17	0.000005	0
C16.9	17	20	0.000009	0
C22.9	9	9	0	0
C30.0	7	7	0	0
C31.9	11	11	0	0
C32.9	12	12	0	1
C33	9	10	0	0
C34.0	15	21	0.000005	0
C34.1	64	109	0.000179	0
C34.8	28	35	0.000044	0
C34.9	71	126	0.000311	1
C37	6	6	0	0
C38.1	6	6	0	0
C38.3	9	14	0	0
C50.5	5	5	0	0
C50.9	37	56	0.000033	0
C53.9	14	20	0.000004	1
C64	29	54	0.000023	1
C74.9	9	9	0	0
C75.0	5	5	0	0
C77.1	9	10	0	0
C77.9	9	9	0	0
C78.0	37	50	0.000115	0
C78.1	10	14	0	0
C78.2	12	12	0	0
C78.7	17	18	0.000017	0
C79.0	12	12	0	0
C79.3	5	5	0	0
C79.5	6	6	0	1
C79.7	11	11	0	0
C79.88	16	16	0	1
C80	9	9	0	0
C81.9	8	8	0	1
C82.2	23	23	0	0
C82.7	6	6	0	0
C83.0	4	4	0	0
C83.8	9	9	0	0
C88.90	10	17	0	1
C91.10	7	7	0	0
C91.70	4	4	0	0
D00.2	9	9	0	0
D12.6	10	10	0	0
D13.3	10	10	0	1

D14.3	24	35	0.000009	0
D15.2	14	14	0	1
D17.1	8	8	0	0
D17.4	10	10	0	1
D17.9	13	13	0	1
D18.00	18	28	0.000008	0
D18.03	31	36	0.000025	0
D18.08	43	52	0.000026	0
D25.9	60	104	0.000162	1
D26.0	14	15	0.000007	0
D26.9	17	17	0	1
D28.9	9	9	0	12
D30.3	9	9	0	0
D32.0	11	11	0	0
D32.9	21	33	0.000008	0
D35.0	30	44	0.000012	0
D36.7	21	23	0.00001	1
D38.1	48	68	0.000076	0
D38.3	6	6	0	0
D39.9	5	5	0	0
D44.0	13	13	0	1
D45	9	9	0	0
D47.1	9	9	0	8
D47.3	2	2	0	0
D47.7	16	17	0	1
D48.0	14	16	0.000001	0
D48.9	12	12	0	0
D50.0	14	15	0.000012	9
D50.8	106	209	0.000587	0
D50.9	32	40	0.000064	8
D53.0	7	7	0	0
D53.1	5	5	0	0
D53.8	17	29	0	1
D53.9	210	709	0.004218	0
D56.1	5	5	0	0
D58.2	23	60	0.000006	1
D61.0	7	7	0	0
D61.9	12	12	0	1
D62	9	9	0	0
D63.0*	39	56	0.000068	0
D63.8*	52	75	0.000142	1
D64.3	8	8	0	8
D64.8	76	138	0.000218	0
D64.9	172	401	0.00249	0
D68.2	8	8	0	0
D68.8	7	12	0	0

D68.9	31	44	0.000005	1
D69.0	15	20	0.000007	0
D69.1	7	7	0	0
D69.3	10	10	0	0
D69.5	95	141	0.000423	0
D69.6	82	137	0.000401	0
D69.9	14	17	0.000002	0
D70	9	10	0.000001	0
D72.0	18	20	0.000002	0
D72.1	78	150	0.000304	0
D72.8	149	398	0.001141	0
D72.9	30	35	0.000014	1
D73.2	4	4	0	0
D73.9	5	5	0	0
D75.0	14	14	0	1
D75.1	70	175	0.00019	1
D75.2	67	119	0.000206	0
D77*	11	11	0	0
D81.9	20	21	0.000004	0
D84.9	11	11	0	0
D86.0	11	23	0.000003	0
D89.9	9	9	0	0
E01.1	7	7	0	0
E03.0	11	11	0	0
E03.8	19	24	0.000004	0
E03.9	117	260	0.000906	0
E04.1	33	38	0.000029	0
E04.2	9	9	0	0
E04.9	35	56	0.000029	0
E05.0	13	14	0.000001	1
E06.0	6	6	0	0
E06.3	101	187	0.000784	0
E06.5	22	39	0.000034	1
E06.9	19	24	0.000001	0
E07.1	5	5	0	0
E07.8	29	44	0.000021	1
E07.9	15	19	0.000001	0
E09.01	32	75	0.000025	0
E09.9	20	34	0.000004	0
E10.8	15	17	0	1
E10.9	31	42	0.000003	0
E11.01	17	32	0	1
E11.11	7	7	0	1
E11.21	17	28	0.000004	0
E11.34	12	12	0	1
E11.40	17	23	0.000003	0

E11.42	17	17	0	1
E11.49	10	10	0	1
E11.61	11	11	0	1
E11.65	48	80	0.000073	0
E11.69	22	38	0.000009	0
E11.71	7	7	0	0
E11.8	102	226	0.000564	1
E11.9	308	1540	0.010049	0
E13.42	26	26	0	1
E13.9	15	15	0	0
E16.2	14	14	0	0
E16.8	22	33	0.000012	0
E21.4	13	13	0	9
E22.0	25	42	0.000031	0
E22.9	8	8	0	0
E31.9	15	24	0	0
E44.1	46	72	0.000027	1
E55.9	15	15	0	1
E58	45	66	0.00006	0
E65	4	4	0	0
E66.0	384	2072	0.020193	0
E66.2	60	124	0.000107	1
E66.8	69	178	0.000204	1
E66.9	218	768	0.004226	0
E70.0	8	8	0	0
E72.5	1	1	0	0
E75.0	9	9	0	1
E75.6	10	10	0	0
E78.0	257	771	0.007462	0
E78.1	58	76	0.000175	1
E78.2	194	516	0.003248	1
E78.4	20	20	0	1
E78.5	100	258	0.000737	1
E78.8	21	25	0.000003	0
E78.9	44	56	0.000089	1
E79.0	210	697	0.003256	1
E79.9	11	11	0	0
E83.5	7	7	0	1
E83.8	25	42	0.000005	1
E84.9	13	13	0	9
E86	94	192	0.000546	0
E87.0	71	92	0.000185	0
E87.1	162	481	0.002152	0
E87.2	83	143	0.000316	0
E87.3	50	69	0.000086	0
E87.5	92	182	0.000329	0

E87.6	157	332	0.001963	0
E87.8	148	364	0.002542	0
E88.0	27	34	0.000007	0
E88.1	7	7	0	0
E88.8	10	10	0	1
E88.9	78	166	0.000292	0
E89.0	23	31	0.000007	0
F03	10	10	0	0
F06.2	7	7	0	0
F06.3	10	10	0	0
F06.4	10	11	0	0
F06.6	135	385	0.001519	0
F06.7	26	26	0	8
F06.8	14	14	0	0
F10.2	76	132	0.000211	1
F10.5	19	19	0	0
F10.7	8	8	0	0
F17.2	147	359	0.001262	1
F20.0	10	10	0	0
F29	21	26	0.000007	0
F30.2	13	13	0	1
F31.3	22	33	0.000007	1
F31.8	15	19	0.000001	1
F33.0	26	26	0	8
F33.1	11	11	0	0
F33.4	23	27	0.000011	0
F33.8	62	128	0.000197	0
F33.9	103	202	0.000609	0
F41.0	28	75	0.00002	1
F41.1	11	11	0	6
F41.2	190	490	0.003891	0
F41.3	11	11	0	0
F41.8	9	9	0	8
F41.9	122	274	0.000877	0
F48.0	19	28	0.000004	0
F50.8	6	6	0	1
F60.0	11	11	0	0
F61	6	6	0	1
F92.0	26	28	0.000012	0
G00.1	9	9	0	0
G04.2	9	9	0	0
G08	11	11	0	0
G13.8*	26	26	0	1
G20	62	87	0.00013	8
G30.9	19	19	0	1
G31.0	26	26	0	8

G40.10	5	5	0	0
G40.90	40	66	0.000092	12
G41.8	5	5	0	1
G43.3	10	17	0	1
G43.9	11	11	0	6
G45.0	30	34	0.000034	0
G47.30	145	457	0.001734	1
G47.31	40	71	0.000034	0
G47.32	192	789	0.00283	0
G47.33	23	26	0.000003	1
G47.39	49	54	0.000051	0
G47.9	13	13	0	0
G51.8	7	7	0	0
G54.1	18	20	0.000008	0
G54.2	21	31	0.000016	0
G55.2*	9	9	0	1
G56.0	20	20	0	1
G57.0	6	6	0	1
G57.3	15	15	0	0
G58.0	7	7	0	1
G60.8	12	33	0.000001	0
G62.9	8	8	0	1
G70.0	18	23	0.000014	0
G70.1	7	7	0	1
G72.3	6	6	0	0
G80.8	9	9	0	1
G81.9	27	43	0.000062	0
G82.50	6	6	0	0
G93.3	11	12	0.000004	0
G96.8	40	53	0.000025	0
H05.9	7	7	0	13
H10.9	7	7	0	13
H11.0	8	8	0	0
H20.0	19	19	0	0
H20.9	10	10	0	1
H25.9	19	23	0.000007	0
H26.9	14	18	0.000004	1
H31.8	8	8	0	4
H35.8	13	20	0	1
H40.0	11	14	0	9
H40.1	41	73	0.000116	1
H40.2	4	4	0	0
H40.9	28	32	0.000003	8
H44.2	28	36	0.000039	0
H47.0	17	23	0.000002	0
H50.0	19	21	0.000014	9

H52.1	14	16	0.000007	4
H53.4	9	9	0	12
H54.4	5	5	0	1
H81.4	29	36	0.000008	0
H81.9	28	46	0.000014	1
H90.0	49	98	0.000061	0
H90.1	27	48	0.000023	0
H90.2	6	6	0	0
H90.3	14	15	0.000001	1
H90.6	11	11	0	0
H90.8	15	18	0.000002	1
H91.0	10	10	0	0
H91.8	5	5	0	0
H91.9	24	27	0.000016	0
H95.9	17	17	0	0
I05.0	7	7	0	0
I05.1	43	45	0.000033	1
I05.8	26	26	0	8
I05.9	9	9	0	0
I06.2	11	11	0	6
I07.1	123	237	0.000947	1
I07.8	23	35	0.000002	1
I07.9	6	6	0	1
I08.0	22	54	0.000003	0
I08.1	37	59	0.000012	1
I08.9	9	9	0	0
I09.9	7	7	0	0
I10	768	6898	0.143465	0
I11.0	188	738	0.002784	1
I11.9	106	280	0.000694	0
I13.1	8	8	0	0
I15.0	5	5	0	0
I15.8	10	10	0	1
I15.9	15	16	0.000003	0
I20.0	57	104	0.000131	0
I20.8	96	194	0.00052	1
I20.9	141	398	0.001136	1
I21.9	7	7	0	0
I22.9	12	12	0	1
I24.9	59	72	0.000112	8
I25.0	78	182	0.000274	0
I25.10	9	9	0	0
I25.2	83	210	0.000282	1
I25.5	122	350	0.001145	0
I25.6	27	41	0.000016	1
I25.8	73	152	0.000275	0

I25.9	321	1789	0.011769	1
I26.0	24	26	0.000008	0
I26.9	44	152	0.000064	1
I27.0	189	975	0.002316	1
I27.2	48	68	0.000068	1
I27.8	29	60	0.000011	1
I27.9	270	1465	0.006506	1
I30.0	18	22	0.000002	1
I30.1	13	15	0.000004	1
I30.9	47	63	0.000115	1
I31.9	22	22	0	1
I32.0*	11	11	0	1
I33.9	5	5	0	0
I34.0	252	834	0.005882	1
I34.1	15	18	0.000001	1
I34.2	15	16	0.000006	0
I35.0	38	43	0.000053	0
I35.1	88	174	0.000397	1
I35.8	26	26	0	8
I36.0	38	70	0.000054	0
I36.1	148	484	0.001339	1
I36.9	10	10	0	0
I37.1	28	34	0.000018	1
I38	15	15	0	1
I42.0	23	35	0.000005	1
I42.2	8	8	0	0
I44.0	7	7	0	0
I44.7	49	70	0.000063	0
I45.0	136	528	0.001031	1
I45.1	10	10	0	1
I45.5	10	10	0	1
I45.8	30	46	0.000019	1
I45.9	25	37	0.000002	1
I46.0	8	8	0	0
I46.9	77	151	0.000268	0
I47.1	149	308	0.001411	1
I47.2	23	26	0.000004	1
I47.9	34	44	0.000031	1
I48	146	458	0.001409	1
I49.0	30	54	0.000018	1
I49.1	50	91	0.000134	0
I49.3	92	181	0.000485	1
I49.4	30	46	0.000011	1
I49.5	26	42	0.000026	0
I49.8	45	81	0.000061	0
I49.9	9	11	0	0

I50.0	283	1745	0.007694	1
I50.1	21	23	0.000005	0
I50.9	166	473	0.001804	1
I51.7*	42	53	0.000039	0
I51.9	12	13	0.000002	1
I62.9	15	15	0	1
I63.8	45	47	0.000119	8
I63.9	18	19	0.000005	0
I64	29	48	0.000048	0
I65.2	8	8	0	0
I65.9	8	8	0	1
I67.2	10	10	0	0
I67.8	9	16	0	0
I67.9	58	81	0.000093	0
I69.2	7	7	0	5
I69.3	20	20	0	1
I69.4	20	24	0.000004	1
I69.8	15	15	0	1
I70.0	112	220	0.000729	1
I70.21	10	10	0	11
I70.8	37	47	0.000049	1
I70.9	81	144	0.00025	1
I71.2	18	27	0.000005	1
I71.9	12	12	0	1
I72.8	9	9	0	12
I72.9	12	19	0.000001	12
I73.9	34	62	0.000029	0
I74.8	9	9	0	1
I77.6	7	7	0	0
I77.9	10	10	0	1
I78.8	11	11	0	1
I79.0*	11	11	0	1
I80.2	6	6	0	1
I80.3	44	87	0.000045	1
I80.8	8	8	0	0
I80.9	10	10	0	0
I82.2	8	8	0	0
I82.8	28	32	0.000004	0
I83.0	14	14	0	1
I83.1	59	124	0.000165	0
I83.2	6	6	0	0
I83.9	70	179	0.000195	1
I84.9	52	64	0.000102	0
I85.9	16	16	0	7
I86.8	8	8	0	0
I87.0	34	63	0.000027	1

I87.2	162	436	0.002262	0
I87.8	17	31	0.000002	0
I87.9	13	26	0.000002	0
I88.9	7	7	0	0
I95.9	15	24	0	1
I97.2	10	10	0	0
J00	11	11	0	0
J01.0	36	43	0.000053	1
J01.1	18	20	0.00001	1
J01.4	9	9	0	0
J01.8	7	7	0	5
J02.9	27	40	0.00002	0
J03.9	16	21	0.000007	0
J04.0	15	16	0.000001	1
J06.9	27	34	0.000006	1
J10.0	18	20	0.000006	0
J10.8	7	7	0	0
J11.1	16	18	0.000008	3
J12.1	9	9	0	0
J12.8	64	158	0.000192	0
J12.9	162	494	0.00221	0
J13	12	13	0.000001	1
J14	12	12	0	0
J15.0	71	141	0.000179	1
J15.1	53	96	0.000112	0
J15.2	35	54	0.000032	0
J15.4	39	71	0.000071	0
J15.5	19	34	0.000006	0
J15.6	50	90	0.000145	0
J15.8	298	1513	0.010576	0
J15.9	203	698	0.003476	0
J16.8	13	15	0.000001	0
J18.0	164	549	0.001825	0
J18.1	7	7	0	0
J18.8	104	236	0.000574	1
J18.9	272	1299	0.008851	0
J20.8	13	13	0	0
J20.9	19	26	0.000014	0
J21.0	11	11	0	0
J22	9	9	0	0
J30.0	11	14	0.000001	0
J30.1	131	357	0.001619	0
J30.2	11	13	0.000001	0
J30.3	27	52	0.00001	0
J30.4	211	693	0.004597	0
J31.0	152	473	0.001849	0

J31.1	8	8	0	4
J31.2	8	8	0	0
J32.0	42	62	0.000089	0
J32.1	26	32	0.000007	0
J32.8	8	15	0	1
J32.9	85	176	0.000403	0
J33.0	14	18	0.000001	0
J33.1	12	13	0.000004	0
J33.9	51	70	0.000162	0
J34.0	8	8	0	0
J34.2	62	122	0.000222	0
J34.8	13	15	0.000001	0
J36	8	8	0	0
J37.0	68	121	0.000148	1
J38.3	12	12	0	0
J39.9	1	1	0	0
J40	7	7	0	0
J41.0	69	105	0.000203	1
J41.1	20	22	0.000003	0
J41.8	20	38	0.000009	0
J42	63	89	0.000209	1
J43.1	20	22	0.000003	0
J43.2	16	21	0.000003	3
J43.8	34	62	0.000065	1
J43.9	111	274	0.00073	0
J44.0	330	2027	0.012344	1
J44.1	241	1088	0.006079	1
J44.8	85	147	0.000371	1
J44.9	172	409	0.00274	1
J45	997	11445	0.386981	0
J46	57	122	0.000132	0
J47	292	1387	0.010506	0
J60	6	6	0	10
J66.1	10	10	0	1
J67.7	20	20	0	1
J67.9	15	18	0.000001	0
J68.4	7	7	0	1
J69.1	8	8	0	1
J70.4	12	12	0	3
J81	25	34	0.000018	0
J82	27	42	0.000011	0
J84.0	14	17	0.000004	0
J84.1	212	682	0.004489	0
J84.8	48	83	0.000126	0
J84.9	253	828	0.00748	0
J85.1	16	30	0.000004	0

J85.2	6	6	0	0
J86.0	19	21	0.000004	0
J86.9	7	7	0	1
J90	116	283	0.001279	0
J91*	44	55	0.000049	1
J92.0	73	159	0.000375	0
J92.9	163	392	0.001878	1
J93.0	7	10	0	0
J93.8	14	14	0	1
J93.9	20	25	0.000019	0
J94.1	27	40	0.000016	1
J94.8	82	153	0.000342	1
J94.9	32	37	0.000011	1
J95.1	7	7	0	0
J95.8	23	31	0.000004	1
J95.9	17	19	0.000005	1
J96.0	580	4590	0.06643	0
J96.1	439	2945	0.030028	1
J96.9	16	16	0	7
J98.0	14	14	0	0
J98.1	31	48	0.00003	0
J98.3	10	10	0	1
J98.4	28	35	0.000049	0
J98.5	13	13	0	1
J98.6	6	6	0	1
J98.8	65	130	0.000237	1
J98.9	22	36	0.000024	1
K21.0	93	220	0.000794	0
K21.9	114	243	0.001028	0
K22.8	5	5	0	1
K25.3	4	4	0	0
K25.5	8	8	0	0
K25.7	24	28	0.00001	0
K25.9	33	50	0.000041	1
K26.0	11	11	0	9
K26.3	18	27	0.000003	0
K26.7	25	39	0.000015	0
K27.3	8	8	0	1
K27.7	18	27	0.000004	0
K28.0	5	5	0	1
K28.1	5	5	0	1
K28.9	34	73	0.000027	1
K29.0	10	10	0	0
K29.1	16	19	0	1
K29.2	7	7	0	0
K29.5	67	112	0.000271	1

K29.6	6	6	0	0
K29.7	74	140	0.000309	1
K29.9	10	10	0	1
K30	78	163	0.000317	1
K31.88	6	6	0	0
K40.90	6	6	0	0
K42.9	7	7	0	7
K43.9	8	8	0	1
K44.9	89	146	0.000452	0
K45.8	8	9	0.000001	0
K46.9	12	19	0.000001	12
K51.0	20	22	0.000002	0
K51.9	15	33	0.000001	0
K52.8	12	12	0	0
K52.9	28	36	0.00002	1
K57.10	6	6	0	0
K57.30	9	9	0	0
K57.32	13	13	0	0
K58.0	55	67	0.000055	1
K58.9	8	8	0	0
K59.0	49	78	0.000064	1
K59.1	20	22	0.000013	0
K59.3	13	13	0	1
K60.2	14	14	0	0
K61.0	12	12	0	1
K62.9	10	10	0	0
K63.50	6	6	0	0
K70.1	36	56	0.000024	1
K70.3	17	34	0.000002	1
K70.9	79	132	0.000388	0
K71.0	35	48	0.00002	0
K71.1	12	12	0	3
K71.9	82	179	0.000222	0
K72.0	30	33	0.000009	0
K72.1	11	11	0	0
K72.9	17	18	0.000005	0
K73.0	10	19	0	1
K73.2	26	26	0	1
K73.8	7	7	0	0
K73.9	31	52	0.000013	0
K74.0	70	163	0.000259	1
K74.6	37	49	0.000048	0
K75.0	13	15	0.000001	0
K76.0	253	843	0.007094	0
K76.1	11	11	0	1
K76.8	6	6	0	1

K76.9	38	79	0.000052	1
K80.00	7	7	0	0
K80.01	4	4	0	0
K80.10	11	11	0	0
K80.11	21	21	0	1
K80.20	68	100	0.000208	1
K80.21	6	6	0	0
K80.80	31	41	0.000048	1
K81.0	10	10	0	0
K81.1	12	12	0	0
K81.8	28	30	0.000015	1
K82.8	37	78	0.000064	1
K83.5	26	30	0.00003	0
K83.8	4	4	0	0
K85	12	14	0.000003	0
K91.1	14	14	0	1
K91.5	36	58	0.000034	1
L03.11	9	9	0	0
L20.9	9	11	0.000003	0
L23.0	8	8	0	0
L23.9	2	2	0	0
L27.1	7	7	0	1
L30.9	20	23	0.00001	0
L40.0	33	47	0.000042	1
L40.8	9	9	0	0
L40.9	49	66	0.000122	1
L50.0	9	9	0	0
L50.1	10	10	0	0
L50.8	7	7	0	0
L50.9	6	6	0	0
L60.9	8	8	0	1
L89	13	13	0	1
L91.0	10	10	0	0
L94.1	9	9	0	0
L94.9	6	6	0	10
L95.8	10	13	0.000004	5
L97	10	10	0	11
L98.6	15	15	0	1
M05.80	10	10	0	1
M05.89	11	11	0	0
M05.99	13	13	0	0
M06.40	9	9	0	8
M06.98	8	8	0	0
M06.99	9	9	0	0
M08.10	8	8	0	1
M10.00	10	10	0	1

M10.99	42	58	0.000048	0
M13.0	65	122	0.000199	1
M15.8	21	34	0.000012	1
M15.9	73	149	0.000335	1
M16.0	20	38	0.000009	0
M16.9	26	27	0	8
M17.0	38	61	0.000078	0
M17.1	10	12	0.000002	0
M17.4	15	17	0.000001	0
M17.9	24	28	0.000022	0
M19.99	10	11	0.000001	0
M20.0	8	8	0	1
M20.2	6	6	0	0
M30.0	6	6	0	0
M30.1	13	13	0	0
M32.9	12	12	0	0
M34.9	12	12	0	1
M35.9	19	26	0.000012	0
M40.00	12	16	0.000003	0
M40.04	25	65	0.000021	1
M40.09	6	10	0	1
M40.10	5	5	0	1
M40.12	20	20	0	1
M40.14	10	10	0	0
M40.15	6	6	0	1
M40.22	7	7	0	0
M40.50	5	5	0	1
M41.14	20	22	0.000008	1
M41.25	4	4	0	0
M41.34	9	15	0	0
M41.35	6	6	0	0
M41.49	14	14	0	1
M41.54	8	8	0	0
M41.80	5	5	0	1
M41.84	13	22	0	1
M41.85	24	27	0.000008	0
M41.86	4	4	0	0
M41.89	9	26	0	1
M41.90	15	17	0.000004	0
M41.99	12	12	0	0
M43.02	17	18	0.000007	6
M43.09	22	22	0	0
M43.12	18	19	0.000003	0
M43.16	6	6	0	0
M45.09	15	17	0.000007	1
M47.14	7	7	0	0

M47.18	17	17	0	0
M47.20	9	21	0	0
M47.22	12	12	0	0
M47.80	71	142	0.000282	0
M47.82	86	149	0.000433	0
M47.83	19	27	0.000006	1
M47.84	51	93	0.000132	0
M47.86	55	86	0.000126	0
M47.87	8	8	0	0
M47.89	56	78	0.000095	0
M47.90	104	275	0.000588	1
M47.92	70	113	0.000306	0
M47.94	26	50	0.000021	0
M47.95	15	18	0.000003	3
M47.96	10	10	0	1
M47.99	43	57	0.000076	1
M48.50	8	8	0	0
M48.86	9	9	0	2
M50.9	6	6	0	0
M51.0	37	42	0.000092	1
M51.1+	6	6	0	0
M51.2	49	157	0.000104	1
M51.3	14	19	0.000001	0
M51.8	10	10	0	0
M51.9	48	75	0.000034	0
M53.26	16	16	0	7
M53.86	14	15	0.000001	1
M53.90	12	21	0	0
M53.99	10	17	0	0
M54.16	8	8	0	0
M54.3	11	11	0	0
M54.4	40	60	0.00006	0
M54.84	19	19	0	1
M54.90	10	10	0	1
M62.90	9	13	0.000001	0
M65.4	10	10	0	1
M75.0	5	5	0	0
M75.9	14	14	0	0
M79.09	7	7	0	0
M79.28	42	70	0.000066	0
M80.90	13	13	0	1
M81.00	32	50	0.000032	1
M81.09	12	14	0.000006	0
M81.59	11	11	0	0
M81.88	12	20	0.000004	1
M81.90	28	35	0.000059	0

M81.98	32	53	0.000023	1
M81.99	23	29	0.000012	0
M85.40	12	21	0	1
M85.90	12	17	0.000005	0
M85.99	4	4	0	1
M86.00	4	4	0	1
N02.9	47	90	0.000101	0
N03.9	13	13	0	9
N10	17	17	0	1
N11.0	10	10	0	0
N11.1	29	30	0.000017	0
N12	21	23	0.000005	1
N13.3	27	46	0.000014	0
N17.8	33	47	0.000024	0
N17.9	79	142	0.000277	0
N18.8	10	11	0.000003	0
N18.90	111	267	0.000715	1
N19	8	10	0	0
N20.0	100	183	0.000529	0
N20.1	12	12	0	1
N20.2	18	25	0.000006	0
N20.9	90	155	0.000379	0
N21.0	9	9	0	0
N28.1	88	134	0.000517	1
N30.0	47	66	0.000095	0
N30.9	19	19	0	0
N39.0	354	1731	0.019036	0
N39.1	7	7	0	0
N39.81	19	28	0.000004	0
N39.9	9	9	0	0
N40	59	100	0.000135	1
N43.3	9	9	0	0
N60.1	23	37	0.000002	1
N60.2	26	34	0.000005	1
N60.8	10	10	0	0
N61	11	11	0	1
N63	9	9	0	2
N64.9	18	20	0.000006	0
N72	10	10	0	0
N81.0	4	4	0	0
N83.2	10	13	0.000001	0
N84.1	9	9	0	0
N85.0	9	9	0	2
N92.6	11	11	0	0
N95.3	8	8	0	1
N99.3	47	61	0.000134	1

N99.8	10	10	0	1
N99.9	15	21	0.000001	1
Q07.0	9	9	0	8
Q21.0	20	28	0.000006	1
Q21.1	27	37	0.000024	1
Q21.9	9	47	0	1
Q25.8	13	13	0	1
Q40.1	26	43	0.000031	0
Q44.6	22	25	0.000006	1
Q61.0	37	47	0.000055	0
Q61.8	26	26	0	8
Q61.9	22	24	0.000009	0
Q63.89	6	6	0	1
Q85.0	8	8	0	1
Q89.35	8	8	0	0
R00.0	81	167	0.00032	0
R00.1	30	38	0.000007	1
R04.0	9	9	0	1
R04.2	145	318	0.001557	0
R05	26	49	0.000005	0
R06.0	2	2	0	0
R07.1	21	28	0.000007	1
R07.2	6	6	0	1
R07.3	6	6	0	5
R07.4	34	46	0.000048	1
R09.1	125	215	0.000966	0
R09.2	33	65	0.000031	0
R09.8	12	12	0	1
R10.0	18	19	0.000012	12
R10.1	19	19	0	1
R10.2	9	9	0	1
R10.4	14	14	0	1
R11	14	14	0	1
R16.0	40	100	0.00005	0
R16.1	40	84	0.000069	0
R16.2	17	22	0.000002	0
R20.2	17	17	0	0
R23.3	18	18	0	0
R30.0	12	12	0	1
R31	83	173	0.000327	0
R32	8	8	0	0
R33	9	9	0	0
R39.0	8	8	0	0
R40.2	26	37	0.000008	1
R41.2	6	6	0	0
R42	50	64	0.000101	0

R47.0	7	7	0	0
R49.0	9	11	0.000003	0
R50.0	73	135	0.000195	0
R50.1	14	16	0.000005	0
R50.9	122	297	0.000879	0
R51	65	95	0.000216	0
R55	21	24	0.000017	9
R56.8	29	38	0.000003	0
R58	9	9	0	1
R59.0	55	109	0.000211	0
R59.1	6	7	0	0
R59.9	27	38	0.00003	0
R61.1	14	14	0	0
R63.3	12	13	0.000005	0
R63.4	21	21	0	0
R64	104	236	0.000702	1
R72	22	27	0.000002	0
R73	231	832	0.005054	0
R74.0	192	595	0.003041	0
R76.0	15	18	0.000001	0
R77.0	43	52	0.000084	0
R77.8	16	17	0.000009	0
R77.9	15	15	0	1
R79.0	24	32	0.000012	0
R84.9	6	6	0	1
R90.0	6	6	0	0
R91	44	66	0.000066	1
R94.2	34	56	0.000047	1
R94.5	95	157	0.000432	1
S20.2	7	7	0	0
S22.32	18	21	0.000002	0
S27.6	13	15	0.000003	0
S42.09	7	7	0	0
S72.00	6	6	0	1
S80.0	7	7	0	0
S82.9	3	3	0	1
S85.7	4	6	0	1
T17.5	6	6	0	0
T45.5	11	11	0	0
T48.0	12	12	0	0
T65.2	20	20	0	1
T78.2	4	4	0	0
T78.4	85	153	0.00048	0
T87.4	10	10	0	11
T88.7	36	44	0.000046	0
T88.9	4	4	0	0

T91.2	17	19	0.000004	0
U07.1	165	677	0.002109	0
U07.2	24	30	0.000006	0
W80	6	6	0	0
Y36.0	10	10	0	0
Y40.8	8	8	0	1
Y43.4	13	13	0	0
Y44.2	17	28	0	1
Y83.6	15	15	0	0
Y83.8	8	8	0	0
Y84.1	12	12	0	0
Z01.5	11	12	0.000001	0
Z03.0	34	40	0.000016	0
Z03.1	22	29	0.000012	0
Z11.1	29	56	0.000008	0
Z11.5	220	713	0.004363	0
Z12.2	6	6	0	0
Z12.8	9	11	0.000001	0
Z12.9	7	7	0	0
Z20.9	24	30	0.000006	0
Z41.9	6	6	0	0
Z45.8	7	7	0	1
Z59.0	79	170	0.000196	1
Z59.1	5	5	0	1
Z59.5	7	7	0	0
Z71.3	88	230	0.000558	0
Z71.6	13	14	0	0
Z71.8	18	22	0.000004	0
Z72.0	223	911	0.004445	0
Z72.1	69	169	0.000178	0
Z75.11	15	15	0	1
Z82.3	12	12	0	0
Z85.3	23	26	0.000017	0
Z85.4	14	19	0.000005	0
Z86.11	6	6	0	0
Z87.11	19	19	0	1
Z87.12	13	13	0	0
Z88.0	28	31	0.00002	1
Z88.1	36	44	0.000046	1
Z88.6	20	32	0.000007	0
Z88.8	6	6	0	0
Z88.9	13	13	0	1
Z89.9	10	10	0	11
Z90.0	8	8	0	0
Z90.2	18	21	0.000012	0
Z90.5	19	19	0	0

Z90.7	26	29	0.000031	1
Z92.1	79	136	0.000188	1
Z92.4	20	26	0.000005	0
Z92.8	8	8	0	0
Z92.9	6	6	0	0
Z95.2	8	8	0	1
Z95.4	13	13	0	1
Z95.9	13	19	0	1
Z96.64	12	12	0	0
Z97.4	9	9	0	0
Z99.1	186	890	0.002456	1
Z99.2	11	11	0	1

Group 60-69

**Label**	**Degree**	**Weighted Degree**	**Betweeness centrality**	**Modularity class**
A02.0	14	16	0.000002	0
A04.0	8	8	0	1
A04.5	14	14	0	0
A04.7	207	511	0.001604	0
A04.9	138	265	0.000784	0
A05.1	9	9	0	1
A05.9	5	5	0	0
A07.1	50	70	0.000074	0
A09	108	226	0.000288	0
A15.0	110	194	0.000231	0
A15.1	39	75	0.000019	0
A15.3	46	102	0.000042	0
A16.0	60	95	0.00005	1
A16.1	17	19	0.000001	0
A16.2	52	76	0.000101	1
A16.5	5	5	0	1
A16.8	12	12	0	0
A18.2	11	11	0	0
A26.0	19	19	0	1
A41.0	14	14	0	0
A41.1	35	39	0.00001	0
A41.50	12	19	0	0
A41.51	15	15	0	0
A41.8	131	311	0.000535	1
A41.9	243	778	0.002289	0
A42.0	8	8	0	6
A42.1	18	18	0	0
A46	16	21	0	0
A48.3	12	12	0	0
A49.0	27	41	0.000019	0

A63.0	18	18	0	0
B02.9	13	16	0.000003	0
B05.2	15	15	0	1
B15.9	14	14	0	0
B16.9	6	6	0	0
B17.1	7	7	0	1
B18.0	21	23	0.000003	0
B18.1	148	277	0.001059	0
B18.2	150	386	0.000919	0
B18.9	19	22	0.000003	1
B19.9	19	21	0.000001	0
B34.1	13	13	0	1
B34.2	52	97	0.000074	0
B35.1	8	8	0	0
B36.0	10	19	0	1
B36.9	13	13	0	0
B37.0	502	3038	0.018809	1
B37.1	178	647	0.001086	1
B37.2	7	7	0	0
B37.3+	13	13	0	0
B37.4+	10	10	0	1
B37.7	63	100	0.000133	0
B37.81	11	13	0	1
B37.88	59	76	0.000042	0
B37.9	62	80	0.000024	0
B39.0	10	10	0	1
B44.1	5	5	0	1
B47.9	10	17	0	1
B67.0	6	6	0	1
B90.2	26	40	0.000004	1
B90.9	365	1864	0.007021	1
B95.1	8	8	0	1
B95.3	14	16	0.000001	1
B95.48	6	6	0	0
B95.5	7	7	0	1
B95.6	48	88	0.000016	1
B95.7	33	37	0.000005	0
B96.0	12	12	0	0
B96.1	59	115	0.000032	1
B96.2	182	466	0.000982	1
B96.38	7	7	0	1
B96.4	16	18	0.000002	1
B96.5	126	310	0.000381	1
B96.6	5	5	0	1
B96.7	11	11	0	0
B96.81	12	21	0	0

B96.88	235	1298	0.002336	1
B97.1	29	40	0.000005	0
B97.2	243	1147	0.002686	0
B99	51	85	0.000052	0
C00.2	12	12	0	1
C01	11	11	0	1
C02.9	18	18	0	0
C15.9	6	6	0	1
C16.9	29	35	0.000013	0
C18.2	20	22	0.000009	0
C18.4	17	17	0	0
C18.7	13	13	0	1
C18.9	43	71	0.000014	1
C32.1	6	6	0	0
C32.8	9	12	0.000022	1
C32.9	9	9	0	1
C34.0	40	55	0.00003	1
C34.1	84	153	0.000163	0
C34.2	26	32	0.000015	1
C34.3	35	52	0.000021	1
C34.8	35	49	0.000021	1
C34.9	163	449	0.000734	1
C38.2	19	20	0.000002	0
C38.3	43	104	0.000017	1
C38.4	6	6	0	1
C39.8	4	4	0	1
C40.9	17	17	0	1
C43.8	15	15	0	0
C43.9	9	9	0	0
C48.0	13	13	0	0
C50.1	11	11	0	0
C50.8	9	9	0	1
C50.9	90	170	0.000248	0
C53.9	29	34	0.000013	0
C54.9	16	16	0	0
C55	11	11	0	0
C61	28	29	0.000003	2
C64	25	27	0.000007	0
C67.9	27	29	0.000012	0
C69.6	5	5	0	1
C69.9	15	15	0	1
C73	21	21	0	0
C76.1	17	17	0	1
C77.1	72	96	0.000116	1
C78.0	66	112	0.000116	0
C78.1	34	44	0.000019	1

C78.2	24	31	0.000006	1
C78.7	37	43	0.000027	1
C78.8	8	8	0	0
C79.2	13	13	0	1
C79.5	28	31	0.000004	0
C79.7	52	63	0.000049	1
C79.88	11	11	0	1
C80	48	63	0.000014	1
C81.9	14	16	0	0
C82.7	10	10	0	1
C82.9	18	24	0.000001	0
C83.8	12	12	0	0
C85.7	14	14	0	0
C90.00	6	6	0	0
C91.01	6	6	0	1
C91.10	27	48	0.000001	1
C92.10	7	7	0	0
D09.3	12	12	0	0
D13.5	19	19	0	0
D14.3	52	58	0.000049	0
D14.4	13	13	0	0
D15.2	20	20	0	1
D16.9	11	11	0	0
D17.9	15	15	0	0
D18.00	17	17	0	0
D18.01	8	8	0	4
D18.03	12	12	0	0
D18.08	20	33	0.000002	0
D22.9	15	15	0	0
D25.9	66	87	0.000046	0
D29.1	63	82	0.000081	0
D32.0	18	18	0	0
D32.9	34	44	0.000004	0
D34	32	38	0.000012	0
D35.0	60	80	0.000051	0
D36.0	4	4	0	1
D37.1	17	17	0	0
D38.1	99	173	0.000142	0
D38.2	26	31	0.000006	1
D38.3	24	25	0.000002	1
D38.5	11	11	0	0
D40.0	7	7	0	8
D44.0	41	53	0.000009	0
D44.1	34	41	0.000006	0
D44.4	14	14	0	1
D44.7	25	25	0	0

D46.0	19	19	0	0
D46.4	37	54	0.000055	0
D47.3	14	14	0	0
D47.7	30	38	0.000012	0
D47.9	37	46	0.000009	0
D48.1	15	15	0	0
D48.9	11	11	0	0
D50.0	23	29	0.000004	0
D50.8	122	318	0.000561	1
D50.9	138	291	0.000446	0
D51.8	10	10	0	1
D53.0	18	22	0.000003	1
D53.8	10	11	0	1
D53.9	349	1681	0.006386	1
D56.1	12	15	0.000001	1
D58.2	45	104	0.000011	1
D60.0	3	3	0	0
D61.0	26	30	0.000005	0
D62	22	22	0	0
D63.0*	73	128	0.000126	1
D63.8*	77	187	0.000112	1
D64.0	31	39	0.000014	0
D64.1	16	16	0	0
D64.3	8	8	0	1
D64.8	131	330	0.000384	1
D64.9	304	1092	0.00472	0
D68.3	16	16	0	0
D68.8	29	48	0.000006	0
D68.9	11	11	0	0
D69.3	6	6	0	0
D69.4	14	14	0	0
D69.5	144	261	0.000787	0
D69.6	125	235	0.000398	0
D69.8	12	17	0	1
D70	17	19	0.000005	0
D71	9	10	0.000002	1
D72.1	80	172	0.000132	1
D72.8	237	765	0.001889	1
D72.9	53	66	0.000047	0
D73.1	22	23	0.00001	0
D73.3	13	13	0	0
D73.9	10	10	0	0
D75.0	23	27	0.000002	1
D75.1	19	26	0.000001	1
D75.2	120	218	0.00042	0
D75.9	10	10	0	1

D76.1	13	13	0	1
D77*	9	9	0	5
D80.2	7	7	0	1
D80.9	7	7	0	1
D81.9	9	14	0	0
D82.9	14	14	0	0
D86.0	11	20	0.000001	0
D86.2	16	16	0	0
D86.8	16	16	0	0
D89.1	7	7	0	0
E01.0	13	13	0	0
E01.1	6	6	0	0
E01.2	2	2	0	1
E03.1	13	13	0	0
E03.8	29	32	0.000007	0
E03.9	142	403	0.000531	1
E04.1	76	114	0.000153	0
E04.2	9	9	0	0
E04.8	5	5	0	3
E04.9	16	16	0	1
E05.9	34	45	0.000005	1
E06.3	141	265	0.000679	0
E06.5	18	18	0	1
E06.9	41	58	0.000018	1
E07.1	8	8	0	1
E07.8	39	66	0.000014	1
E07.9	45	58	0.000031	0
E09.01	30	47	0.000009	1
E09.9	32	74	0.000012	1
E10.36	7	7	0	1
E10.39	10	10	0	0
E10.8	3	3	0	1
E10.9	43	62	0.000013	1
E11.01	7	13	0	0
E11.02	4	4	0	1
E11.13	20	20	0	1
E11.21	21	23	0	1
E11.22	47	65	0.000015	0
E11.34	20	22	0.000004	0
E11.35	13	13	0	1
E11.40	13	13	0	0
E11.42	90	143	0.000103	0
E11.49	7	7	0	0
E11.51	7	7	0	0
E11.65	93	186	0.000148	1
E11.69	119	251	0.000344	0

E11.72	6	6	0	0
E11.8	245	842	0.00242	1
E11.9	535	3632	0.022464	1
E13.9	5	5	0	0
E14.65	10	10	0	0
E14.9	4	4	0	0
E16.2	32	35	0.000018	0
E16.8	12	12	0	0
E16.9	27	30	0.000004	0
E21.4	18	20	0.000003	0
E23.0	7	7	0	10
E23.3	13	13	0	0
E23.6	24	30	0.000005	0
E24.2	12	19	0	1
E26.0	13	13	0	0
E27.2	13	13	0	0
E27.9	15	22	0.000001	1
E28.2	9	9	0	1
E44.0	11	11	0	0
E44.1	17	24	0.000001	1
E55.9	16	16	0	0
E58	7	7	0	1
E61.2	3	3	0	0
E64.8	14	14	0	1
E65	7	7	0	1
E66.0	469	3296	0.014241	1
E66.1	19	23	0.000002	1
E66.2	87	182	0.000097	1
E66.8	37	77	0.000016	1
E66.9	297	1373	0.004023	0
E68	8	14	0	1
E70.0	8	8	0	1
E70.9	19	19	0	0
E72.2	11	11	0	0
E72.5	10	11	0.000001	1
E72.9	10	10	0	1
E73.9	6	6	0	1
E74.4	6	6	0	0
E75.5	12	19	0	1
E75.6	64	125	0.000053	0
E77.8	7	7	0	0
E78.0	297	1100	0.005224	0
E78.1	64	103	0.000097	1
E78.2	202	564	0.001605	1
E78.3	23	25	0.000003	1
E78.4	18	18	0	0

E78.5	111	351	0.000332	1
E78.6	13	13	0	1
E78.8	35	60	0.000023	0
E78.9	24	31	0.000001	1
E79.0	311	1310	0.004013	1
E79.8	10	10	0	0
E80.1	8	8	0	0
E83.0	12	12	0	1
E83.8	13	13	0	0
E84.9	13	13	0	0
E85.9	16	16	0	0
E86	199	480	0.001324	0
E87.0	114	259	0.000216	1
E87.1	269	886	0.002888	0
E87.2	185	464	0.000895	0
E87.3	109	182	0.000249	0
E87.5	178	440	0.000775	0
E87.6	261	882	0.002689	0
E87.7	14	16	0.000004	0
E87.8	302	943	0.005852	0
E88.0	6	6	0	0
E88.2	8	8	0	1
E88.9	103	247	0.000223	1
E89.0	29	38	0.000035	1
F01.1	7	7	0	1
F01.2	10	10	0	2
F01.3	29	34	0.000013	0
F01.9	14	14	0	1
F03	26	29	0.000005	1
F06.0	8	8	0	0
F06.4	16	18	0.000002	1
F06.6	192	633	0.001522	0
F06.7	35	45	0.000011	0
F06.8	8	8	0	0
F06.9	12	12	0	0
F07.0	21	21	0	0
F07.9	8	8	0	1
F10.1	16	18	0.000003	1
F10.2	73	156	0.00007	1
F10.3	15	15	0	1
F10.5	14	15	0	1
F13.3	9	9	0	0
F17.0	6	6	0	1
F17.1	31	34	0.000005	0
F17.2	159	555	0.000728	1
F17.3	7	7	0	1

F17.9	15	15	0	1
F20.0	8	8	0	1
F20.3	8	8	0	0
F23.9	11	11	0	1
F25.2	6	6	0	1
F31.3	64	123	0.000052	1
F31.4	7	7	0	8
F31.8	15	22	0.000001	1
F31.9	5	5	0	1
F32.00	10	10	0	0
F32.90	14	18	0.000001	0
F33.0	33	42	0.000005	0
F33.1	18	21	0.000001	1
F33.4	12	12	0	0
F33.8	108	214	0.000289	1
F33.9	180	456	0.0011	0
F38.8	10	10	0	1
F39	9	9	0	1
F41.0	41	56	0.000019	1
F41.1	36	46	0.000008	0
F41.2	231	686	0.002114	0
F41.8	6	6	0	0
F41.9	211	647	0.001749	0
F44.9	13	13	0	0
F45.0	22	25	0.000001	1
F45.30	4	4	0	1
F45.33	11	11	0	0
F45.4	7	7	0	1
F48.0	25	44	0.000016	0
F60.0	18	29	0	1
F71.9	5	5	0	1
F91.2	6	6	0	0
F92.0	11	11	0	0
F92.9	7	19	0	1
F98.0	5	5	0	1
G11.9	7	7	0	0
G13.8*	10	10	0	0
G20	150	326	0.000933	0
G25.8	24	31	0.000009	0
G25.9	47	56	0.00004	0
G26*	21	24	0.000006	0
G30.9	5	5	0	1
G31.0	87	139	0.000175	0
G31.1	9	9	0	0
G31.9	22	27	0.000002	0
G35	5	5	0	0

G40.40	8	8	0	1
G40.90	26	38	0.000005	1
G44.0	13	13	0	0
G44.8	33	53	0.000012	0
G45.0	63	90	0.000098	0
G45.4	13	13	0	0
G45.8	20	22	0.000006	12
G45.9	43	74	0.000034	0
G46.7*	10	10	0	0
G47.0	32	37	0.000009	0
G47.1	8	9	0	0
G47.30	163	523	0.000768	1
G47.31	89	155	0.000104	0
G47.32	230	833	0.001743	1
G47.33	26	36	0.000001	1
G47.39	22	31	0.000002	0
G47.8	13	13	0	0
G47.9	28	30	0.000007	0
G50.0	32	40	0.000012	0
G51.1	27	34	0.000008	0
G51.9	10	10	0	0
G54.0	8	8	0	6
G54.1	36	42	0.000012	0
G54.4	11	11	0	0
G56.0	13	13	0	0
G58.9	9	9	0	0
G62.0	16	16	0	1
G62.8	19	20	0	1
G62.9	22	24	0.000007	0
G70.0	26	28	0.000004	0
G70.9	14	14	0	0
G73.7*	18	30	0.000003	0
G81.9	119	243	0.000246	0
G83.2	10	10	0	0
G83.3	9	9	0	5
G83.8	5	5	0	3
G83.9	8	8	0	0
G90.2	8	8	0	6
G93.3	4	4	0	0
G93.4	26	30	0.000007	0
G93.9	34	56	0.000008	1
G96.8	41	51	0.000037	0
G96.9	25	25	0	0
G99.8*	10	10	0	0
H02.4	29	31	0.00001	0
H10.1	45	64	0.000014	1

H10.4	10	10	0	0
H10.5	15	15	0	1
H10.9	25	27	0.000004	1
H25.9	53	59	0.000046	0
H26.9	84	130	0.000252	2
H35.0	30	32	0.000008	0
H35.3	17	17	0	0
H35.4	14	14	0	0
H35.9	9	9	0	1
H40.0	5	5	0	1
H40.1	43	56	0.000013	1
H40.2	11	11	0	1
H40.8	16	16	0	2
H40.9	123	235	0.000617	0
H47.0	10	10	0	0
H52.0	11	11	0	1
H54.0	37	45	0.000015	1
H54.2	11	11	0	1
H54.4	10	10	0	1
H57.8	7	7	0	12
H60.9	15	15	0	0
H66.0	14	14	0	1
H66.9	9	9	0	0
H81.3	5	5	0	1
H81.4	28	35	0.000014	1
H81.9	48	128	0.000035	0
H90.0	41	57	0.000013	1
H90.1	10	10	0	1
H90.3	14	14	0	0
H90.4	5	5	0	1
H90.5	11	11	0	0
H90.8	8	9	0	1
H91.3	16	25	0.00001	1
H91.8	46	49	0.000024	0
H91.9	44	56	0.000008	1
H93.2	10	10	0	1
H93.8	10	10	0	0
I05.0	55	87	0.000024	1
I05.1	101	197	0.000312	0
I05.8	30	40	0.000008	0
I05.9	15	15	0	0
I06.0	9	9	0	0
I06.1	36	74	0.000036	0
I06.8	5	5	0	0
I07.1	216	682	0.001443	0
I07.2	24	27	0.000005	0

I07.8	19	20	0.000004	0
I07.9	27	32	0.000012	0
I08.0	39	65	0.000027	1
I08.1	38	55	0.000009	0
I08.9	11	11	0	1
I09.9	23	26	0.000003	0
I10	1081	13054	0.16709	1
I11.0	255	1145	0.00238	1
I11.9	127	294	0.000327	1
I12.0	7	7	0	1
I12.9	24	24	0	1
I15.0	19	19	0	0
I15.8	30	35	0.000005	0
I15.9	35	42	0.000019	0
I20.0	52	94	0.000024	1
I20.1	13	13	0	0
I20.8	181	493	0.00109	0
I20.9	225	883	0.001775	1
I21.4	16	16	0	1
I21.9	36	47	0.00002	1
I22.8	13	13	0	0
I22.9	35	62	0.000008	1
I23.3	38	39	0	0
I24.8	23	26	0.000002	0
I24.9	18	21	0.000001	1
I25.0	109	211	0.000263	0
I25.10	16	16	0	0
I25.11	40	64	0.000003	0
I25.2	217	770	0.001415	0
I25.3	16	16	0	0
I25.5	209	631	0.001431	1
I25.6	118	293	0.000313	0
I25.8	150	403	0.000739	1
I25.9	550	4100	0.021007	1
I26.0	32	36	0.000023	0
I26.9	92	176	0.000208	1
I27.0	245	1097	0.002302	1
I27.2	169	528	0.000772	0
I27.8	94	249	0.000197	1
I27.9	380	2480	0.006863	1
I28.8	10	10	0	1
I30.0	8	8	0	0
I30.8	20	22	0.000002	0
I30.9	35	44	0.000023	1
I31.3	31	37	0.00001	0
I31.8	13	13	0	0

I32.0*	7	7	0	1
I32.8*	23	23	0	0
I34.0	416	2071	0.009569	0
I34.1	27	45	0.000002	0
I34.2	21	24	0.000002	0
I34.8	31	48	0.000017	0
I35.0	66	132	0.000067	0
I35.1	209	577	0.001384	0
I35.2	28	31	0.000008	0
I35.8	22	33	0.000008	0
I36.0	42	71	0.000024	0
I36.1	247	899	0.002382	0
I36.8	12	12	0	0
I36.9	11	11	0	0
I37.0	5	5	0	1
I37.1	90	176	0.000139	0
I37.8	19	27	0.000003	0
I37.9	33	51	0.000018	0
I40.0	9	9	0	0
I40.1	14	19	0	1
I40.9	8	8	0	1
I42.0	121	264	0.00019	0
I42.1	16	16	0	0
I42.2	35	45	0.000018	0
I42.9	15	15	0	0
I44.0	47	54	0.000019	0
I44.1	12	13	0.000001	1
I44.2	17	19	0.000001	0
I44.4	24	36	0.000003	1
I44.7	101	178	0.000235	0
I45.0	150	488	0.000766	1
I45.1	98	219	0.000255	0
I45.4	12	14	0	1
I45.5	8	8	0	3
I45.8	15	18	0.000001	1
I45.9	16	16	0	0
I46.0	21	22	0.000005	0
I46.9	171	507	0.000712	0
I47.0	23	31	0.000003	1
I47.1	104	183	0.000238	1
I47.2	27	58	0	1
I47.9	67	92	0.00006	0
I48	330	1557	0.004851	1
I49.0	29	32	0.000007	0
I49.1	113	274	0.000223	1
I49.3	150	433	0.000479	1

I49.4	70	183	0.000105	1
I49.5	42	78	0.000024	0
I49.8	102	226	0.000104	1
I49.9	92	174	0.000098	1
I50.0	519	4106	0.01743	1
I50.1	18	19	0.000002	0
I50.9	338	1576	0.005721	0
I51.7	49	92	0.000064	0
I51.8	15	15	0	0
I51.9	35	43	0.000041	0
I61.9	8	8	0	0
I63.1	24	24	0	1
I63.4	11	11	0	1
I63.8	56	70	0.000028	0
I63.9	68	131	0.00009	1
I64	64	123	0.000033	1
I65.0	11	11	0	0
I65.2	53	95	0.00004	0
I65.9	18	21	0.000001	1
I66.1	16	16	0	0
I67.1	7	7	0	0
I67.2	27	30	0.000015	0
I67.4	15	15	0	1
I67.8	31	69	0.000008	1
I67.9	80	141	0.000125	1
I69.2	5	5	0	2
I69.3	115	191	0.000263	0
I69.4	41	78	0.000018	1
I69.8	25	30	0.000008	0
I70.0	151	335	0.000557	1
I70.20	61	101	0.000044	0
I70.21	23	28	0.000002	1
I70.24	7	7	0	1
I70.8	104	171	0.000278	0
I70.9	149	428	0.000534	0
I71.00	10	10	0	0
I71.01	31	31	0	0
I71.2	32	53	0.000013	0
I71.9	20	32	0.000008	1
I72.2	11	13	0	1
I73.0	24	27	0.000007	0
I73.1	17	17	0	0
I73.9	52	97	0.000065	0
I74.3	9	9	0	0
I74.4	34	36	0.00001	0
I74.8	10	10	0	1

I77.6	11	11	0	1
I77.8	25	28	0.000004	1
I77.9	47	53	0.000011	0
I79.8*	21	21	0	0
I80.0	8	10	0	1
I80.2	20	21	0.000007	0
I80.3	23	25	0	1
I81	11	11	0	0
I82.9	16	18	0.000002	0
I83.0	26	33	0.000009	0
I83.1	43	55	0.000034	1
I83.2	11	11	0	0
I83.9	108	302	0.000168	0
I84.0	29	31	0.000019	0
I84.5	25	27	0.000011	0
I84.9	23	24	0.000001	0
I85.9	14	21	0.000001	0
I86.0	6	6	0	1
I87.0	69	126	0.000074	1
I87.2	279	913	0.003323	0
I87.8	11	11	0	1
I88.8	18	18	0	0
I89.0	6	6	0	0
I89.1	13	13	0	1
I95.0	13	13	0	1
I99	19	22	0.000002	1
J01.0	47	67	0.000036	0
J01.1	12	12	0	0
J02.9	21	23	0.000003	0
J03.9	18	23	0.000003	0
J04.0	3	3	0	0
J04.1	3	3	0	0
J04.2	15	16	0.000001	1
J06.8	22	25	0.000001	0
J06.9	21	22	0.000003	0
J10.0	28	43	0.000003	0
J10.1	24	32	0.000006	0
J10.8	30	40	0.000013	0
J11.0	14	15	0	0
J11.1	14	18	0.000006	1
J11.8	11	11	0	0
J12.8	139	323	0.00056	0
J12.9	195	710	0.001383	1
J13	10	12	0	1
J15.0	76	173	0.000078	1
J15.1	74	171	0.000069	1

J15.2	42	58	0.000015	1
J15.3	10	10	0	1
J15.4	54	106	0.000059	1
J15.5	50	92	0.000045	1
J15.6	91	262	0.000102	1
J15.7	7	7	0	0
J15.8	368	2521	0.00788	1
J15.9	241	1270	0.002427	1
J16.8	7	7	0	1
J18.0	337	1426	0.006422	0
J18.1	46	58	0.000048	0
J18.8	139	279	0.000652	0
J18.9	423	2265	0.012808	1
J20.9	9	11	0	1
J21.9	15	17	0.000001	1
J22	34	37	0.000014	0
J30.0	57	75	0.00009	0
J30.1	108	273	0.000472	0
J30.3	36	47	0.000012	0
J30.4	207	612	0.00178	1
J31.0	124	403	0.000389	1
J31.1	3	3	0	0
J32.0	37	40	0.00003	0
J32.1	8	8	0	0
J32.4	18	18	0	1
J32.8	6	6	0	1
J32.9	92	180	0.000251	0
J33.0	14	14	0	1
J33.1	18	18	0	1
J33.8	23	25	0.000002	0
J33.9	56	93	0.000025	1
J34.2	44	77	0.000025	0
J34.8	15	19	0.000001	1
J36	10	10	0	0
J37.0	20	23	0.00001	1
J37.1	6	6	0	1
J38.00	16	22	0	1
J38.01	15	34	0	1
J38.02	10	18	0	1
J38.1	23	29	0.000011	1
J38.2	9	9	0	0
J38.3	13	13	0	1
J38.4	10	10	0	1
J41.0	37	67	0.000012	1
J41.1	8	12	0	1
J41.8	27	43	0.000003	1

J42	42	61	0.000044	0
J43.1	24	38	0.000003	1
J43.2	33	36	0.000028	0
J43.8	43	68	0.000014	1
J43.9	223	767	0.001703	1
J44.0	457	3774	0.01244	1
J44.1	314	1853	0.004993	1
J44.8	137	383	0.00053	1
J44.9	248	815	0.002742	0
J45	1265	18126	0.319823	1
J46	68	111	0.000302	1
J47	444	2838	0.01315	1
J60	4	4	0	1
J62.8	33	35	0.000002	0
J67.7	13	13	0	0
J67.8	10	10	0	1
J67.9	21	28	0.000003	1
J68.9	13	13	0	0
J69.8	10	10	0	0
J70.1	13	13	0	0
J80	19	20	0.000002	0
J81	71	104	0.000075	0
J82	22	29	0.000006	1
J84.0	30	35	0.000009	0
J84.1	363	1607	0.008427	1
J84.8	46	71	0.000054	1
J84.9	271	1081	0.002965	1
J85.1	39	63	0.000021	1
J85.2	36	36	0	0
J86.9	20	25	0.000013	1
J90	224	758	0.002215	1
J91*	79	115	0.000142	0
J92.0	105	298	0.000257	1
J92.9	254	1114	0.002111	1
J93.0	5	5	0	7
J93.8	7	13	0	1
J93.9	41	57	0.00001	0
J94.0	7	7	0	1
J94.1	51	85	0.000042	1
J94.2	16	16	0	0
J94.8	45	80	0.000034	1
J94.9	19	23	0.000001	1
J95.0	10	11	0.000001	1
J95.1	22	34	0.000007	1
J95.9	5	5	0	0
J96.0	723	7175	0.05115	1

J96.1	589	4906	0.028833	1
J96.9	37	47	0.000014	0
J98.1	92	149	0.000233	1
J98.2	11	11	0	0
J98.3	17	20	0.000001	1
J98.4	41	58	0.000035	1
J98.5	17	17	0	0
J98.6	22	31	0.00001	1
J98.8	65	125	0.000111	1
J98.9	28	46	0.000011	0
K04.7	10	10	0	0
K20	5	5	0	1
K21.0	101	157	0.000693	0
K21.9	152	357	0.001062	0
K22.4	8	8	0	3
K22.9	8	8	0	0
K25.6	19	19	0	0
K25.7	57	85	0.000043	0
K25.9	68	114	0.000086	0
K26.0	27	42	0.000007	1
K26.1	19	19	0	0
K26.4	9	9	0	0
K26.7	52	87	0.000028	1
K26.9	26	33	0.000008	0
K27.0	6	6	0	0
K27.7	12	12	0	1
K28.7	11	11	0	0
K28.9	12	46	0.000004	1
K29.1	26	30	0.00001	1
K29.3	10	10	0	1
K29.4	25	25	0	2
K29.5	137	280	0.000575	0
K29.7	116	289	0.000274	1
K29.8	8	8	0	1
K29.9	12	13	0	0
K30	110	289	0.000283	0
K31.1	8	8	0	0
K31.82	11	11	0	0
K31.88	14	14	0	1
K35.1	6	6	0	1
K38.9	15	15	0	0
K40.90	54	68	0.000029	2
K41.9	9	9	0	1
K42.9	78	126	0.000108	1
K43.1	36	36	0	0
K43.9	14	14	0	0

K44.0	24	25	0.000001	0
K44.9	130	313	0.000551	1
K45.8	35	50	0.000026	0
K46.9	45	51	0.000017	1
K50.1	14	14	0	0
K50.9	8	8	0	1
K51.0	24	27	0.000012	0
K51.9	7	14	0	0
K52.8	15	15	0	0
K52.9	12	14	0.000003	1
K56.0	36	36	0	0
K56.4	19	21	0.000005	0
K57.30	53	67	0.000048	0
K57.31	19	19	0	0
K57.32	13	13	0	0
K57.50	20	20	0	0
K58.0	43	57	0.000034	0
K58.9	50	59	0.000059	0
K59.0	55	113	0.000036	1
K59.1	59	69	0.000058	0
K62.1	20	20	0	0
K62.2	11	11	0	1
K62.7	25	25	0	2
K63.58	25	25	0	0
K70.0	15	15	0	0
K70.1	59	77	0.000045	0
K70.3	10	10	0	1
K70.9	85	216	0.000157	0
K71.0	17	23	0.000001	1
K71.1	29	32	0.000003	0
K71.2	73	98	0.000193	0
K71.6	7	7	0	1
K71.9	78	171	0.000057	1
K72.0	87	129	0.000219	0
K72.9	20	25	0.000003	1
K73.2	11	11	0	0
K73.8	16	24	0.000002	1
K73.9	11	11	0	0
K74.0	84	177	0.000117	1
K74.5	11	11	0	0
K74.6	64	87	0.000147	0
K75.0	11	11	0	0
K75.2	37	45	0.000023	0
K76.0	327	1176	0.005821	0
K76.8	21	31	0	1
K76.9	52	149	0.000061	1

K80.00	34	44	0.000016	0
K80.10	28	38	0.000006	1
K80.11	14	14	0	0
K80.20	146	332	0.000572	0
K80.21	29	34	0.000005	0
K80.80	41	51	0.000016	0
K80.81	20	22	0.000003	1
K81.0	9	10	0.000001	1
K81.1	33	35	0.000017	0
K81.8	11	11	0	1
K82.8	42	71	0.000009	0
K83.0	7	7	0	0
K83.5	80	169	0.000046	0
K85	6	6	0	1
K86.1	23	25	0.000004	0
K87.0*	12	12	0	0
K90.0	13	13	0	0
K90.9	6	6	0	1
K91.1	20	37	0.000002	1
K91.2	4	4	0	1
K91.5	41	52	0.000011	0
K92.0	36	36	0	0
K92.1	53	60	0.000021	0
K92.2	34	37	0.000004	0
L02.4	13	13	0	0
L03.10	8	8	0	1
L03.11	66	97	0.000094	0
L04.0	9	9	0	1
L08.8	11	11	0	1
L12.0	7	7	0	1
L20.8	11	20	0	0
L30.9	15	15	0	1
L40.0	70	111	0.0001	1
L40.8	10	10	0	0
L40.9	48	63	0.00005	0
L43.1	14	14	0	0
L50.0	30	34	0.000008	0
L50.8	14	14	0	0
L50.9	10	10	0	1
L56.1	7	7	0	0
L80	16	27	0	0
L87.0	7	7	0	1
L89	82	104	0.00008	0
L94.0	11	16	0.000002	1
L94.8	7	7	0	1
L94.9	4	4	0	1

L95.9	15	15	0	0
M05.10	7	7	0	10
M05.89	39	47	0.00001	0
M06.00	5	5	0	9
M06.40	8	8	0	0
M06.80	13	13	0	1
M06.89	7	7	0	1
M10.09	12	12	0	0
M10.30	7	19	0	1
M10.95	14	14	0	0
M13.0	64	89	0.000065	0
M13.94	16	16	0	0
M15.3	11	11	0	0
M15.8	5	5	0	9
M15.9	90	192	0.000209	0
M16.0	22	30	0.000008	1
M16.1	31	64	0.000007	0
M16.6	13	13	0	0
M16.9	104	186	0.00027	0
M17.0	61	108	0.000064	0
M17.1	7	7	0	1
M17.4	38	43	0.000019	0
M17.5	9	9	0	0
M17.9	77	139	0.000224	0
M18.9	11	11	0	1
M19.99	25	28	0.000005	0
M20.0	9	9	0	1
M23.20	13	13	0	0
M23.29	11	11	0	0
M32.9	36	40	0.000009	2
M34.0	10	10	0	0
M35.0	24	44	0.000009	0
M35.9	16	17	0	1
M40.00	27	29	0.000008	1
M40.04	16	19	0.00001	10
M40.09	37	52	0.000003	1
M40.15	11	11	0	0
M40.20	10	10	0	0
M40.24	8	8	0	1
M40.29	27	27	0	0
M41.00	11	11	0	0
M41.19	6	6	0	0
M41.30	13	13	0	0
M41.80	18	20	0.000001	1
M41.89	15	15	0	1
M41.90	13	16	0	1

M41.93	11	11	0	0
M41.94	30	33	0.000005	0
M41.99	11	11	0	1
M43.02	38	39	0	0
M43.04	18	19	0.000002	1
M43.05	14	14	0	1
M43.06	7	7	0	1
M43.09	16	16	0	1
M43.12	7	7	0	1
M43.15	18	18	0	0
M43.85	18	18	0	0
M45.09	13	15	0.000001	2
M46.46	19	19	0	0
M47.08+	10	10	0	0
M47.20	19	19	0	0
M47.22	21	29	0.000022	1
M47.80	103	262	0.000269	0
M47.82	140	304	0.000718	0
M47.83	14	14	0	0
M47.84	121	254	0.00035	1
M47.86	38	49	0.000014	1
M47.88	14	14	0	0
M47.89	97	167	0.000181	0
M47.90	70	158	0.000075	1
M47.91	7	7	0	0
M47.92	96	156	0.000265	0
M47.94	30	39	0.000016	1
M47.95	11	11	0	1
M47.99	61	104	0.000073	0
M48.97	10	10	0	0
M50.3	24	33	0.000006	0
M51.0	9	10	0	1
M51.2	17	28	0.000002	0
M51.8	31	45	0.000006	0
M51.9	72	140	0.000096	0
M53.86	17	38	0	0
M53.87	16	16	0	2
M54.4	23	36	0.000005	0
M54.84	14	14	0	0
M54.85	12	12	0	0
M54.86	5	5	0	9
M54.90	18	18	0	0
M62.08	21	21	0	0
M62.26	16	16	0	1
M67.96	15	15	0	0
M71.2	26	26	0	0

M72.0	9	9	0	1
M75.0	29	52	0.000003	0
M75.8	15	15	0	0
M77.3	9	9	0	0
M79.00	17	35	0.000001	1
M79.09	18	24	0	1
M79.19	26	26	0	0
M79.20	19	19	0	0
M79.26	11	11	0	0
M79.28	17	18	0.000003	1
M79.59	6	6	0	1
M80.90	18	18	0	0
M81.00	13	19	0.000003	1
M81.09	18	18	0	1
M81.89	4	4	0	1
M81.90	29	44	0.000039	0
M81.98	41	54	0.000018	1
M81.99	62	82	0.000128	0
M82.89*	10	10	0	0
M84.88	8	8	0	1
M85.40	18	18	0	0
M85.41	11	11	0	1
M87.00	15	15	0	0
M87.09	15	15	0	0
M99.2	16	16	0	0
N02.9	70	165	0.00007	1
N03.9	43	85	0.000037	0
N10	4	4	0	10
N11.1	21	26	0.000009	1
N11.9	6	6	0	1
N12	44	50	0.000016	0
N13.3	51	60	0.000042	0
N17.8	128	233	0.000359	0
N17.9	240	602	0.002026	0
N18.0	53	81	0.000014	0
N18.8	42	57	0.000031	0
N18.90	268	1042	0.002407	1
N18.91	26	32	0.000007	0
N19	28	35	0.000011	0
N20.0	107	218	0.000189	0
N20.2	26	33	0.000008	1
N20.9	69	98	0.000093	0
N21.0	16	18	0.000004	0
N21.9	12	12	0	0
N23	15	15	0	1
N25.8	12	12	0	0

N26	7	11	0	1
N27.9	14	14	0	0
N28.1	98	197	0.000189	1
N28.8	12	12	0	0
N30.0	78	129	0.000188	0
N30.8	12	21	0	0
N30.9	26	29	0.000002	0
N31.0	27	31	0.000003	1
N31.9	15	15	0	1
N35.8	17	20	0.000002	0
N35.9	8	8	0	1
N39.0	489	2875	0.017889	1
N39.81	38	42	0.000003	0
N39.88	6	6	0	1
N39.9	31	34	0.000006	1
N40	219	723	0.00168	1
N41.1	7	7	0	1
N43.3	9	9	0	0
N45.9	12	12	0	1
N60.0	29	37	0.000009	1
N60.9	12	12	0	0
N61	13	24	0	1
N62	10	10	0	0
N64.9	15	16	0	1
N72	15	15	0	0
N81.1	16	16	0	1
N85.8	22	25	0.000004	0
N89.0	8	8	0	1
N95.0	12	12	0	0
N95.9	8	8	0	1
N99.3	6	6	0	0
N99.4	12	12	0	0
N99.9	4	4	0	1
Q07.0	17	19	0.000005	0
Q24.4	13	13	0	0
Q27.3	20	22	0.000003	0
Q39.6	10	10	0	1
Q44.6	32	37	0.00001	1
Q61.0	25	26	0.000009	0
Q61.2	16	16	0	0
Q61.3	17	20	0.000002	1
Q61.41	8	8	0	0
Q61.8	13	13	0	0
Q61.9	13	13	0	0
Q64.72	13	13	0	0
Q76.49	11	11	0	0

Q79.0	14	14	0	0
Q87.18	10	10	0	0
Q89.31	6	6	0	0
R00.0	94	217	0.000196	1
R00.1	71	97	0.00011	0
R03.1	4	4	0	1
R04.0	39	57	0.00001	0
R04.2	209	652	0.001645	1
R05	32	41	0.000015	0
R06.1	12	12	0	1
R06.8	44	49	0.000006	0
R07.4	39	63	0.000009	1
R09.1	215	562	0.00199	0
R09.2	119	289	0.000253	0
R10.0	40	45	0.000016	0
R10.1	4	4	0	0
R11	10	11	0.000002	1
R16.0	13	13	0	0
R16.1	31	42	0.000014	1
R16.2	14	14	0	0
R17	11	11	0	1
R18	44	51	0.00002	1
R21	10	10	0	1
R26.2	12	12	0	1
R29.1	14	14	0	0
R29.89	7	7	0	12
R31	136	399	0.000476	1
R32	16	24	0.000001	1
R33	20	22	0.000002	1
R39.8	11	11	0	1
R40.2	50	57	0.000038	0
R41.0	12	12	0	0
R41.2	14	20	0	1
R42	103	181	0.000344	0
R49.0	54	86	0.000042	1
R49.8	12	12	0	1
R50.0	86	150	0.000129	0
R50.1	36	56	0.000016	1
R50.9	171	463	0.000643	1
R51	30	31	0.000004	0
R52.0	18	30	0.000001	0
R55	70	90	0.000107	0
R58	14	14	0	0
R59.0	140	288	0.000536	1
R59.1	21	25	0.000007	0
R59.9	56	78	0.000056	1

R60.1	22	22	0	0
R63.3	10	10	0	0
R63.4	10	10	0	0
R64	128	329	0.000342	1
R73	241	820	0.002295	1
R74.0	270	1035	0.002962	0
R74.9	15	15	0	0
R76.0	26	31	0	1
R77.0	58	77	0.000066	0
R77.8	31	38	0.000017	0
R77.9	61	73	0.000033	0
R79.0	65	96	0.00009	0
R79.9	23	33	0.000002	0
R80	19	19	0	0
R90.0	6	6	0	1
R91	77	127	0.00008	1
R93.0	6	6	0	0
R93.1	11	11	0	1
R94.2	23	28	0.000004	1
R94.5	85	145	0.000147	0
R94.6	13	13	0	0
S01.9	8	8	0	4
S04.3	12	12	0	0
S20.80	13	13	0	1
S22.32	19	23	0.000006	1
S22.40	54	95	0.000055	1
S22.42	13	13	0	0
S22.43	19	19	0	0
S27.6	9	9	0	1
S30.0	13	13	0	0
S35.4	19	19	0	0
S52.02	7	7	0	1
S52.10	5	5	0	1
S52.20	10	10	0	1
S52.50	7	7	0	1
S72.00	12	12	0	0
S78.9	20	20	0	0
S82.28	26	26	0	0
S92.0	13	13	0	0
S98.4	9	9	0	1
T10.0	4	4	0	1
T17.5	19	32	0.000003	1
T41.2	11	11	0	0
T45.5	20	22	0	1
T49.9	7	7	0	1
T50.9	8	8	0	1

T65.2	10	10	0	0
T67.1	13	13	0	0
T67.3	13	13	0	0
T78.4	84	183	0.000152	1
T78.9	16	16	0	0
T79.7	22	27	0.000001	0
T79.9	8	8	0	4
T88.7	61	134	0.000042	1
T88.8	43	63	0.00002	0
T89.02	5	5	0	11
T91.1	13	13	0	0
T91.2	37	58	0.000016	1
T91.9	20	20	0	0
T93.2	9	9	0	1
U07.1	253	1213	0.003028	0
U07.2	11	11	0	0
W80	13	23	0	1
X57	5	5	0	11
Y40.5	10	10	0	0
Y40.8	27	62	0.000003	0
Y43.4	14	14	0	0
Y44.2	82	146	0.000088	0
Y82.3	19	19	0	0
Y83.1	9	9	0	1
Y83.5	16	16	0	0
Y83.6	39	64	0.000046	0
Y84.1	12	17	0	1
Y84.6	24	29	0.000001	1
Y95	87	103	0.000088	0
Z01.5	3	3	0	0
Z03.0	20	29	0.000003	1
Z03.1	82	204	0.000165	1
Z11.1	2	2	0	1
Z11.5	307	1223	0.004631	0
Z11.9	9	9	0	1
Z12.2	42	54	0.000025	1
Z12.8	17	21	0.000005	0
Z20.9	22	23	0.000012	1
Z22.52	17	17	0	0
Z43.0	7	12	0	0
Z59.0	30	52	0.000007	1
Z60.9	8	8	0	1
Z71.3	130	295	0.000742	0
Z71.4	12	28	0	1
Z71.6	33	55	0.00001	1
Z71.8	21	27	0.000001	0

Z72.0	248	1144	0.002325	1
Z72.1	88	203	0.000153	1
Z72.4	13	13	0	1
Z75.10	16	28	0	0
Z82.1	12	12	0	1
Z82.3	24	30	0.000002	0
Z85.9	8	8	0	1
Z86.10	8	8	0	0
Z86.11	49	63	0.000018	2
Z86.43	25	25	0	0
Z87.12	25	25	0	2
Z87.18	16	16	0	0
Z88.0	45	52	0.000009	0
Z88.1	33	38	0.000022	1
Z88.2	6	6	0	1
Z88.8	42	44	0.000014	0
Z88.9	14	28	0	0
Z89.0	19	19	0	0
Z89.9	8	8	0	0
Z90.1	5	5	0	7
Z90.4	8	8	0	1
Z90.5	3	3	0	1
Z90.7	35	42	0.000011	1
Z90.8	18	18	0	1
Z92.1	206	630	0.001188	0
Z92.4	13	13	0	0
Z94.0	27	29	0.000005	0
Z95.0	99	200	0.000142	0
Z95.1	51	91	0.000027	1
Z95.2	19	22	0	0
Z95.5	68	125	0.000077	0
Z95.8	19	22	0.000001	1
Z95.9	26	27	0.00002	0
Z96.0	12	12	0	1
Z96.64	13	16	0.000001	0
Z96.8	10	10	0	0
Z97.4	13	13	0	0
Z98.8	20	22	0.000002	0
Z99.0	11	11	0	1
Z99.1	307	1884	0.003877	1

Group 70+

**Label**	**Degree**	**Weighted Degree**	**Betweeness centrality**	**Modularity class**
A02.0	15	18	0.000002	1
A04.1	9	9	0	5

A04.6	19	19	0	1
A04.7	278	967	0.002923	1
A04.9	98	195	0.000194	1
A07.1	46	65	0.000031	1
A08.0	11	11	0	1
A08.4	24	25	0.00001	1
A08.5	13	13	0	5
A09	119	274	0.000255	1
A15.0	134	274	0.000411	5
A15.1	76	121	0.000032	5
A15.2	6	10	0	5
A15.3	6	8	0	5
A15.5	17	18	0.000002	1
A15.8	12	12	0	1
A15.9	19	19	0	5
A16.0	25	32	0.000003	5
A16.2	12	12	0	1
A16.5	43	62	0.000006	5
A16.8	16	16	0	5
A18.1+	2	2	0	5
A18.2	10	10	0	5
A18.8+	23	26	0.000001	5
A41.0	10	10	0	1
A41.1	20	23	0.000001	1
A41.50	11	11	0	1
A41.58	13	13	0	1
A41.8	156	402	0.000425	5
A41.9	365	1445	0.005665	1
A46	44	55	0.000044	1
A49.0	47	68	0.000029	1
A49.9	10	10	0	1
A68.9	11	11	0	1
B02.2+	7	7	0	5
B02.8	5	5	0	1
B02.9	41	51	0.000035	4
B05.2	2	2	0	1
B05.8	4	4	0	5
B08.8	13	13	0	5
B17.1	4	4	0	5
B18.1	67	109	0.000079	1
B18.2	144	342	0.000631	1
B20	51	60	0.000028	1
B23.0	13	13	0	1
B23.8	11	11	0	1
B34.2	71	136	0.000094	1
B37.0	562	4005	0.01826	5

B37.1	200	910	0.00085	5
B37.2	9	9	0	1
B37.4+	13	13	0	5
B37.7	95	244	0.000136	5
B37.81	28	32	0.000004	5
B37.88	104	200	0.00017	1
B37.9	12	15	0	5
B44.1	53	71	0.000035	1
B44.9	19	21	0.000001	1
B47.9	10	10	0	5
B67.0	17	17	0	1
B67.4	16	16	0	3
B86	6	6	0	1
B90.2	9	9	0	5
B90.9	310	1414	0.003444	5
B94.8	9	9	0	7
B95.3	11	13	0	5
B95.5	25	30	0.000004	1
B95.6	135	267	0.000323	1
B95.7	16	22	0.000003	5
B95.8	23	25	0.000002	5
B96.0	6	6	0	1
B96.1	131	270	0.000218	1
B96.2	177	449	0.000791	5
B96.38	6	6	0	5
B96.4	32	40	0.000002	5
B96.5	127	313	0.000268	5
B96.81	11	11	0	5
B96.88	266	1871	0.002498	5
B97.1	9	9	0	5
B97.2	314	1434	0.003592	1
B99	54	79	0.000024	1
C18.0	30	46	0.000001	5
C18.2	15	15	0	5
C18.7	7	7	0	1
C18.9	38	53	0.00002	5
C20	34	42	0.00001	1
C22.0	9	9	0	1
C22.9	11	11	0	4
C25.0	6	6	0	5
C25.2	11	11	0	4
C25.9	8	8	0	5
C32.0	6	6	0	5
C34.0	41	59	0.000025	5
C34.1	83	188	0.000127	5
C34.2	14	16	0.000002	1

C34.3	13	15	0.000002	1
C34.8	44	73	0.000029	5
C34.9	162	413	0.000734	5
C38.4	16	32	0	5
C39.9	7	7	0	5
C45.0	13	15	0	5
C50.0	17	19	0.000002	5
C50.9	68	102	0.000102	5
C53.9	17	21	0	5
C61	36	43	0.000008	1
C64	16	46	0	5
C67.9	55	105	0.000029	5
C71.9	12	12	0	5
C73	15	22	0.000001	5
C74.9	11	11	0	1
C77.0	13	13	0	5
C77.1	14	14	0	5
C78.0	67	91	0.000139	4
C78.1	12	12	0	4
C78.7	46	62	0.000017	5
C78.8	11	11	0	5
C79.4	17	17	0	1
C79.5	23	25	0.000006	4
C79.7	18	18	0	5
C79.88	11	11	0	5
C80	34	38	0.000014	5
C82.9	12	12	0	5
C83.0	10	10	0	5
C83.1	14	14	0	1
C85.7	11	11	0	1
C85.9	16	16	0	1
C90.00	11	11	0	1
C90.20	11	11	0	1
C91.10	51	69	0.000013	0
C91.90	14	19	0.000002	1
C92.00	14	14	0	1
C94.70	8	8	0	1
D05.7	19	19	0	5
D09.7	11	11	0	4
D10.2	9	9	0	7
D12.5	14	14	0	1
D12.8	14	14	0	5
D13.4	15	15	0	1
D14.3	55	67	0.000027	1
D14.4	25	25	0	5
D15.1	6	6	0	1

D15.2	8	8	0	5
D15.9	11	11	0	5
D17.0	18	18	0	1
D17.4	13	13	0	5
D17.9	14	14	0	1
D18.01	7	7	0	5
D18.03	16	16	0	1
D18.08	22	25	0.00001	1
D25.9	9	9	0	5
D29.1	84	130	0.00009	1
D30.0	74	112	0.000043	1
D32.9	16	18	0.000001	1
D35.0	81	122	0.000088	1
D37.1	14	14	0	5
D38.1	61	90	0.000062	5
D38.2	11	11	0	5
D38.3	8	8	0	5
D39.9	13	13	0	5
D40.0	33	33	0	1
D41.0	6	6	0	5
D41.4	6	6	0	1
D43.0	16	16	0	1
D44.0	21	21	0	1
D44.1	6	6	0	1
D46.0	11	11	0	1
D46.3	20	20	0	1
D46.4	91	146	0.000211	1
D46.9	27	28	0.000015	1
D47.1	26	65	0.000002	5
D47.2	9	9	0	5
D47.3	17	17	0	1
D47.9	9	9	0	5
D48.6	21	21	0	1
D50.0	21	25	0.000002	1
D50.8	176	428	0.000982	5
D50.9	171	348	0.000769	1
D51.3	19	19	0	1
D53.0	21	28	0.000002	1
D53.1	7	7	0	5
D53.8	38	78	0.000024	5
D53.9	434	2440	0.008945	5
D58.9	11	11	0	1
D59.1	11	11	0	5
D61.9	26	29	0.000005	1
D62	17	17	0	5
D63.0*	56	105	0.000048	5

D63.8*	156	473	0.000513	5
D64.0	23	29	0.00001	5
D64.3	34	46	0.000006	1
D64.4	29	32	0.000011	1
D64.8	215	748	0.001234	5
D64.9	390	1745	0.00664	1
D68.8	39	60	0.000033	1
D68.9	40	57	0.000012	1
D69.0	9	9	0	1
D69.3	22	24	0.000001	1
D69.5	88	140	0.000158	1
D69.6	164	395	0.000664	1
D70	21	25	0.000009	5
D71	10	10	0	5
D72.0	31	37	0.000006	5
D72.1	66	143	0.000069	5
D72.8	185	552	0.000667	5
D72.9	72	112	0.000059	1
D73.5	25	28	0.000004	5
D73.9	18	19	0.000001	5
D75.1	23	32	0.000005	5
D75.2	138	311	0.000315	1
D75.8	22	24	0.000001	5
D75.9	16	16	0.000001	1
D76.1	10	10	0	5
D81.2	8	8	0	1
D81.9	53	70	0.000026	1
D82.9	9	9	0	1
D86.0	16	16	0	1
D86.2	7	7	0	5
D89.1	8	8	0	1
E01.1	44	52	0.000011	1
E02	28	32	0.000004	1
E03.8	41	52	0.000016	1
E03.9	172	430	0.000581	1
E04.0	40	43	0.000006	1
E04.1	116	191	0.000343	1
E04.2	38	43	0.000016	1
E04.9	26	31	0.000003	5
E05.0	11	11	0	1
E05.9	25	36	0.000004	5
E06.3	121	209	0.000354	1
E06.5	17	17	0	1
E06.9	22	23	0.000001	1
E07.8	34	41	0.000013	1
E07.9	12	12	0	4

E09.01	41	133	0.000012	5
E09.9	74	148	0.000044	5
E10.31	25	25	0	1
E10.40	53	60	0.000023	1
E10.42	12	12	0	5
E10.65	8	8	0	1
E10.69	10	10	0	5
E10.8	31	44	0.000007	5
E10.9	58	96	0.000034	5
E11.01	37	50	0.00002	1
E11.11	13	13	0	1
E11.13	9	9	0	1
E11.21	20	28	0.000003	5
E11.29	16	16	0	2
E11.40	29	32	0.000001	1
E11.41	28	28	0	1
E11.42	94	159	0.00009	1
E11.51	27	32	0.000006	1
E11.53	13	13	0	5
E11.65	70	132	0.000057	5
E11.69	82	125	0.000094	1
E11.72	33	33	0	1
E11.8	250	891	0.00162	1
E11.9	498	3781	0.012386	5
E13.01	22	22	0	1
E13.29	11	11	0	5
E13.31	10	10	0	5
E13.42	27	27	0	1
E13.65	11	11	0	1
E13.9	30	38	0.000017	1
E14.22	7	7	0	5
E14.32	33	33	0	1
E14.65	9	9	0	5
E14.8	8	8	0	1
E14.9	15	19	0.000001	5
E16.8	25	39	0.000002	5
E16.9	13	40	0.000001	5
E21.0	25	25	0	1
E21.1	24	24	0	1
E22.2	7	7	0	5
E25.9	8	8	0	5
E26.9	10	10	0	5
E27.9	24	49	0.000012	4
E44.0	8	8	0	5
E44.1	50	85	0.000017	5
E56.9	10	10	0	1

E58	16	16	0	5
E61.1	12	13	0.000006	1
E64.0	16	16	0	1
E66.0	411	2511	0.007564	5
E66.1	9	9	0	1
E66.2	51	99	0.000018	5
E66.8	97	202	0.000172	1
E66.9	243	780	0.001918	1
E71.3	20	21	0.000004	1
E72.5	19	19	0	1
E73.9	6	6	0	5
E75.0	22	22	0	1
E75.5	8	8	0	1
E75.6	40	55	0.000011	1
E77.9	16	16	0	5
E78.0	292	893	0.003047	1
E78.1	58	84	0.000051	1
E78.2	239	598	0.001881	1
E78.4	67	81	0.00004	1
E78.5	128	350	0.000253	5
E78.8	18	20	0.000005	1
E78.9	38	52	0.000009	1
E79.0	429	2169	0.007564	5
E79.8	23	23	0	1
E79.9	5	5	0	5
E80.2	6	6	0	1
E83.4	13	13	0	5
E83.5	25	28	0.000002	1
E85.9	10	10	0	5
E86	243	846	0.001739	1
E87.0	136	300	0.000376	1
E87.1	376	1461	0.005453	1
E87.2	220	706	0.001146	1
E87.3	118	219	0.000213	1
E87.5	228	699	0.001254	1
E87.6	413	1727	0.007626	1
E87.7	7	7	0	5
E87.8	318	1171	0.004975	1
E88.1	9	9	0	5
E88.2	33	42	0.000005	5
E88.8	27	44	0.000001	1
E88.9	130	264	0.000265	1
F00.0*	25	25	0	1
F00.1*	15	16	0	5
F00.2*	54	74	0.000044	1
F00.9*	49	64	0.000009	1

F01.1	17	17	0	1
F01.3	71	113	0.000041	1
F01.8	30	36	0.000009	1
F01.9	25	32	0.000012	5
F02.3*	8	8	0	5
F03	144	300	0.000219	1
F05.8	6	6	0	1
F05.9	10	10	0	5
F06.3	18	18	0	1
F06.4	18	21	0.000002	1
F06.6	235	792	0.002175	1
F06.7	93	137	0.00021	1
F06.9	21	21	0	1
F09	12	12	0	1
F10.2	61	76	0.000033	0
F11.8	8	8	0	5
F12.2	29	29	0	0
F17.2	93	183	0.000087	5
F20.0	8	8	0	5
F22.0	20	21	0.000003	5
F23.3	25	25	0	1
F25.2	11	11	0	4
F25.9	11	11	0	5
F29	26	29	0.000006	1
F31.3	78	152	0.00004	5
F31.4	24	27	0.000006	1
F31.6	5	5	0	5
F31.8	6	6	0	5
F31.9	13	14	0	5
F32.10	12	12	0	1
F32.90	12	19	0	5
F33.1	29	29	0	0
F33.2	8	8	0	5
F33.4	14	14	0	1
F33.8	79	148	0.000058	5
F33.9	136	272	0.000349	1
F39	12	12	0	5
F41.0	58	110	0.000045	1
F41.1	50	62	0.000021	1
F41.2	243	696	0.002132	1
F41.3	9	9	0	1
F41.9	145	374	0.000337	5
F42.9	8	8	0	5
F43.0	16	16	0	1
F43.9	11	11	0	5
F45.0	14	18	0	5

F48.0	35	76	0.000021	1
F48.8	13	13	0	1
F51.0	54	67	0.000022	0
F51.2	31	35	0.000005	1
F51.8	19	22	0.000003	1
F92.0	27	30	0	1
G04.2	27	27	0	1
G12.2	80	120	0.000122	1
G13.8*	17	17	0	1
G20	321	1088	0.004274	1
G25.8	49	63	0.000046	1
G25.9	29	43	0.000017	1
G26*	14	14	0	1
G30.0	12	12	0	5
G30.1	31	34	0.000006	5
G30.8	7	7	0	5
G30.9	80	158	0.000061	5
G31.0	207	424	0.001079	1
G31.1	26	27	0.000002	1
G31.8	5	5	0	1
G31.9	71	98	0.000038	1
G40.00	10	10	0	5
G40.80	7	7	0	5
G40.90	36	58	0.000014	5
G40.91	8	8	0	5
G43.3	8	8	0	1
G44.2	10	10	0	1
G44.8	33	34	0.000001	0
G45.0	113	259	0.000246	5
G45.1	13	13	0	1
G45.8	20	23	0.000002	5
G45.9	100	186	0.000186	1
G46.7*	28	39	0.000004	5
G46.8*	18	18	0	1
G47.0	23	25	0.000002	5
G47.30	127	277	0.000319	1
G47.31	24	33	0	5
G47.32	158	543	0.000414	5
G47.33	11	11	0	5
G47.9	36	48	0.000016	1
G50.0	18	18	0	1
G51.1	54	67	0.000041	1
G53.0*	7	7	0	5
G54.1	12	12	0	1
G54.4	12	12	0	1
G56.0	12	12	0	1

G62.8	42	68	0.000008	1
G62.9	16	16	0	1
G63.0*	6	6	0	5
G71.0	20	20	0	1
G80.01	13	13	0	1
G80.02	10	10	0	1
G81.9	142	285	0.000297	1
G82.54	7	7	0	1
G83.4	15	15	0	1
G83.8	18	18	0	1
G83.9	10	10	0	1
G92	38	57	0.000001	5
G93.3	26	43	0.000004	1
G93.4	77	108	0.000092	1
G93.9	97	155	0.000092	1
G96.8	56	79	0.000031	5
G96.9	11	11	0	1
G98	13	13	0	1
H05.2	10	10	0	5
H10.1	8	8	0	5
H10.9	4	4	0	5
H25.0	16	16	0	2
H25.2	14	14	0	1
H25.9	81	139	0.000197	5
H26.8	30	33	0.000007	1
H26.9	155	391	0.000513	5
H27.8	22	22	0	1
H27.9	15	37	0.000001	5
H35.9	9	10	0	5
H40.0	16	16	0	2
H40.1	67	118	0.00003	5
H40.8	25	27	0.000006	1
H40.9	124	314	0.000408	1
H53.4	28	28	0	1
H54.0	41	49	0.000019	5
H54.1	16	19	0.000002	5
H54.4	43	57	0.000016	5
H66.4	5	5	0	1
H66.9	11	13	0	5
H70.1	7	7	0	5
H80.9	7	7	0	5
H81.1	74	91	0.000071	1
H81.3	86	127	0.000137	1
H81.4	84	132	0.00006	5
H81.8	14	14	0	1
H81.9	96	153	0.000149	1

H90.0	169	412	0.000535	5
H90.1	26	37	0.000006	5
H90.2	19	19	0	5
H90.3	22	26	0.000001	5
H90.5	10	10	0	4
H90.6	12	12	0	5
H90.7	13	13	0	1
H90.8	58	89	0.000045	5
H91.1	29	34	0.000003	5
H91.3	5	5	0	5
H91.8	32	40	0.000009	1
H91.9	135	278	0.000244	1
H93.2	40	55	0.000011	1
I00	8	8	0	1
I05.0	44	52	0.00001	1
I05.1	66	116	0.000064	5
I05.2	43	52	0.000027	1
I05.8	74	123	0.000067	1
I05.9	26	32	0.000009	1
I06.0	21	25	0.000002	5
I06.1	36	57	0.00001	1
I06.9	13	13	0	1
I07.0	10	10	0	1
I07.1	379	1430	0.005209	1
I07.2	35	43	0.000005	1
I07.8	59	100	0.000035	1
I08.0	78	157	0.00006	5
I08.1	16	18	0	5
I08.9	11	11	0	5
I09.9	19	19	0	1
I10	1123	15709	0.153833	5
I11.0	293	1289	0.002957	5
I11.9	172	425	0.000629	5
I12.0	31	37	0.000018	2
I13.0	13	13	0	1
I15.1	17	17	0	5
I15.2	15	15	0	1
I15.8	19	19	0	1
I15.9	52	83	0.00005	1
I20.0	88	184	0.00008	5
I20.1	23	25	0.000006	2
I20.8	195	621	0.000906	5
I20.9	311	1134	0.003451	5
I21.0	25	34	0.000001	5
I21.1	7	7	0	5
I21.9	81	166	0.000124	1

I22.0	9	9	0	5
I22.9	27	31	0.000003	1
I23.2	22	22	0	1
I24.0	35	39	0.000006	1
I24.8	79	123	0.000106	1
I24.9	33	38	0.000003	1
I25.0	123	269	0.000259	1
I25.11	63	102	0.000042	1
I25.2	188	657	0.000585	5
I25.3	28	29	0.000001	1
I25.4	15	18	0.000004	4
I25.5	247	983	0.001893	5
I25.6	152	429	0.000432	5
I25.8	141	398	0.000432	5
I25.9	632	5130	0.027025	5
I26.0	63	93	0.00003	1
I26.9	138	278	0.000404	1
I27.0	344	1765	0.003865	5
I27.2	230	758	0.001205	1
I27.8	87	256	0.000094	5
I27.9	380	2576	0.004817	5
I28.0	13	13	0	5
I28.8	9	9	0	1
I30.0	10	10	0	5
I30.1	15	15	0	1
I30.8	10	10	0	1
I30.9	98	164	0.000082	1
I31.0	32	36	0.000008	1
I31.1	13	13	0	1
I31.3	42	50	0.000008	1
I31.9	13	13	0	5
I32.8*	18	21	0	5
I33.0	14	14	0	1
I34.0	534	2786	0.01364	1
I34.1	32	35	0.000002	1
I34.2	49	76	0.000009	5
I34.9	9	9	0	1
I35.0	150	381	0.000472	1
I35.1	305	1081	0.002581	1
I35.2	83	157	0.000144	5
I35.8	54	85	0.000034	1
I35.9	29	33	0.000006	1
I36.0	38	64	0.000005	1
I36.1	233	744	0.001272	1
I36.2	9	9	0	1
I36.9	17	18	0.000001	1

I37.1	97	188	0.000136	1
I37.8	17	17	0	1
I39.8*	27	27	0	1
I40.8	12	12	0	1
I42.0	176	511	0.00056	5
I42.2	17	17	0	1
I42.6	43	71	0.000003	5
I42.8	12	12	0	1
I42.9	45	59	0.000031	1
I44.0	105	178	0.000186	1
I44.1	18	21	0.000001	1
I44.2	43	50	0.000019	1
I44.3	7	7	0	5
I44.4	54	98	0.000023	5
I44.5	9	9	0	5
I44.6	16	16	0	8
I44.7	191	483	0.001065	5
I45.0	215	721	0.00102	5
I45.1	124	276	0.000182	1
I45.2	14	14	0	1
I45.3	16	33	0.000001	1
I45.5	43	68	0.000018	1
I45.8	9	9	0	5
I45.9	88	145	0.000088	1
I46.0	33	36	0.000003	1
I46.9	288	1118	0.002872	1
I47.0	42	53	0.000013	1
I47.1	114	193	0.000195	5
I47.2	18	28	0	5
I47.9	60	105	0.000044	5
I48	553	4194	0.016484	5
I49.1	145	485	0.000339	5
I49.2	14	14	0	5
I49.3	143	533	0.000269	5
I49.4	65	108	0.000064	5
I49.5	32	43	0.000013	5
I49.8	132	302	0.000243	1
I49.9	77	131	0.000081	1
I50.0	656	7051	0.028137	5
I50.1	98	133	0.000186	1
I50.9	486	2640	0.01196	1
I51.6	27	27	0	1
I51.7	151	307	0.000429	1
I51.9	65	88	0.000039	1
I62.1	8	8	0	1
I63.0	4	4	0	1

I63.2	11	11	0	1
I63.3	9	13	0	5
I63.5	33	33	0	1
I63.8	166	304	0.000627	1
I63.9	192	478	0.000901	1
I64	138	409	0.000291	5
I65.1	6	6	0	5
I65.2	93	146	0.000121	1
I65.8	19	19	0	1
I65.9	9	9	0	5
I66.0	10	10	0	5
I67.1	4	4	0	1
I67.2	60	99	0.000032	1
I67.4	11	11	0	1
I67.8	128	299	0.000314	5
I67.9	137	373	0.000245	5
I69.1	4	4	0	5
I69.2	12	12	0	4
I69.3	146	273	0.000698	1
I69.4	59	89	0.000027	1
I69.8	41	64	0.000018	5
I70.0	222	573	0.001268	1
I70.20	39	47	0.00002	5
I70.21	59	89	0.000034	5
I70.23	25	25	0	1
I70.8	146	257	0.000455	1
I70.9	256	823	0.001825	5
I71.1	11	11	0	1
I71.2	20	28	0.000001	5
I71.9	81	118	0.000086	1
I72.9	10	10	0	1
I73.0	21	23	0.000001	5
I73.1	18	18	0	1
I73.9	91	171	0.000086	5
I74.8	11	11	0	1
I74.9	13	13	0	5
I77.0	19	19	0	1
I77.6	11	11	0	5
I77.8	44	48	0.000014	1
I77.9	35	45	0.000006	1
I78.0	27	27	0	1
I79.0*	10	10	0	5
I80.0	57	83	0.000048	5
I80.2	10	10	0	5
I80.8	24	25	0.000011	5
I80.9	35	51	0.000008	5

I82.2	11	11	0	6
I82.3	11	11	0	6
I82.8	36	42	0.000016	1
I82.9	32	34	0.000008	1
I83.0	30	38	0.000001	5
I83.1	44	57	0.000009	1
I83.2	47	75	0.000017	1
I83.9	138	319	0.000372	5
I84.2	28	31	0.000002	5
I84.9	39	47	0.000018	1
I85.9	11	11	0	1
I87.0	105	215	0.000162	5
I87.1	11	11	0	1
I87.2	328	1227	0.003527	1
I87.8	55	65	0.000025	5
I87.9	10	10	0	1
I88.8	12	13	0.000001	5
I89.0	9	9	0	1
I89.1	16	16	0	3
I89.8	17	17	0	1
I89.9	15	15	0	1
I95.0	8	8	0	5
I95.1	4	4	0	5
I95.2	17	17	0	1
I95.8	14	14	0	1
I99	31	47	0.00001	1
J00	10	10	0	5
J01.0	21	24	0.000001	1
J01.3	26	26	0	1
J01.4	17	32	0.000004	5
J01.8	8	8	0	5
J02.9	12	12	0	4
J03.8	6	6	0	1
J06.9	42	65	0.000042	5
J10.0	25	34	0.000003	1
J10.1	31	38	0.000002	1
J10.8	47	55	0.00005	1
J11.1	17	21	0.000001	5
J12.1	9	9	0	1
J12.8	124	327	0.000364	1
J12.9	153	486	0.000571	5
J13	6	6	0	5
J15.0	84	161	0.000095	5
J15.1	82	177	0.000092	5
J15.2	91	168	0.000124	5
J15.3	8	8	0	5

J15.4	57	129	0.000033	5
J15.5	54	117	0.00001	5
J15.6	116	315	0.000255	5
J15.7	13	13	0	5
J15.8	446	3621	0.011706	5
J15.9	281	1594	0.003176	5
J16.8	11	11	0	1
J18.0	378	1867	0.005724	1
J18.1	43	57	0.000025	1
J18.8	174	471	0.000717	5
J18.9	443	2618	0.010305	5
J20.8	5	5	0	5
J20.9	24	26	0.000002	5
J21.9	2	2	0	5
J22	42	49	0.000018	1
J30.0	16	17	0	5
J30.1	46	82	0.00002	5
J30.2	6	6	0	5
J30.3	26	29	0.000001	1
J30.4	160	416	0.000772	5
J31.0	85	224	0.000279	5
J32.0	53	94	0.000017	0
J32.1	11	13	0.000001	5
J32.2	29	29	0	0
J32.4	24	37	0.000006	1
J32.9	52	75	0.000013	1
J33.0	7	7	0	5
J33.1	11	11	0	1
J33.9	27	38	0.000017	1
J34.2	19	19	0	1
J34.8	34	37	0.000001	5
J37.0	21	27	0.000002	5
J38.00	14	15	0.000001	5
J38.01	6	6	0	5
J39.9	3	3	0	5
J40	44	50	0.00002	1
J41.0	68	99	0.000043	1
J41.1	15	18	0	5
J41.8	12	12	0	5
J42	56	73	0.000059	1
J43.0	5	5	0	5
J43.1	30	33	0.000001	1
J43.2	32	39	0.000005	1
J43.8	70	117	0.000051	1
J43.9	243	913	0.001582	5
J44.0	483	3648	0.011717	5

J44.1	302	1977	0.003095	5
J44.8	138	410	0.000421	5
J44.9	207	736	0.001096	1
J45	1287	21422	0.272591	5
J46	84	169	0.000144	5
J47	456	2940	0.010231	5
J62.8	28	48	0.000012	5
J63.1	18	18	0	1
J67.8	5	5	0	1
J67.9	25	32	0.000005	0
J69.0	4	4	0	1
J70.4	20	20	0	1
J80	15	15	0	1
J81	40	72	0.000011	5
J84.0	6	9	0	5
J84.1	353	1767	0.004652	5
J84.8	74	138	0.000056	1
J84.9	304	1268	0.003426	5
J85.1	32	59	0.000007	5
J85.2	14	14	0	1
J86.0	4	4	0	1
J86.9	20	23	0	5
J90	259	1297	0.001868	5
J91*	79	138	0.000064	5
J92.0	138	495	0.000282	5
J92.9	269	1223	0.001995	5
J93.9	36	52	0.000011	1
J94.1	43	61	0.00002	5
J94.8	104	221	0.000219	5
J94.9	47	56	0.000054	1
J95.1	9	9	0	5
J95.8	9	9	0	5
J96.0	831	9950	0.061176	5
J96.1	633	5666	0.026851	5
J96.9	67	88	0.000054	1
J98.0	28	34	0.000003	1
J98.1	107	190	0.000104	5
J98.2	18	20	0	5
J98.3	34	54	0.000003	5
J98.4	41	49	0.000006	1
J98.8	41	74	0.000013	1
J98.9	22	22	0	5
K05.0	14	14	0	5
K09.2	26	26	0	1
K11.8	7	7	0	5
K20	26	29	0.000013	2

K21.0	124	282	0.000146	1
K21.9	80	128	0.000111	1
K22.0	25	31	0.000002	1
K22.3	10	10	0	5
K22.4	14	14	0	5
K22.5	34	51	0.000002	5
K22.8	20	20	0	1
K25.0	23	27	0.000002	1
K25.1	5	15	0	5
K25.3	11	11	0	1
K25.7	68	112	0.000034	5
K25.9	68	159	0.000051	5
K26.0	5	5	0	5
K26.7	45	63	0.000017	5
K26.9	29	37	0.000012	0
K27.3	11	11	0	5
K27.7	34	38	0.000007	1
K28.7	26	26	0	1
K28.9	14	14	0	1
K29.1	44	60	0.000018	5
K29.3	10	10	0	5
K29.4	25	36	0.000001	1
K29.5	95	167	0.000096	1
K29.7	149	448	0.00037	5
K29.9	61	95	0.000072	5
K30	108	237	0.000143	5
K31.88	12	12	0	1
K40.20	29	31	0.000003	1
K40.90	38	62	0.000007	5
K40.91	12	12	0	5
K42.9	94	158	0.000175	1
K43.9	34	46	0.00001	5
K44.0	33	41	0.000017	1
K44.9	261	675	0.002089	1
K45.8	22	26	0.000002	5
K46.9	48	86	0.000016	5
K50.9	11	11	0	5
K51.0	24	30	0.000004	5
K52.9	63	98	0.000075	1
K56.4	17	18	0.000001	1
K57.20	16	16	0	1
K57.30	72	93	0.000061	1
K57.31	9	9	0	0
K58.0	85	132	0.000106	1
K58.9	32	80	0.000002	5
K59.0	124	331	0.000301	5

K59.1	17	18	0.000001	5
K63.50	15	15	0	1
K63.9	30	40	0.000009	1
K65.9	9	9	0	1
K66.8	16	16	0	8
K70.3	19	19	0	1
K70.9	53	104	0.000026	5
K71.0	35	48	0.000019	1
K71.1	23	29	0.000002	5
K71.2	16	19	0	5
K71.6	19	22	0.000001	1
K71.7	8	8	0	1
K71.9	92	205	0.000081	5
K72.0	50	63	0.000009	1
K72.1	60	82	0.000036	1
K72.9	30	32	0.000001	1
K73.8	20	23	0	5
K73.9	19	23	0.000003	1
K74.0	37	76	0.000003	5
K74.4	10	10	0	1
K74.6	70	102	0.000081	1
K75.2	40	55	0.000021	1
K76.0	223	656	0.001516	5
K76.1	10	10	0	5
K76.2	14	14	0	1
K76.7	20	20	0	1
K76.8	45	51	0.000029	1
K76.9	35	49	0.000021	5
K80.00	35	41	0.000009	1
K80.10	47	60	0.000031	1
K80.20	192	449	0.000988	1
K80.21	24	26	0.000006	1
K80.50	25	27	0.000002	1
K80.80	60	86	0.000053	5
K81.0	29	34	0.000004	1
K81.1	34	41	0.000003	1
K81.9	22	27	0.000001	5
K82.8	8	8	0	5
K83.0	15	19	0.000001	5
K83.5	12	12	0	1
K83.9	5	5	0	5
K85	8	8	0	5
K86.1	12	15	0	5
K86.9	7	10	0	5
K91.0	11	11	0	5
K91.1	8	8	0	5

K91.4	12	12	0	1
K91.5	83	122	0.000069	5
K92.1	5	5	0	5
K92.2	48	60	0.000039	1
K92.8	26	33	0.000001	5
L03.11	64	97	0.000086	1
L03.9	12	12	0	5
L08.9	17	17	0	1
L12.0	27	27	0	1
L23.9	31	36	0.000005	5
L30.9	22	27	0.000001	5
L40.0	48	118	0.000015	5
L40.1	16	16	0	3
L40.9	47	62	0.000022	1
L50.8	9	9	0	1
L50.9	32	35	0.000005	5
L56.1	10	10	0	5
L73.9	12	12	0	5
L80	13	15	0	1
L82	10	10	0	5
L89	44	59	0.000019	1
L94.0	13	21	0	5
L94.4	15	15	0	1
L94.9	9	9	0	5
M05.00	9	9	0	7
M05.18	7	7	0	1
M05.39+	13	13	0	0
M05.80	19	22	0	5
M05.89	19	21	0.000002	5
M05.91	10	10	0	1
M06.40	9	9	0	5
M06.49	8	8	0	1
M06.90	10	10	0	1
M06.99	8	8	0	5
M10.09	9	9	0	1
M10.99	10	10	0	5
M13.0	60	95	0.000033	1
M13.90	18	18	0	1
M13.94	9	9	0	5
M15.9	161	490	0.000513	5
M16.0	77	103	0.000137	1
M16.2	6	6	0	1
M16.6	21	25	0.000004	5
M16.9	99	153	0.000224	1
M17.0	91	139	0.00007	1
M17.1	11	11	0	5

M17.4	9	12	0	5
M17.9	133	263	0.000387	1
M19.04	19	19	0	5
M19.08	7	7	0	5
M19.99	44	52	0.000008	1
M20.0	14	14	0	1
M32.9	17	17	0	5
M35.0	56	65	0.000038	1
M35.1	11	11	0	5
M35.9	14	14	0	5
M40.00	10	10	0	5
M40.03	29	34	0.000006	1
M40.04	33	41	0.000019	1
M40.09	90	172	0.000081	1
M40.14	46	51	0.000014	1
M40.20	26	34	0.000002	5
M40.24	45	66	0.000007	5
M40.29	65	101	0.000021	5
M41.03	18	18	0	1
M41.09	9	9	0	1
M41.19	20	21	0.000001	1
M41.35	2	2	0	5
M41.53	15	15	0	1
M41.54	12	12	0	1
M41.80	7	7	0	5
M41.84	40	54	0.000006	5
M41.85	31	33	0	1
M41.86	10	10	0	5
M41.89	36	53	0.000018	5
M41.90	11	11	0	5
M41.94	22	29	0.000004	5
M41.95	31	35	0.000012	1
M41.99	58	94	0.000033	1
M43.00	59	67	0.00003	1
M43.02	6	6	0	5
M43.03	21	21	0	1
M43.04	29	34	0.000004	1
M43.05	16	22	0.000002	5
M43.12	51	63	0.000027	1
M43.16	38	44	0.000011	5
M43.86	7	7	0	1
M43.96	25	25	0	1
M45.02	14	16	0.000001	5
M45.09	32	37	0.000016	1
M47.09+	7	7	0	1
M47.14	37	42	0.000006	1

M47.80	93	151	0.000147	1
M47.82	151	331	0.000728	5
M47.83	13	17	0.000001	1
M47.84	123	274	0.000294	5
M47.85	14	14	0	1
M47.86	55	74	0.000037	1
M47.87	13	13	0	5
M47.89	104	190	0.000164	1
M47.90	41	81	0.000005	5
M47.92	92	139	0.000166	1
M47.93	15	15	0	1
M47.94	20	23	0.000002	5
M47.95	36	39	0.000009	1
M47.99	50	72	0.00002	5
M48.06	8	8	0	5
M48.20	27	27	0	1
M48.50	34	38	0.000007	5
M48.92	13	13	0	5
M50.8	7	12	0	1
M51.2	35	45	0.000024	1
M51.3	23	29	0.000001	5
M51.8	11	11	0	0
M51.9	82	137	0.000172	1
M53.22	16	16	0	1
M53.83	8	8	0	5
M53.92	6	6	0	1
M53.97	17	20	0	5
M54.2	9	9	0	1
M54.4	95	181	0.000252	1
M54.86	16	16	0	1
M54.94	9	9	0	1
M62.39	10	10	0	1
M66.21	16	16	0	1
M77.2	16	16	0	1
M77.9	13	13	0	5
M79.00	15	17	0	5
M79.09	13	15	0	1
M79.20	17	17	0	1
M79.28	60	65	0.000042	1
M79.29	27	44	0.000001	1
M79.68	13	13	0	1
M80.00	16	16	0	2
M80.90	13	13	0	5
M81.00	46	93	0.000016	1
M81.09	29	34	0.000012	1
M81.90	43	56	0.000019	5

M81.98	92	138	0.000123	1
M81.99	18	19	0.000001	1
M87.07	25	25	0	1
M89.59	14	14	0	5
M93.2	8	8	0	5
M99.9	18	18	0	1
N02.9	71	147	0.000127	5
N03.3	21	21	0	1
N03.7	10	10	0	5
N03.9	16	17	0.000005	5
N04.0	7	7	0	5
N10	13	13	0	1
N11.0	16	16	0	8
N11.1	25	33	0.000004	5
N11.9	36	44	0.000016	5
N12	67	126	0.000043	5
N13.0	16	16	0	8
N13.3	49	59	0.000028	0
N17.8	147	305	0.000488	1
N17.9	261	1000	0.001838	1
N18.0	38	51	0.000021	1
N18.8	132	239	0.000228	1
N18.90	382	1988	0.005222	5
N18.91	59	87	0.000022	1
N19	42	54	0.000029	1
N20.0	145	281	0.00042	1
N20.2	39	61	0.000018	0
N20.9	118	225	0.000316	5
N21.0	44	53	0.000058	1
N25.0	24	85	0.000002	1
N25.9	8	8	0	1
N26	33	81	0.000006	1
N27.0	14	14	0	1
N28.1	182	402	0.000745	1
N28.8	18	32	0.000002	5
N28.9	34	39	0.000021	1
N29.8*	12	12	0	1
N30.0	140	243	0.000499	1
N30.9	24	26	0.000001	1
N31.9	10	10	0	5
N35.0	25	25	0	5
N35.9	12	12	0	5
N39.0	494	3718	0.01401	5
N39.2	15	15	0	1
N39.4	29	30	0.000003	1
N39.81	58	84	0.000017	1

N39.9	18	21	0.000001	1
N40	387	1829	0.005593	5
N41.2	13	21	0	5
N42.2	8	8	0	1
N51.0*	9	9	0	1
N60.0	17	17	0	1
N60.1	19	19	0	5
N60.9	19	19	0	1
N62	22	32	0.000001	1
N81.2	20	36	0	1
N82.0	11	11	0	0
N83.2	10	10	0	1
N87.1	8	8	0	1
N92.1	9	9	0	5
N95.3	16	16	0	8
N99.3	14	14	0	1
N99.9	10	10	0	5
O82	9	9	0	1
Q07.0	19	19	0	1
Q21.1	22	24	0.000003	1
Q21.2	8	8	0	5
Q32.0	16	16	0	1
Q32.2	16	16	0	1
Q32.4	4	4	0	5
Q33.1	10	10	0	5
Q40.1	58	104	0.000045	1
Q40.2	11	11	0	5
Q43.2	13	13	0	1
Q44.6	25	28	0.000004	5
Q45.89	4	4	0	5
Q54.9	17	17	0	5
Q61.0	52	72	0.00003	1
Q61.2	40	59	0.000008	5
Q61.41	74	120	0.000076	0
Q61.42	31	32	0.000003	1
Q61.8	46	59	0.000022	1
Q61.9	24	27	0.000003	1
Q89.01	22	22	0	1
Q89.24	10	10	0	5
Q96.8	14	14	0	5
R00.0	92	191	0.000125	5
R00.1	79	128	0.000104	1
R02	8	8	0	1
R04.0	66	77	0.000054	1
R04.1	13	13	0	5
R04.2	175	457	0.000861	5

R05	42	57	0.000021	1
R07.3	4	4	0	5
R07.4	9	9	0	5
R09.1	299	1070	0.002922	1
R09.2	224	725	0.001305	1
R10.0	29	43	0.000005	1
R10.4	11	11	0	1
R11	35	44	0.000022	1
R16.0	38	42	0.000022	1
R16.1	34	46	0.000022	1
R18	53	63	0.000061	1
R19.0	13	13	0	1
R21	15	17	0	5
R25.1	16	16	0	1
R27.0	9	9	0	5
R30.1	1	1	0	5
R31	125	416	0.000234	5
R32	47	68	0.000017	1
R33	11	11	0	5
R40.2	26	32	0.000009	1
R41.2	9	9	0	5
R41.8	28	29	0.000006	1
R42	157	362	0.0007	1
R45.1	24	24	0	1
R45.81	13	13	0	1
R47.0	31	34	0.000011	1
R49.0	49	89	0.000027	1
R49.2	12	12	0	5
R50.0	111	251	0.000304	5
R50.1	24	32	0.000002	1
R50.9	174	513	0.000688	5
R51	27	40	0.000008	1
R52.0	11	14	0	5
R52.1	7	7	0	5
R52.9	10	10	0	5
R53	16	16	0	5
R54	48	77	0.00001	5
R55	55	71	0.000042	5
R58	18	18	0	1
R59.0	136	239	0.000332	5
R59.1	18	19	0.000003	5
R59.9	65	110	0.000066	1
R60.9	12	12	0	1
R63.3	32	41	0.00004	1
R64	165	441	0.00064	5
R69	11	11	0	1

R72	25	27	0.000009	1
R73	316	1418	0.003906	5
R74.0	297	1134	0.002825	1
R76.8	14	14	0	1
R77.0	117	199	0.000356	1
R77.8	112	174	0.000288	1
R77.9	45	55	0.00001	1
R79.0	79	131	0.000121	1
R79.9	45	65	0.000011	5
R80	15	15	0	1
R82.7	16	18	0.000001	1
R90.0	8	8	0	5
R91	65	100	0.000032	1
R94.2	86	130	0.000109	5
R94.5	118	197	0.000244	1
S06.8	26	26	0	1
S14.5	14	14	0	1
S22.04	35	37	0.000003	1
S22.05	28	28	0	1
S22.32	36	53	0.000005	5
S22.40	41	48	0.000008	1
S22.42	32	35	0.000006	1
S25.3	16	16	0	3
S27.6	36	39	0.000004	5
S37.00	17	17	0	1
S37.30	23	23	0	1
S40.0	18	18	0	1
S40.88	18	18	0	1
S42.23	21	23	0	1
S43.01	11	11	0	5
S51.9	8	8	0	5
S52.30	12	12	0	5
S52.50	15	17	0	1
S72.00	33	45	0.000006	5
S72.10	7	7	0	5
S78.1	13	13	0	1
S92.9	11	11	0	5
S98.4	14	14	0	1
T17.5	12	12	0	5
T41.5	9	9	0	1
T45.5	90	129	0.000088	1
T51.0	17	17	0	5
T65.8	16	16	0	1
T78.4	71	156	0.00014	5
T78.8	27	37	0.000005	5
T80.1	13	13	0	5

T81.0	22	22	0	5
T82.2	11	11	0	4
T88.7	60	121	0.000016	5
T88.8	10	10	0	5
T89.02	30	30	0	1
T91.2	19	28	0.000001	5
T92.1	11	11	0	5
T96	13	13	0	1
T98.1	17	22	0.000001	5
U07.1	319	1477	0.003818	1
U07.2	51	61	0.000022	1
U09.9	50	64	0.000013	1
W18	15	15	0	1
W54.0	8	8	0	5
Y40.8	25	31	0.000012	5
Y44.2	49	105	0.000021	5
Y83.2	17	17	0	5
Y83.5	19	19	0	1
Y83.6	19	21	0.000002	5
Y83.8	9	9	0	5
Y84.6	20	24	0.000001	5
Y88.3	5	5	0	5
Y95	46	63	0.000007	1
Z03.0	6	6	0	5
Z03.1	91	235	0.000074	5
Z11.1	37	45	0.000004	5
Z11.5	380	1650	0.006343	1
Z11.9	7	7	0	5
Z12.2	27	36	0.000003	5
Z12.8	12	12	0	1
Z20.8	9	9	0	1
Z20.9	39	45	0.000011	1
Z22.3	12	12	0	1
Z22.51	11	11	0	1
Z22.52	9	9	0	5
Z45.0	11	19	0.000003	1
Z51.1	16	19	0	5
Z51.88	8	8	0	5
Z59.0	9	9	0	5
Z59.1	11	11	0	5
Z71.3	153	351	0.000907	1
Z71.7	13	13	0	1
Z71.8	46	58	0.000015	1
Z72.0	132	304	0.000324	5
Z72.1	25	28	0.000002	1
Z72.4	18	20	0.000005	5

Z73.0	9	9	0	5
Z74.0	14	14	0	5
Z82.3	14	14	0	1
Z85.2	14	14	0	5
Z85.3	30	45	0.000013	5
Z85.4	9	15	0	5
Z85.5	9	9	0	5
Z85.6	44	57	0.000007	1
Z86.0	9	9	0	1
Z86.11	6	6	0	5
Z87.12	18	23	0.000001	1
Z87.5	9	9	0	1
Z88.0	25	28	0.000007	1
Z88.1	65	87	0.000057	1
Z88.2	12	12	0	1
Z88.6	21	31	0.000006	5
Z88.8	24	24	0	1
Z89.9	18	18	0	1
Z90.2	22	24	0.000003	5
Z90.4	7	7	0	5
Z90.5	38	45	0.000005	5
Z90.7	20	23	0.000002	1
Z90.8	29	31	0.000007	1
Z91.0	19	22	0	5
Z91.1	9	9	0	5
Z91.6	9	9	0	1
Z92.0	8	8	0	5
Z92.1	322	1368	0.003168	5
Z92.4	6	6	0	1
Z92.9	10	10	0	5
Z95.0	92	223	0.000092	5
Z95.1	73	135	0.000068	5
Z95.2	38	51	0.000004	1
Z95.4	8	8	0	5
Z95.5	132	289	0.000294	1
Z95.8	14	14	0	5
Z96.2	7	7	0	5
Z96.4	13	13	0	1
Z96.64	77	117	0.000052	1
Z96.65	10	10	0	5
Z97.4	8	8	0	1
Z99.1	336	2068	0.003671	5
Z99.8	6	6	0	1
Z99.9	11	11	0	5
